# Lung and liver editing by lipid nanoparticle delivery of a stable CRISPR–Cas9 ribonucleoprotein

**DOI:** 10.1038/s41587-024-02437-3

**Published:** 2024-10-16

**Authors:** Kai Chen, Hesong Han, Sheng Zhao, Bryant Xu, Boyan Yin, Atip Lawanprasert, Marena Trinidad, Benjamin W. Burgstone, Niren Murthy, Jennifer A. Doudna

**Affiliations:** 1 Department of Molecular and Cell Biology, University of California Berkeley, Berkeley, CA, USA.; 2 Innovative Genomics Institute, University of California Berkeley, Berkeley, CA, USA.; 3 Department of Bioengineering, University of California Berkeley, Berkeley, CA, USA.; 4 Howard Hughes Medical Institute, University of California Berkeley, Berkeley, CA, USA.; 5 Gladstone Institutes, San Francisco, CA, USA.; 6 Gladstone-UCSF Institute of Genomic Immunology, San Francisco, CA, USA.; 7 Molecular Biophysics and Integrated Bioimaging Division, Lawrence Berkeley National Laboratory, Berkeley, CA, USA.; 8 Department of Chemistry, University of California Berkeley, Berkeley, CA, USA.; 9 California Institute for Quantitative Biosciences, University of California Berkeley, Berkeley, CA, USA.

## Abstract

Lipid nanoparticle (LNP) delivery of clustered regularly interspaced short palindromic repeat (CRISPR) ribonucleoproteins (RNPs) could enable high-efficiency, low-toxicity and scalable in vivo genome editing if efficacious RNP–LNP complexes can be reliably produced. Here we engineer a thermostable Cas9 from *Geobacillus stearothermophilus* (GeoCas9) to generate iGeoCas9 variants capable of >100× more genome editing of cells and organs compared with the native GeoCas9 enzyme. Furthermore, iGeoCas9 RNP–LNP complexes edit a variety of cell types and induce homology-directed repair in cells receiving codelivered single-stranded DNA templates. Using tissue-selective LNP formulations, we observe genome-editing levels of 16–37% in the liver and lungs of reporter mice that receive single intravenous injections of iGeoCas9 RNP–LNPs. In addition, iGeoCas9 RNPs complexed to biodegradable LNPs edit the disease-causing *SFTPC* gene in lung tissue with 19% average efficiency, representing a major improvement over genome-editing levels observed previously using viral or nonviral delivery strategies. These results show that thermostable Cas9 RNP–LNP complexes can expand the therapeutic potential of genome editing.

Clustered regularly interspaced short palindromic repeats (CRISPR)–Cas9-based genome editing^1–3^ has the potential to provide wide-ranging treatments for genetic diseases^4–6^ if safe and effective methods for delivering CRISPR-based therapeutics can be developed^7,8^. Although viral delivery of CRISPR genome editors is the most widely used method for in vivo cell editing^9–11^, viral vectors can be immunogenic, carry the risk of vector genome integration and can induce off-target DNA damage because of continuous genome editor expression^12^. Alternative nonviral strategies for delivering CRISPR editors could address these limitations if issues of efficacy and toxicity can be overcome.

Lipid nanoparticle (LNP)–mRNA complexes are nonvirally derived vehicles for in vivo delivery that have been remarkably successful at genome editing in the liver^13–15^. However, developing LNP–mRNA complexes that can edit nonliver tissues remains a challenge. Although LNPs can deliver mRNAs coding for Cre recombinase, luciferase and fluorescent proteins to nonliver organs, making the transition from reporter enzymes to CRISPR mRNA and single guide RNA (sgRNA) delivery has been inefficient^16,17^. LNP-mediated delivery of CRISPR mRNA and sgRNA faces challenges of sgRNA instability^18^, mRNA-mediated Toll-like receptor (TLR) activation^7^ and low translational efficiency of the large mRNAs encoding genome editors^19^. These challenges are inherent to the mRNA formulation but could be mitigated with alternative LNP delivery cargoes.

The direct delivery of genome editors in the form of ribonucleoprotein (RNP) complexes^20^ has the potential to address several of the limitations associated with mRNA-based and viral-based delivery of CRISPR editors. In particular, RNPs are expected to elicit lower levels of TLR activation than mRNA and produce minimal off-target DNA damage because of their short intracellular half-life^21–23^. In addition, RNPs may offer higher in vivo editing efficiency compared to mRNA-based delivery methods by avoiding in situ translation of large mRNA^19^ and providing natural protection of the sgRNA by high-affinity Cas9 binding^18^. Strategies for delivering RNPs include the use of complex polymers^24,25^, silica nanoparticles^26^, metal–organic frameworks^27^, LNPs^28–30^ and other formulations^31–34^. However, only LNPs have a proven track record of clinical use and established procedures for good manufacturing practice^35^. A successful LNP-based delivery strategy for RNPs, therefore, has great translational potential. Nevertheless, RNPs lack the negative charge density needed for efficient LNP encapsulation. Furthermore, conditions to formulate LNPs usually consist of organic solvents that can denature proteins^13^. Although LNP-mediated delivery of SpyCas9 in the RNP format induced genome editing in the liver^30^, delivery to nonliver organs such as the lungs remains inefficient^17,29^.

We rationalized that the protein denaturation problem currently limiting LNP-based RNP delivery could be tackled using alternative, thermostable CRISPR enzymes. The RNP of Cas9 from *Geobacillus stearothermophilus* (GeoCas9) has great potential for LNP-mediated delivery because of its higher thermal stability^36^ and higher negative charge density compared to commonly used editors such as *Streptococcus pyogenes* Cas9 (SpyCas9) or *Lachnospiraceae bacterium ND2006* Cas12a (LbCas12a). However, GeoCas9 has low genome-editing efficiency and uses a large protospacer-adjacent motif (PAM) that prevents it from editing a large fraction of the genome^36,37^.

In this study, we demonstrate that laboratory-evolved GeoCas9 mutants, iGeoCas9s, can edit mammalian cells with >100-fold higher efficiency than wild-type GeoCas9 and can edit cells and animal organs efficiently after LNP-mediated delivery. An LNP-based platform containing pH-sensitive PEGylated and cationic lipids enabled iGeoCas9-mediated editing of mouse neural progenitor cells (NPCs), human embryonic kidney 293T (HEK293T) cells and human bronchial epithelial (HBE) cells with efficiencies ranging from 4% to 99% depending on the locus. These iGeoCas9 RNP–LNPs could also induce homology-directed repair (HDR) upon codelivery with single-stranded DNA (ssDNA) templates in cells and efficiently edit the mouse liver and lungs after a single intravenous injection. For example, iGeoCas9 RNP–LNP formulations containing biodegradable ionizable lipids edited an average of 37% of the entire liver issue in Ai9 mice and also edited the *PCSK9* gene in wild-type mice with 31% efficiency, comparable to editing levels observed using other delivery systems^38^. In addition, iGeoCas9 RNP–LNP formulations containing acid-degradable cationic lipids edited an average of 16% of the entire lung tissue in Ai9 mice and were also able to edit the disease-causing *SFTPC* gene at 19% efficiency in the lungs, highlighting iGeoCas9 RNP–LNPs as a potential alternative to current delivery strategies based on viral or nonviral vectors^39–42^. Collectively, these results demonstrate that thermostable genome editors coupled with optimal LNP formulations can efficiently edit cells in vitro and in vivo and are a promising platform for developing CRISPR therapeutics.

## Results

### Directed evolution improves GeoCas9’s editing efficiency

GeoCas9 is a compact type II-C CRISPR–Cas9 protein that can function as a robust RNA-guided endonuclease at elevated temperatures (optimal temperature range of 50–65 °C) or in the presence of human plasma^36^. These properties make GeoCas9 an attractive editor for delivery in vivo, particularly in the RNP format. However, GeoCas9 is far less effective than the canonical SpyCas9 at genome editing in mammalian cells and has a more restricted PAM. Wild-type GeoCas9 recognizes a PAM sequence of 5′-N_4_CRAA-3′ (where R is A or G) and can consequently target a much smaller fraction of the genome than SpyCas9, which has a PAM sequence of 5′-NGG-3′.

**Extended Data Fig. 1 |:**
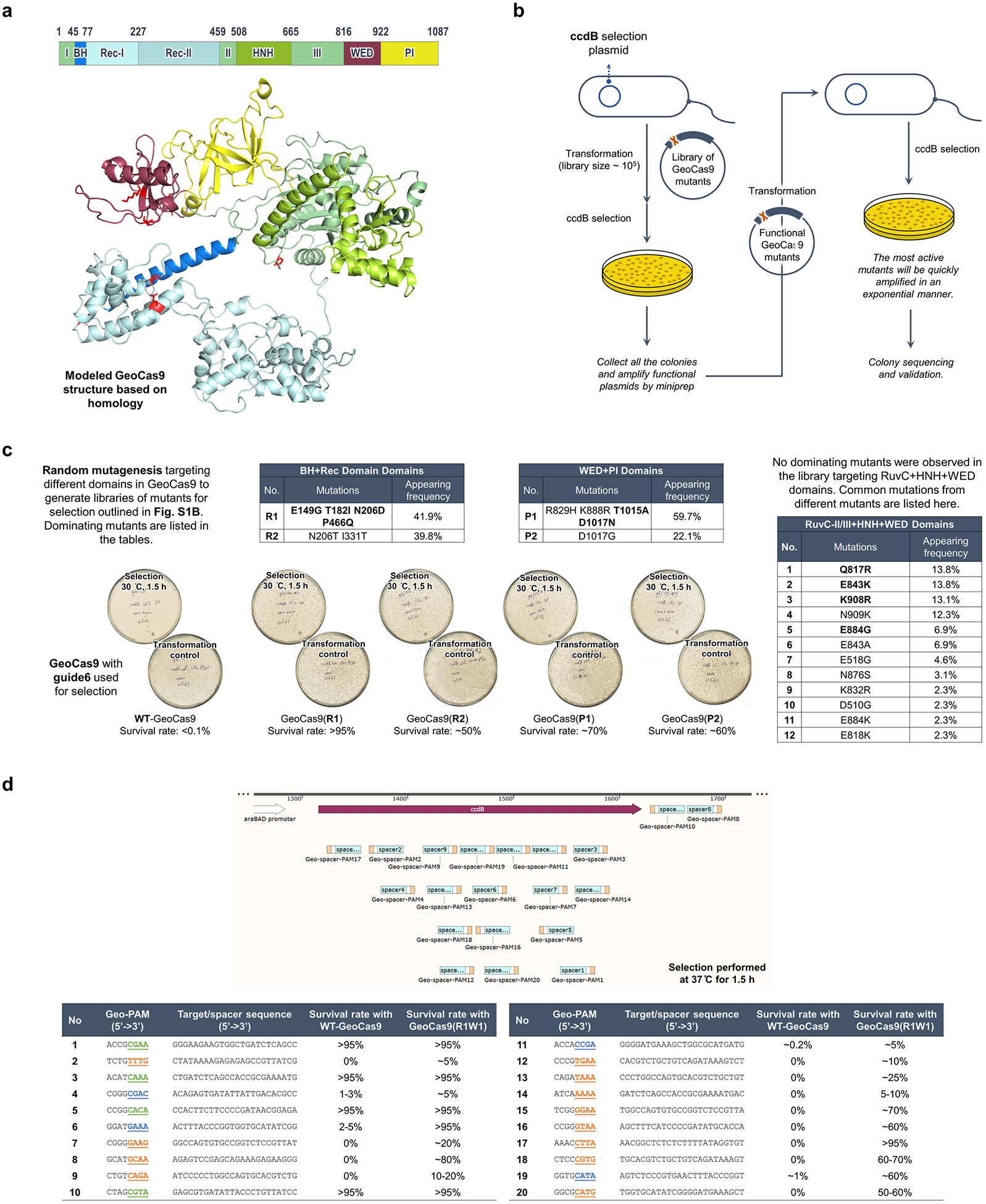
Directed evolution of GeoCas9. **a**. Modelled GeoCas9 structure with mutations highlighted. **b**. Schematic illustration on two sequential rounds of selection to identify improved GeoCas9 mutants. **c**. Mutants and beneficial mutations identified in each round of selection. **d**. Target cleavage activities of WT-GeoCas9 and R1W1 mutant in the bacterial assay using different spacer and PAM sequences, as reflected by the bacterial survival rates.

**Fig. 1 |:**
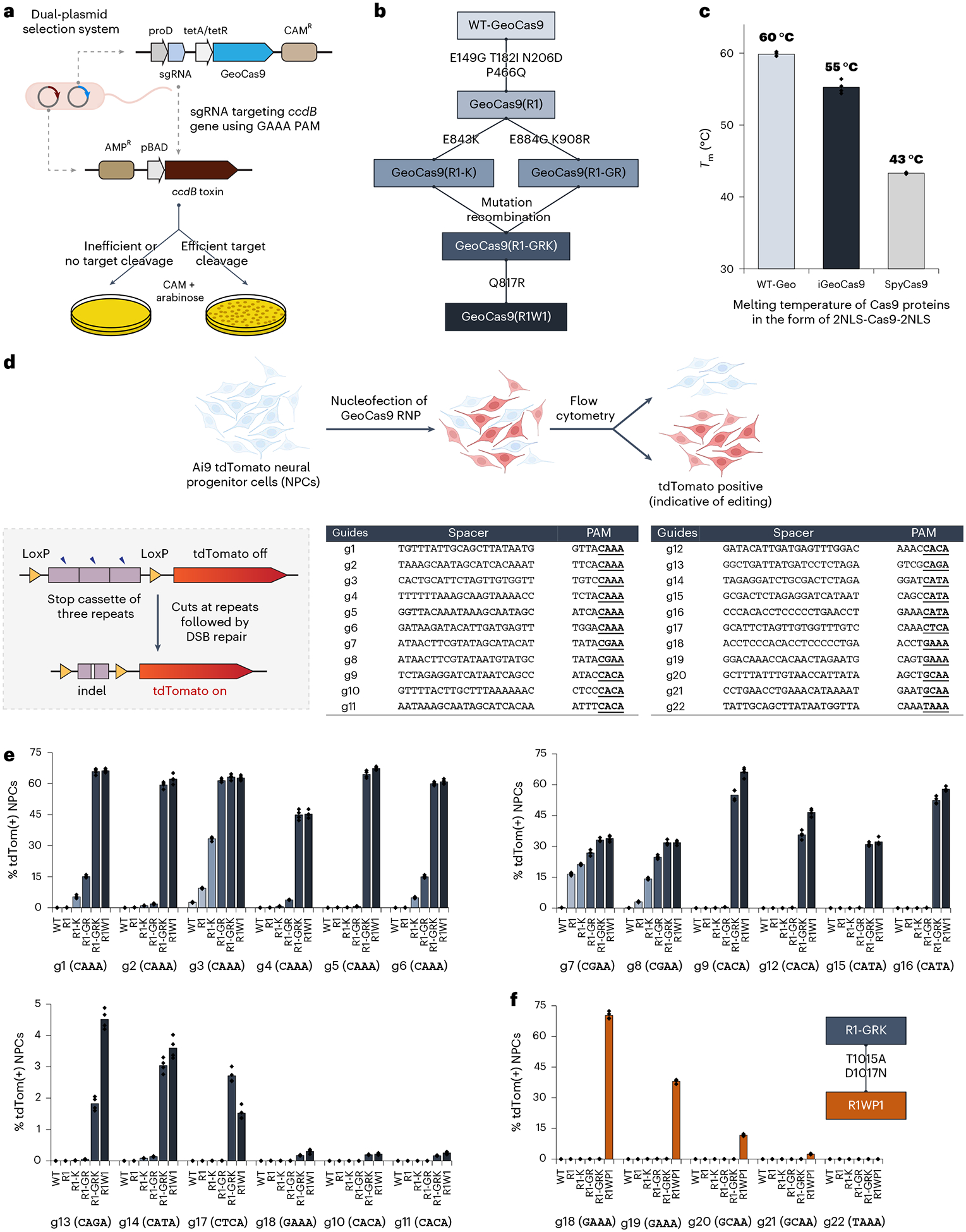
Directed evolution of GeoCas9 improves its editing efficiency by orders of magnitude and broadens its PAM compatibility. **a**, Schematic diagram of the direct evolution system used to evolve GeoCas9 based on bacterial selection. AMP^R^, ampicillin resistant. **b**, Evolutionary lineage of GeoCas9 mutants. **c**, Compared to the wild-type GeoCas9, evolved GeoCas9 (mutant R1W1) adequately preserved its thermostability with a melting temperature much higher than canonical SpyCas9. Melting temperatures of the three Cas9 proteins were measured by a thermal shift assay. **d**, Schematic diagram of GeoCas9-mediated genome editing of NPCs isolated from Ai9 mice. The spacer and PAM sequences of the GeoCas9 gRNAs were designed to turn tdTomato if successful editing occurs. Guides g7 and g8 target the LoxP sites for the stop cassette deletion. DSB, double-strand break. **e**, GeoCas9 mutants edit NPCs with significantly higher efficiency than wild-type GeoCas9 after electroporation-mediated delivery. Genome-editing efficiencies quantified based on tdTom(+) signals with the whole lineage of GeoCas9 mutants paired with different sgRNAs. **f**, PAM specificity is broadened through the further engineering of the GeoCas9 PI domain (*n* = 4 for each group); data are presented as mean values with individual data points.

We rationalized that directed evolution could be used to improve the editing efficiency of GeoCas9 and also minimize its PAM sequence requirement. A bacterial dual-plasmid selection system^43–45^ was used to select for improved GeoCas9 variants based on Cas9-mediated cleavage of a plasmid encoding the *ccdB* toxin gene under the control of an inducible pBAD promoter (Fig. 1a). By changing the Cas9 targets or altering the selection conditions, this targeted degradation of a toxin-encoding plasmid allowed adjustment of the selection pressure to enable directed evolution. To search for a reliable evolutionary starting point with minimal activity in the *Escherichia coli* (*E. coli*) assay, we screened 20 different sgRNAs that target the *ccdB* gene at the protospacers associated with different PAM sequences (Extended Data Fig. 1) and performed selection under two sets of conditions (37 °C or 30 °C for 1.5 h). Target sequence 6 with a disfavored PAM sequence (ggatGAAA) gave a minimal survival rate under either condition (<0.1% for 30 °C and 2–5% for 37 °C) and was chosen for engineering. Libraries of GeoCas9 mutants were generated by targeting different domains of the protein for random mutagenesis and then subjected to the selection system under the conditions at 30 °C (Extended Data Fig. 1). To amplify the most active mutants in these libraries, selected mutants were collected and subjected to another round of selection. Sequencing of the selected colonies identified frequently appearing beneficial mutations from each library. For instance, the library targeting BH + Rec domains for random mutagenesis generated mutant GeoCas9(R1) bearing four substitutions (E149G, T182I, N206D and P466Q), which gave >95% survival (versus <5% with the wild-type protein) in the bacterial assay. The addition of further beneficial substitutions identified in the library targeting RuvC + HNH + WED (wedge) domains, including E843K, K908R, E884G and Q817R, to the mutant R1 construct produced a new lineage of variant GeoCas9 proteins (Fig. 1b). Combining a total of eight beneficial substitutions yielded a composite mutant, GeoCas9(R1W1), which possessed greatly improved target double-stranded DNA (dsDNA) cleavage activity (Extended Data Fig. 1) and well-preserved thermostability (*T*_m_: 55 °C versus 60 °C for R1W1 mutant versus wild-type protein and 43 °C for wild-type SpyCas9) (Fig. 1c and Supplementary Figs. 1 and 2).

**Extended Data Fig. 2 |:**
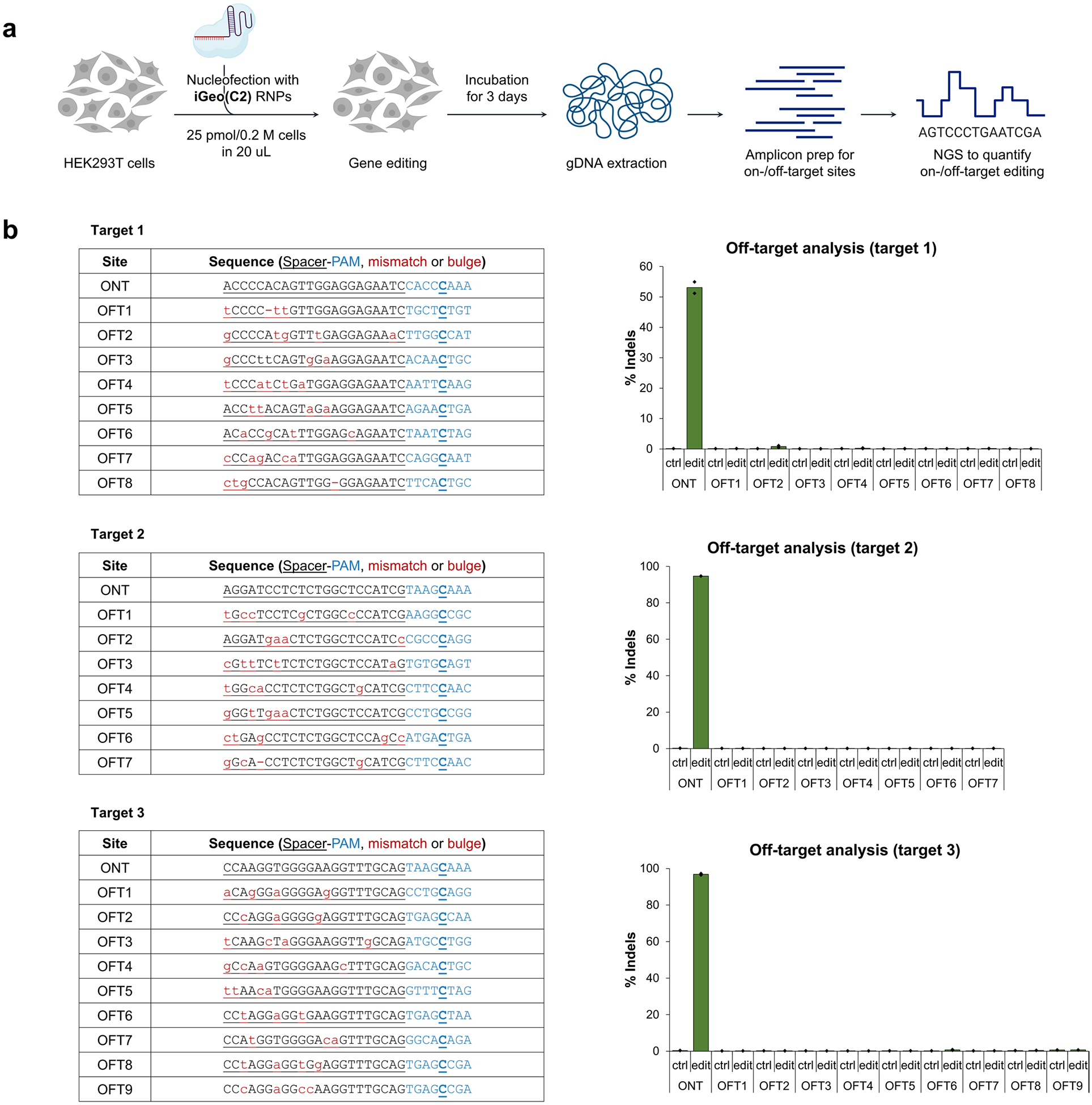
Off-target effect analysis for iGeoCas9-mediated genome editing. **a**. Schematic illustration on the analysis of on-target and off-target editing activities by iGeoCas9. **b**. On-target and off-target sequences listed in the tables, editing levels shown in the bar graphs. iGeoCas9 shows overall minimal off-target editing. Editing efficiencies quantified by NGS. n = 2 for each group, data are presented as mean values with individual data points. Target 1 = AAVS1 site1; target 2 = AAVS1 site2; target 3 = EMX1 site3. iGeoCas9 used in this figure is NLS-iGeoCas9(C2)-2NLS.

The genome-editing ability of the engineered GeoCas9 mutants was assessed in NPCs isolated from Ai9 tdTomato mice. In these cells, successful editing of a stop cassette sequence turns on tdTomato gene expression (Fig. 1d). A total of 22 sgRNAs were designed to target the SV40-derived poly(A) region using various PAM sequences. RNPs assembled from GeoCas9 mutants and these individual sgRNAs were electroporated into NPCs and the percentage of tdTomato-positive cells was determined by flow cytometry (Supplementary Fig. 3). The evolved mutants, GeoCas9(R1-GRK) and GeoCas9(R1W1), edited cells with >100-fold greater efficiency relative to the wild-type GeoCas9 with most sgRNAs investigated (Fig. 1e). In addition to editing NPCs, the evolved R1W1 mutant also exhibited robust genome editing in HEK293T cells and was able to reduce the expression of enhanced green fluorescent protein (EGFP) with up to 99% editing efficiency (Supplementary Fig. 4). These experiments demonstrate that the engineered GeoCas9 mutants can accept a broader range of PAM sequences, including but not limited to 5′-N_4_CNNA-3′ (versus wild-type PAM sequences: 5′-N_4_CRAA-3′) (hereafter GeoCas9(R1-GRK) and GeoCas9(R1W1) are referred to as iGeoCas9(C1) and iGeoCas9(C2) for improved GeoCas9 targeting C-based PAM sequences). Additional editing analysis further established that iGeoCas9(C2) is a highly efficient and precise genome editor with minimally detectable off-target effects (Extended Data Fig. 2).

To further expand the PAM compatibility of the engineered GeoCas9, the substitutions T1015A and D1017N identified from the library targeting the WED + PAM-interacting (PI) domains (Extended Data Fig. 1) were incorporated into a later variant in the engineering lineage, GeoCas9(R1-GRK), to create GeoCas9(R1WP1) (hereafter referred to as iGeoCas9(G)) that alters the preference of the first base in the essential 4-nt PAM sequence from C to G (Fig. 1f). Taken together, these results show that directed evolution can be used to engineer GeoCas9 for improved genome-editing activity and broadened PAM compatibility^46^.

### iGeoCas9 RNP formulated in LNPs edits cells in vitro

**Extended Data Fig. 3 |:**
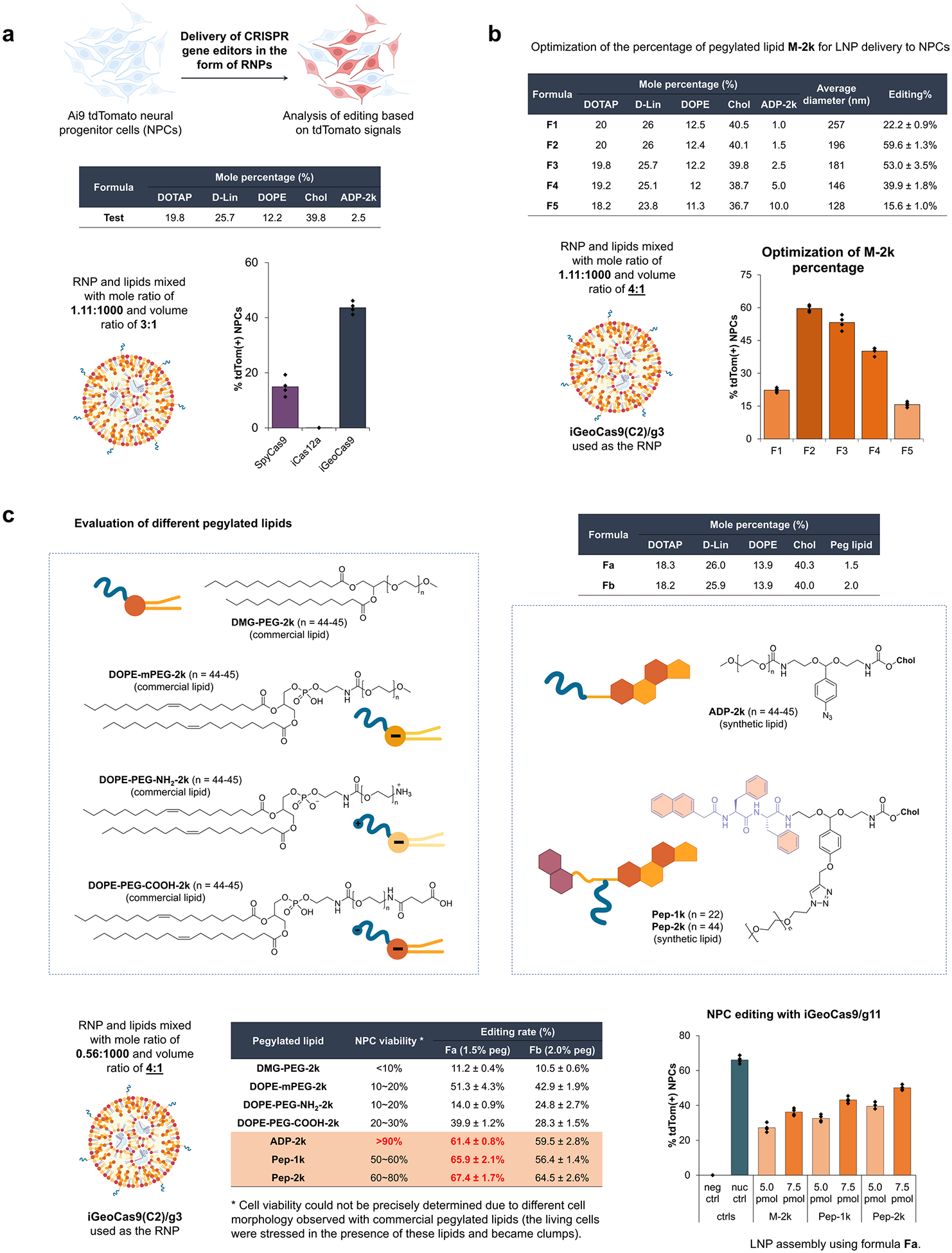
Optimization of LNP formulation for iGeoCas9 RNP delivery. **a**. Comparison of three different genome editors for Ai9 NPC editing based on RNP delivery by LNPs. **b**. Optimization of the percentage of pegylated lipid ADP-2k in LNP formulations. **c**. Comparison of different pegylated lipids in LNP formulations for iGeoCas9 RNP delivery efficiency and cytotoxicity with NPCs. n = 4 for each group, data are presented as mean values with individual data points. iGeoCas9 used in this figure is 2NLS-iGeoCas9(C2)-2NLS.

**Fig. 2 |:**
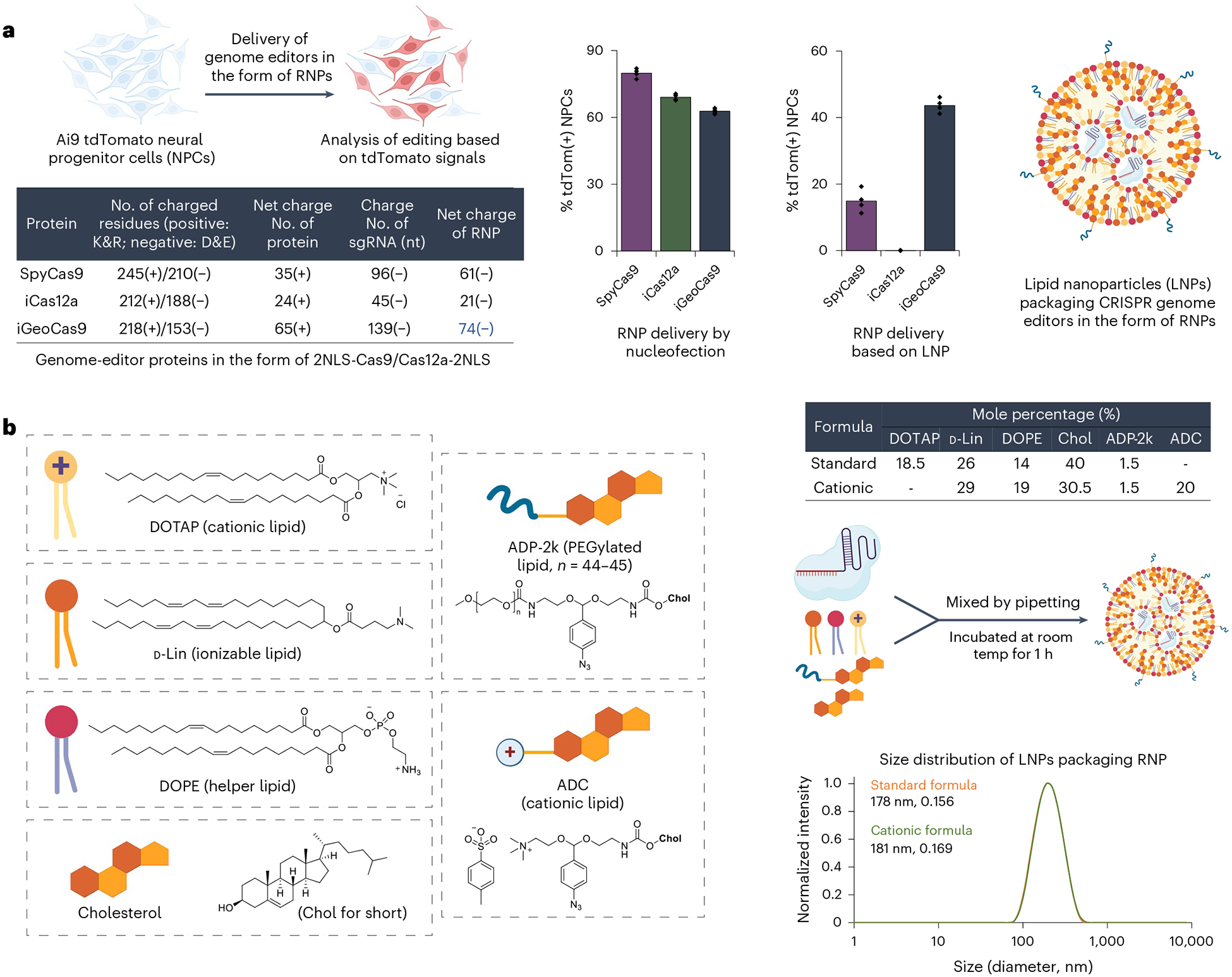
iGeoCas9 edits cells more efficiently than SpyCas9 or iCas12a after LNP-mediated delivery. **a**, iGeoCas9, SpyCas9 and iCas12a edit cells with similar efficiency after nucleofection. However, iGeoCas9 edits cells more efficiently after LNP-mediated delivery than either SpyCas9 or iCas12a (*n* = 4 for each group); data are presented as mean values with individual data points. **b**, Chemical structures of the lipids used in this study; two formulations were identified that delivered iGeoCas9 RNP efficiently, termed standard and cationic (details can be found in the table). DLS of standard and cationic LNPs demonstrates that they have sizes of 178 nm and 181 nm. iGeoCas9 used in this figure is 2NLS-iGeoCas9(C2)-2NLS.

The engineered iGeoCas9s have the potential to induce genome editing in cells and tissues that are not readily editable by other enzymes because of poor stability and/or limited delivery efficiency^17,29^. To test this, we compared the editing activity of iGeoCas9(C2) to that of two established genome editors, SpyCas9 and iCas12a, an engineered version of LbCas12a^45^ (Fig. 2a and Extended Data Fig. 3). Delivery by RNP nucleofection showed that all three of these enzymes generated robust and similar levels of genome editing in tdTomato NPCs. However, delivery of these RNPs using LNPs led to markedly different results: iGeoCas9 RNP–LNP delivery resulted in >2-fold higher editing efficiency compared to SpyCas9 RNP–LNPs and iCas12a RNP–LNP delivery did not produce detectable editing in these cells. The improved performance of iGeoCas9 RNP relative to other RNPs could be because of its higher stability and, thus, higher specific activity per LNP^13^. In addition, the larger size of the sgRNA for iGeoCas9 compared to SpyCas9 (139 versus 96 nucleotides) generates an RNP with increased negative charges, which could facilitate LNP encapsulation (Fig. 2a).

To set up a robust LNP-based system for iGeoCas9 RNP delivery, we further optimized the lipid formulation for RNP encapsulation and LNP assembly. We used four commercial lipids, including 1,2-dioleoyl-3-trimethylammonium propane (DOTAP), (6*Z*,9*Z*,28*Z*,31*Z*)-heptatriaconta-6,9,28,31-tetraen-19-yl 4-(dimethylamino)butanoate (d-Lin), dioleoyl-*sn*-glycero-3-phosphoethanolamine (DOPE), and cholesterol, and two synthetic lipids derived from cholesterol, ADP-2k and ADC, which are newly developed for mRNA delivery^47^ (Fig. 2b). The PEGylated lipid, ADP-2k, proved to be key to the successful encapsulation of RNPs into LNPs and delivery to NPCs (Extended Data Fig. 3). Low percentages (<1%) of ADP-2k led to relatively large particle sizes, which was not beneficial for LNP stability; on the other hand, high percentages (>5%) of ADP-2k resulted in smaller particle sizes but possibly inhibited the endocytosis processes resulting in reduced editing in NPCs. These observations correspond to the known behaviors of PEGylated lipids in enhancing LNP stability, controlling particle size and regulating circulation time^48,49^.

**Extended Data Fig. 4 |:**
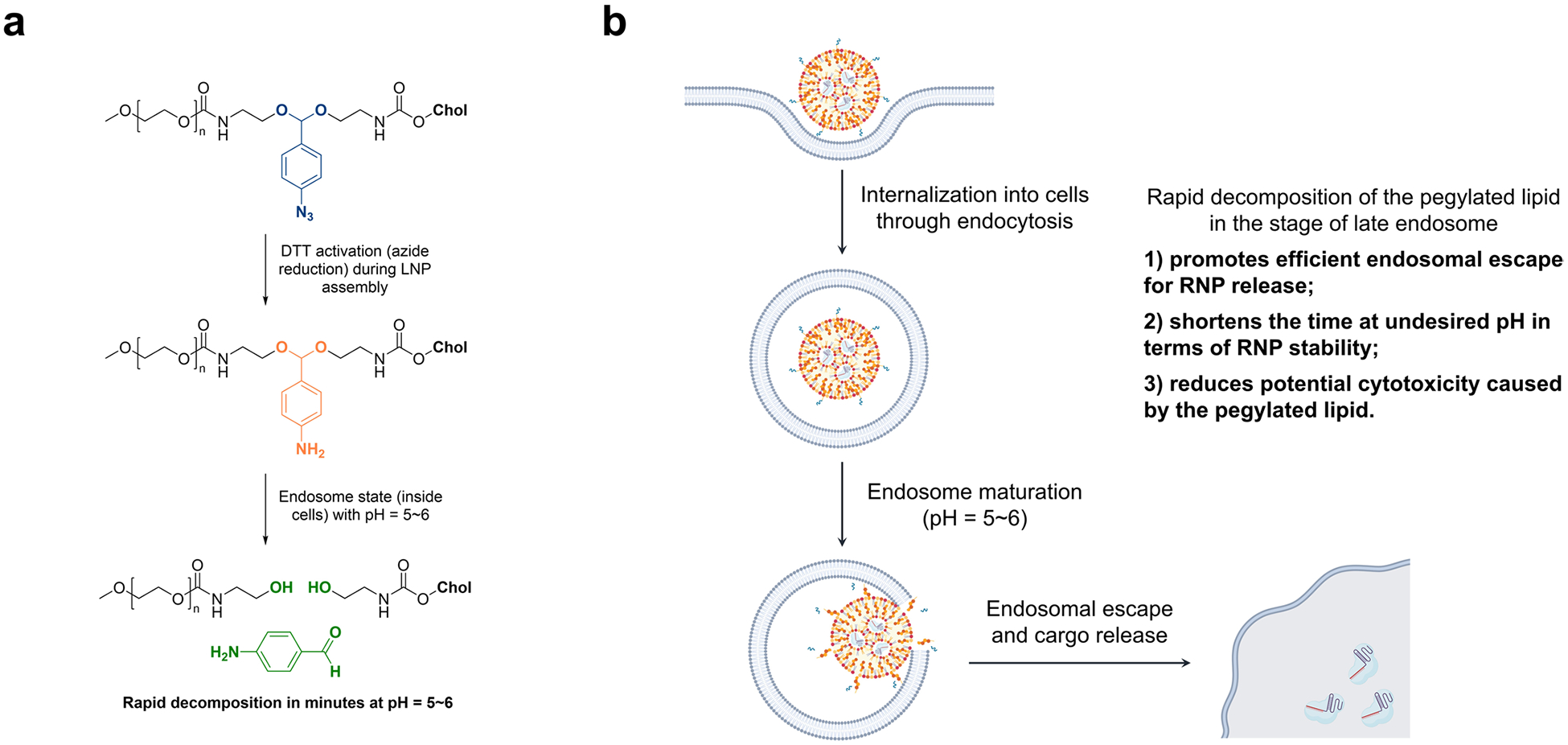
Mechanistic rationalization for promoted RNP delivery with acid-degradable lipids. **a**. pH-sensitive acetyl linker used in synthetic lipid design. **b**. Endocytosis pathway in LNP-based delivery promoted by the pH-sensitive acetyl linker in the lipids.

We next examined several PEGylated lipids, commercial and synthetic, for their ability to encapsulate and deliver iGeoCas9 RNPs in LNPs (Extended Data Fig. 3). The commonly used 1,2-dimyristoyl-*rac*-glycerol-methoxy(poly(ethylene glycol)) (DMG-PEG) and other PEG lipids derived from DOPE were found to be less effective in delivering RNPs while causing toxicity issues. Interestingly, the synthetic pegylated lipid ADP-2k exhibited minimal toxicity in NPCs and, with its inclusion in LNPs, we observed >90% cell viability. Two dipeptide-fused PEG lipids, Pep-1k and Pep-2k, also showed high editing levels in NPCs (Extended Data Fig. 3). The reduced toxicity and enhanced delivery efficiency of these three PEGylated lipids, ADP-2k, Pep-1k and Pep-2k, stem from the pH-sensitive, acid-degradable acetal linker used in their synthesis. Specifically, the labile acetal linker is cleaved in the late endosome stage of LNP delivery at a pH of 5–6, which frees the PEG moiety from the lipid molecule to reduce cytotoxicity while destabilizing the endosome to promote RNP release into the cytosol (Extended Data Fig. 4)^47^. Further optimization of other parameters of LNP assembly (including molar and volume ratios of lipids to RNP and salt concentration in the buffer; Supplementary Fig. 5) established two sets of lipid formulations, a standard formulation (with DOTAP as the cationic lipid) and a cationic formulation (with ADC as the cationic lipid). Both formulations can encapsulate iGeoCas9 RNPs and produce nanoparticles with sizes and polydispersity suitable for cellular delivery (diameter: 170–180 nm, polydispersity index (PDI): 0.13–0.17)^49^ (Fig. 2b).

**Fig. 3 |:**
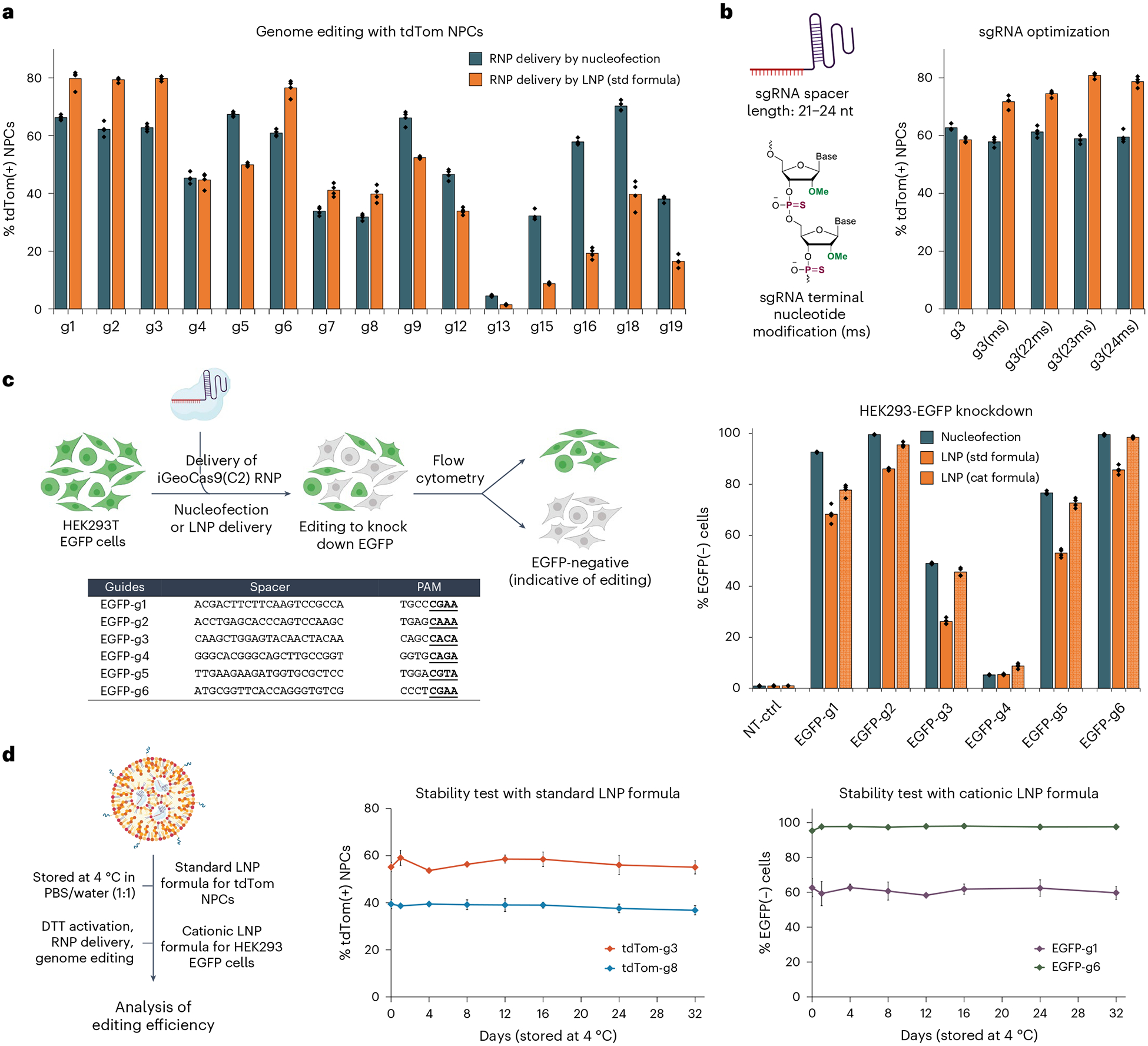
iGeoCas9 RNP–LNP complexes can edit a wide range of genomic targets and multiple different cell lines. **a**, LNP-mediated delivery of iGeoCas9 RNPs edits NPCs with efficiencies comparable to nucleofection. **b**, Chemical modification of sgRNAs improves the editing efficiency after LNP-mediated delivery (ms, 2′-methoxy and phosphorothioate linkage). **c**, Comparison of the genome-editing levels in HEK293T cells based on nucleofection and LNP-assisted delivery of iGeoCas9 RNP. Left, schematic diagram of iGeoCas9-mediated genome editing of HEK293T EGFP cells, resulting in the knockout of EGFP fluorescence. Right, genome-editing efficiencies quantified based on EGFP(−) signals using the engineered GeoCas9 paired with different sgRNAs (*n* = 4 for each group); data are presented as mean values with individual data points. NT-ctrl, non-targeting control. **d**, iGeoCas9 RNP–LNP complexes exhibit ultrastability, allowing for long-term storage in a neutral buffer at 4 °C. Left, schematic illustration of LNP stability test. Right, genome-editing efficiencies quantified based on tdTom(+) or EGFP(−) signals using iGeoCas9 RNP–LNP complexes stored at 4 °C for certain amounts of time (*n* = 4 for each group); data are presented as mean values with the s.d. iGeoCas9 used in this figure is 2NLS-iGeoCas9(C2)-2NLS except for editing experiments using 2NLS-iGeoCas9(G)-2NLS with g18 and g19 in **a**.

The genome-editing efficacy of iGeoCas9 RNP–LNP complexes was evaluated in NPCs by targeting the SV40-derived poly(A) stop cassette to turn on tdTomato. iGeoCas9 RNPs were assembled using corresponding sgRNAs and then encapsulated into LNPs of the standard formulation (Supplementary Fig. 6). Quantification of genome editing by sorting tdTomato-expressing cells after LNP treatment established that LNP-based delivery had comparable delivery efficacy to nucleofection (Fig. 3a). We next tested whether changes to the sgRNA could further enhance editing efficiency using the LNP delivery strategy. We extended the protospacer region from 21 nt to 23 or 24 nt and introduced 2′-*O*-methylation and phosphorothioate linkages to the last three nucleotides at both the 5′ and the 3′ ends (Fig. 3b). These chemical modifications, known to enhance the chemical stability of the sgRNA^18^, can also be beneficial to RNP delivery. The LNP strategy was also capable of delivering iGeoCas9 RNPs to HEK293T cells and disrupting the expression of an EGFP transgene with comparable efficiency to that observed using nucleofection (Fig. 3c). The cationic lipid formulation for LNP assembly was found to be slightly more effective for RNP delivery to HEK cells. In addition, the LNP–RNP complexes were stable and maintained high editing efficacy after storage in a neutral buffer (PBS and water, 1:1) at 4 °C for over 1 month (Fig. 3d). Together, these experiments established a robust LNP-based system for delivering iGeoCas9 RNPs to cell lines for genome editing.

### Codelivery of RNP and ssDNA induces site-specific integrations

**Fig. 4 |:**
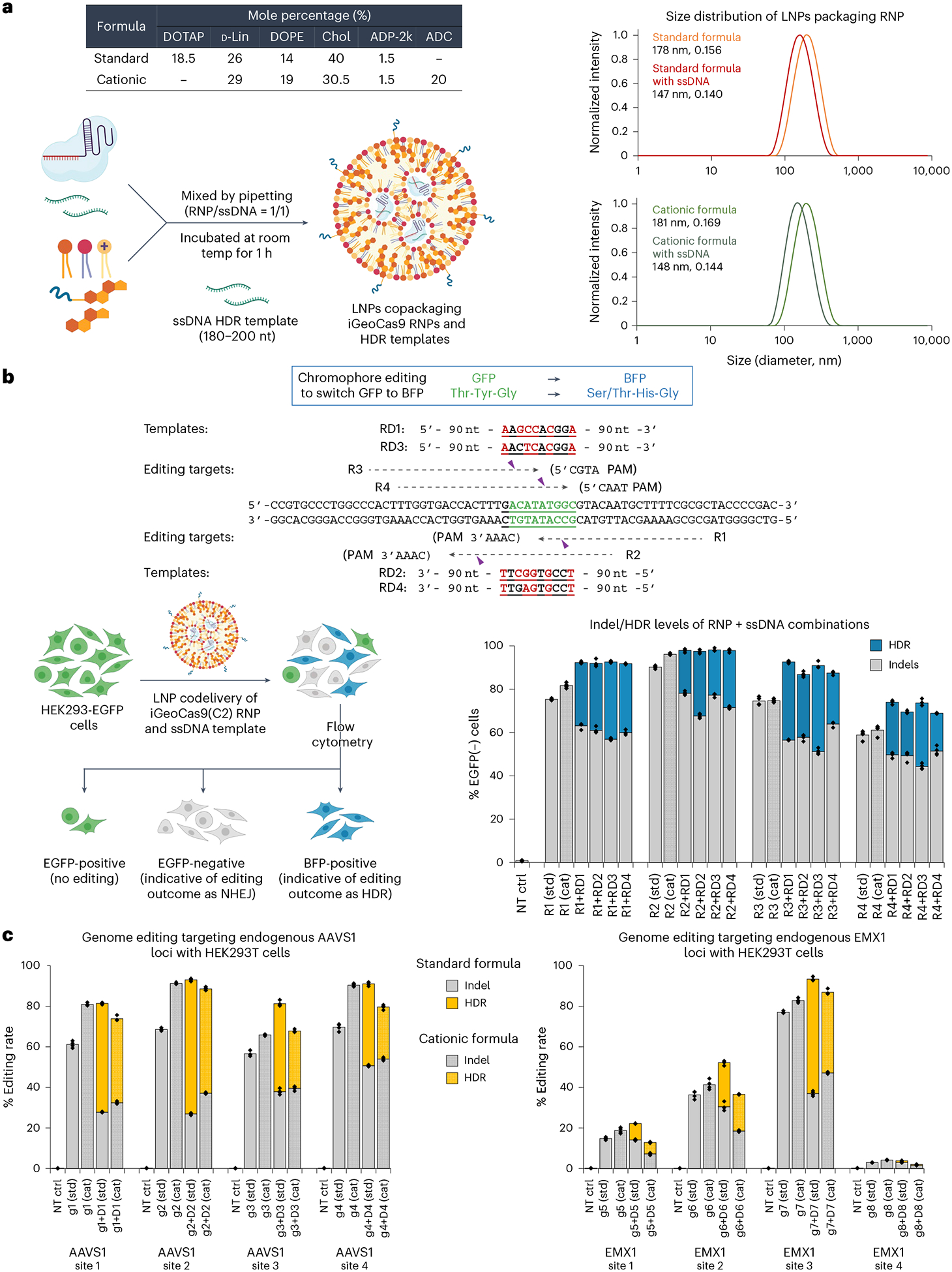
Codelivery of iGeoCas9 RNPs and ssDNA templates with LNPs efficiently generates HDR in cells. **a**, Characterization of LNPs encapsulating iGeoCas9 RNPs and ssDNA templates. **b**, Codelivery of iGeoCas9 RNPs and ssDNA HDR templates with LNPs edits the chromophore of EGFP to BFP in HEK293T cells. Top, target and donor designs for iGeoCas9-mediated chromophore editing. Bottom, genome-editing efficiencies quantified based on EGFP and BFP signals using iGeoCas9 paired with different sgRNAs ± ssDNA templates. iGeoCas9 RNP with ssDNA generates between 20% and 40% HDR in HEK293T cells. GFP, green fluorescent protein. **c**, Genome-editing efficiencies (indels and HDR) by iGeoCas9 paired with different sgRNAs ± ssDNA templates, as quantified by NGS (*n* = 4 for each group); data are presented as mean values with individual data points. iGeoCas9 used in this figure is NLS-iGeoCas9(C2)-2NLS. std, standard; cat, cationic.

We next tested whether LNPs can codeliver iGeoCas9 RNPs with an ssDNA template to induce site-specific genomic integrations through HDR. We first characterized the physical features of LNPs that copackage iGeoCas9 RNPs and ssDNA templates of 180–200 nt in length (Fig. 4a). Interestingly, in the presence of ssDNA (with a molar ratio of 1:1 for RNP:ssDNA), the nanoparticle size was reduced from ~180 nm to 140–150 nm. This phenomenon is consistent with a recent study showing that ssDNA helps RNP encapsulation into LNPs and prevents LNP aggregation by transient binding to Cas9 RNPs^30^.

We investigated whether the codelivery of iGeoCas9 RNPs and ssDNA templates in LNPs could switch EGFP to the blue fluorescent protein (BFP) in a model HEK293T cell line (Fig. 4b). In this cell-based assay, editing of the chromophore T-Y-G in the EGFP transgene by HDR installs an S/T-H-G chromophore and converts EGFP into BFP. Four sgRNAs, rEGFP-R1 to rEGFP-R4, were designed to target the coding and noncoding strands in the chromophore region for editing. To avoid possible recutting events after incorporation of the desired edits, four ssDNA HDR templates were designed to introduce GFP-to-BFP edits together with additional silent mutations in the DNA sequence. BFP signals were observed with the codelivery tests based on all 16 combinations of RNPs and ssDNA templates using the standard lipid formulation for LNP assembly. HDR levels, indicated by the percentage of BFP-positive cells, were quantified by flow cytometry and ranged from 20% to 40%, depending on the RNP + ssDNA combinations; non-homologous end-joining (NHEJ) levels were between 50% and 75% (Fig. 4b and Supplementary Fig. 7). Interestingly, the HDR experiments produced higher overall editing (HDR + NHEJ) levels compared to EGFP knockdown by RNP only, consistent with the role of ssDNA in promoting RNP encapsulation into LNPs. We wondered whether other anionic polymers, such as poly(l-glutamate) (molecular weight (MW): 15–50 kDa) and heparin (MW 10–30 kDa), could have similar effects (Supplementary Fig. 8). As expected, the anionic polymer poly(l-glutamate) also reduced the LNP size and modestly improved editing levels. However, the addition of heparin resulted in reduced editing, probably because of its inhibitory effect on Cas9 function. These results suggest that anionic polymers promote RNP packaging into LNPs through the charge interaction between the polymer additives and cationic lipids (Supplementary Fig. 8).

**Extended Data Fig. 5 |:**
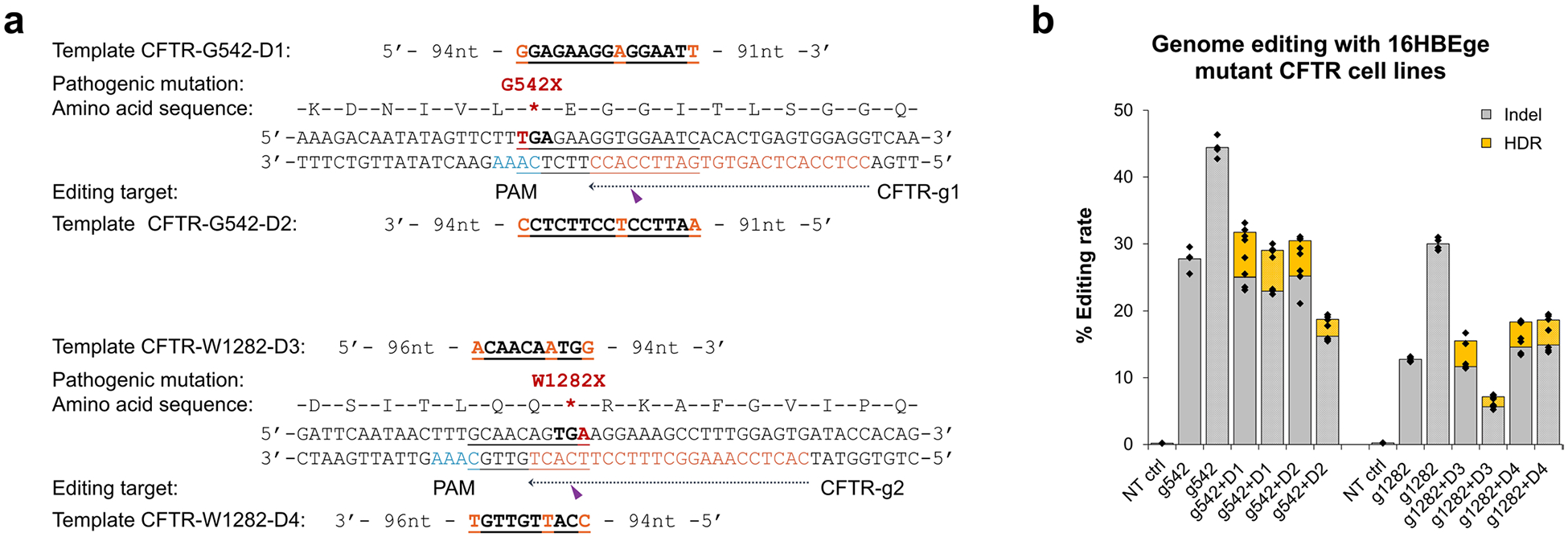
Editing of pathogenic mutations in the *CFTR* gene through HDR. **a**. Target and donor designs for iGeoCas9-mediated editing of pathogenic mutations. **b**. Genome editing efficiencies quantified by NGS. n = 4 for each group, data are presented as mean values with individual data points. iGeoCas9 used in this figure is NLS-iGeoCas9(C2)-2NLS.

LNP-based codelivery of iGeoCas9 RNPs and ssDNA templates was further used to induce HDR at endogenous genomic sites in human cells. Four sets of guide RNAs and corresponding donor ssDNAs were designed to target different sites in the *EMX1* gene and *AAVS1* locus, respectively, for genome editing based on HDR (Fig. 4c). Both standard and cationic LNP formulations were evaluated for their ability to deliver editing materials to HEK293T cells. HDR levels were quantified using next-generation sequencing (NGS), and LNP–RNP–ssDNA complexes generated up to 66% HDR, with total editing levels up to 95%. We then applied this codelivery system to cell lines of disease models and tested whether the LNP-based editing materials can correct pathogenic mutations. Cystic fibrosis is a genetic disease caused by mutations in the *CFTR* gene, which encodes the ion channel protein, cystic fibrosis transmembrane conductance regulator. Two HBE cell lines (16HBEge) containing nonsense mutations in the *CFTR* gene, leading to G542X and W1282X, were used for the HDR tests (Extended Data Fig. 5). iGeoCas9 RNPs and HDR donors were codelivered to the HBE cells, resulting in 7% HDR that reverted the pathogenic mutations leading to G542X and W1282X, as quantified by NGS. These results suggest that LNP-based RNP delivery may have therapeutic utility for restorative genome editing in the future.

### Specific ionizable lipids further improve editing efficiency

**Fig. 5 |:**
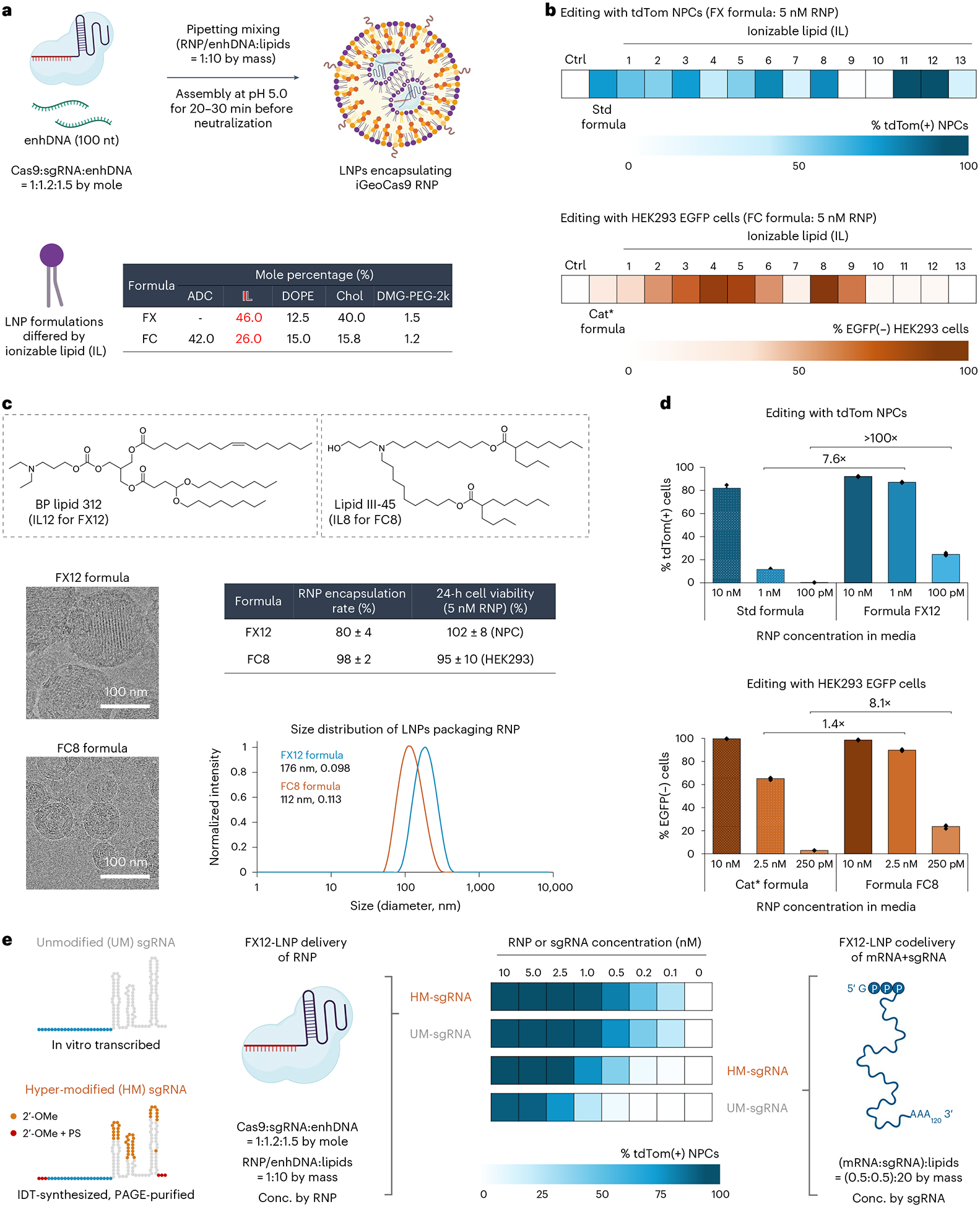
Rescreening of ionizable lipids dramatically boosts the delivery efficiency of iGeoCas9 RNP–LNPs assisted by enhDNA. **a**, Schematic diagram of procedures for LNP assembly and general lipid compositions of the two sets of LNP formulations (FX and FC) for ionizable lipid rescreening. **b**, Screening results indicate that ionizable lipids can dramatically affect the RNP delivery efficiency. Editing assays with tdTom NPCs (with tdTom-g3(23ms)) and HEK293 EGFP cells (with EGFP-g2) were used for the rescreening of FX and FC formulations, respectively. LP01 (IL11) and BP lipid 312 (IL12) were identified as the optimal ionizable lipids for the FX formulation and lipid III-45 (IL8) was identified as the optimal ionizable lipid for the FC formulation. The cationic* formulation used ADP-2k as the PEGylated lipid and d-Lin as the ionizable lipid based on the general FC formulation; standard and cationic* LNP formulations were assembled at pH 7.0. **c**, Characterization of microfluidic-formulated LNPs based on FX12 (FX with IL12) and FC8 (FC with IL8) formula. Top, chemical structures of IL12 and IL8. Bottom left, cryo-TEM imaging of FX12 and FC8 nanoparticles. Bottom right, DLS shows particle size distribution consistent with cryo-TEM imaging. The two formulations had good to high encapsulation efficiency for RNP cargoes and showed minimal cytotoxicity to cultured cells (NPCs and HEK293 cells; *n* = 4 for each group); data are presented as mean values ± s.d. **d**, FX12 and FC8 formulations show substantially improved efficiency for RNP delivery (with tdTom-g3(23ms) and EGFP-g6(23ms) as the sgRNAs) compared to the standard and cationic* formulations with different RNP dosages, even at subnanomolar RNP concentrations. Genome-editing efficiencies quantified on the basis of tdTom(+) or EGFP(−) signals using iGeoCas9 RNP–LNP complexes (*n* = 4 for each group); data are presented as mean values with individual data points. **e**, iGeoCas9 RNP–LNP delivery outcompetes mRNA+sgRNA–LNP delivery, especially with low cargo dosages. mRNA delivery is sensitive to sgRNA stability and requires hypermodification of sgRNA to enable successful editing at low mRNA and sgRNA dosage, while sgRNA modification does not affect the editing efficiency based on RNP–LNP delivery. iGeoCas9 used in this figure is 2NLS-iGeoCas9(C1)-2NLS. OMe, 2′-Omethyl; PS, phosphorothioate; Conc., concentration.

We performed an additional set of screening experiments to further optimize the iGeoCas9 RNP–LNPs, as our goal was to develop an RNP–LNP formulation with high efficiency, low cytotoxicity and low immunogenicity. The standard LNP formulation contains the acid-degradable lipid ADP-2k, which cannot be assembled at acidic pH, thus requiring the inclusion of the cationic lipid DOTAP (Supplementary Fig. 9). As DOTAP induces a strong immune response in mice^50^, LNP formulations lacking DOTAP could have notable advantages over the standard formulation. We, therefore, performed a screen to identify LNPs that could encapsulate iGeoCas9 RNPs without DOTAP. Two general formulations, FX and FC, were developed for the LNP screening; they contained an enhancer ssDNA (enhDNA), an ionizable lipid and DMG-PEG instead of ADP-2k and were formulated at pH 5.0 (Fig. 5a).

**Extended Data Fig. 6 |:**
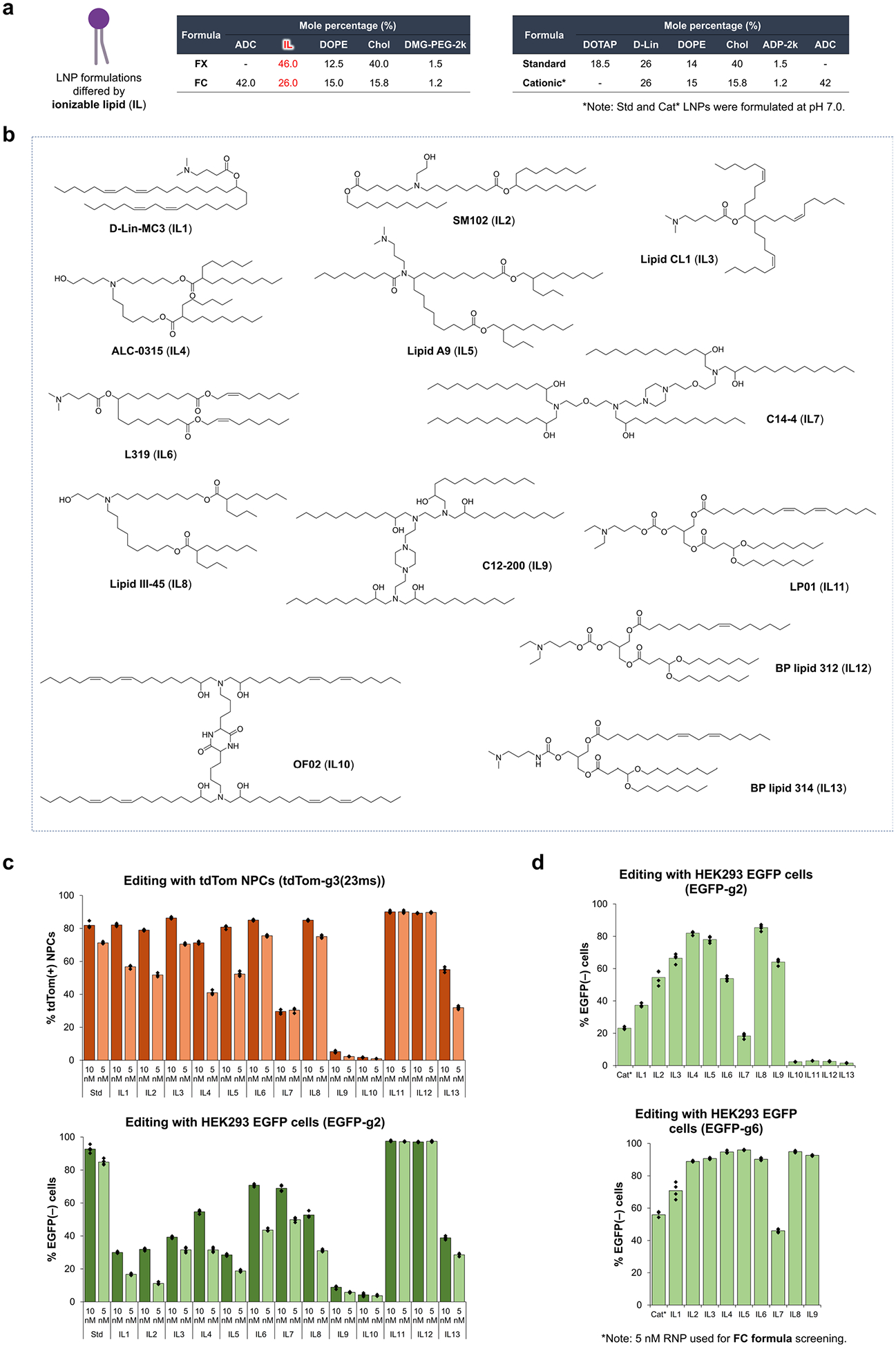
Rescreening of ionizable lipids to improve LNP delivery efficiency of iGeoCas9 RNP. **a**. Lipid compositions for LNP formulations shown in the tables. **b**. Structures of ionizable lipids (IL1 to IL13). **c**. Screening of ionizable lipids for the FX formulation to deliver iGeoCas9 RNP to Ai9 tdTom NPC and HEK293T EGFP cells for genome editing. Genome-editing efficiencies quantified based on tdTom(+) or EGFP(−) signals using iGeoCas9 RNP:LNP complexes in two doses. n = 4 for each group, data are presented as mean values with individual data points. **d**. Screening of ionizable lipids for the FC formulation to deliver iGeoCas9 RNP to HEK293T EGFP cells for EGFP knock-down. Genome-editing efficiencies quantified based on EGFP(−) signal using iGeoCas9 RNP:LNP complexes. n = 4 for each group, data are presented as mean values with individual data points. iGeoCas9 used in this figure is 2NLS-iGeoCas9(C1)-2NLS.

A total of 13 ionizable lipids were evaluated in the FX and FC formulations and were screened for genome editing of tdTomato NPCs and HEK293 EGFP cells, respectively (Extended Data Fig. 6), using a low RNP dose (5 nM) to identify the most efficient LNP formulations. The lipids LP01 (IL11) and BP lipid 312 (IL12) were the most effective ionizable lipids identified from the FX formulation screen (Fig. 5b). LP01 is a well-studied biodegradable ionizable lipid that has a half-life of 6 h in the mouse liver and was previously used for delivering Cas9 mRNA+sgRNA to the liver for genome editing in mice, as demonstrated by Intellia Therapeutics^51^. The lipid BP 312 is a structural analog of LP01. During the screening of FC formulations, ionizable lipids with branched tails, including ALC-0315 (IL4), lipid A9 (IL5) and lipid III-45 (IL8), were found to be more effective for RNP delivery than d-Lin (IL1), SM102 (IL2) and L319 (IL6) with linear tails (Fig. 5b), suggesting that RNPs with defined three-dimensional structures may impose structural requirements on the encapsulating lipids for effective delivery. Thus, two new formulations, FX12 and FC8, composed of BP lipid 312 (IL12) and lipid III-45 (IL8) based on the general FX and FC formulations, respectively, were established.

**Extended Data Fig. 7 |:**
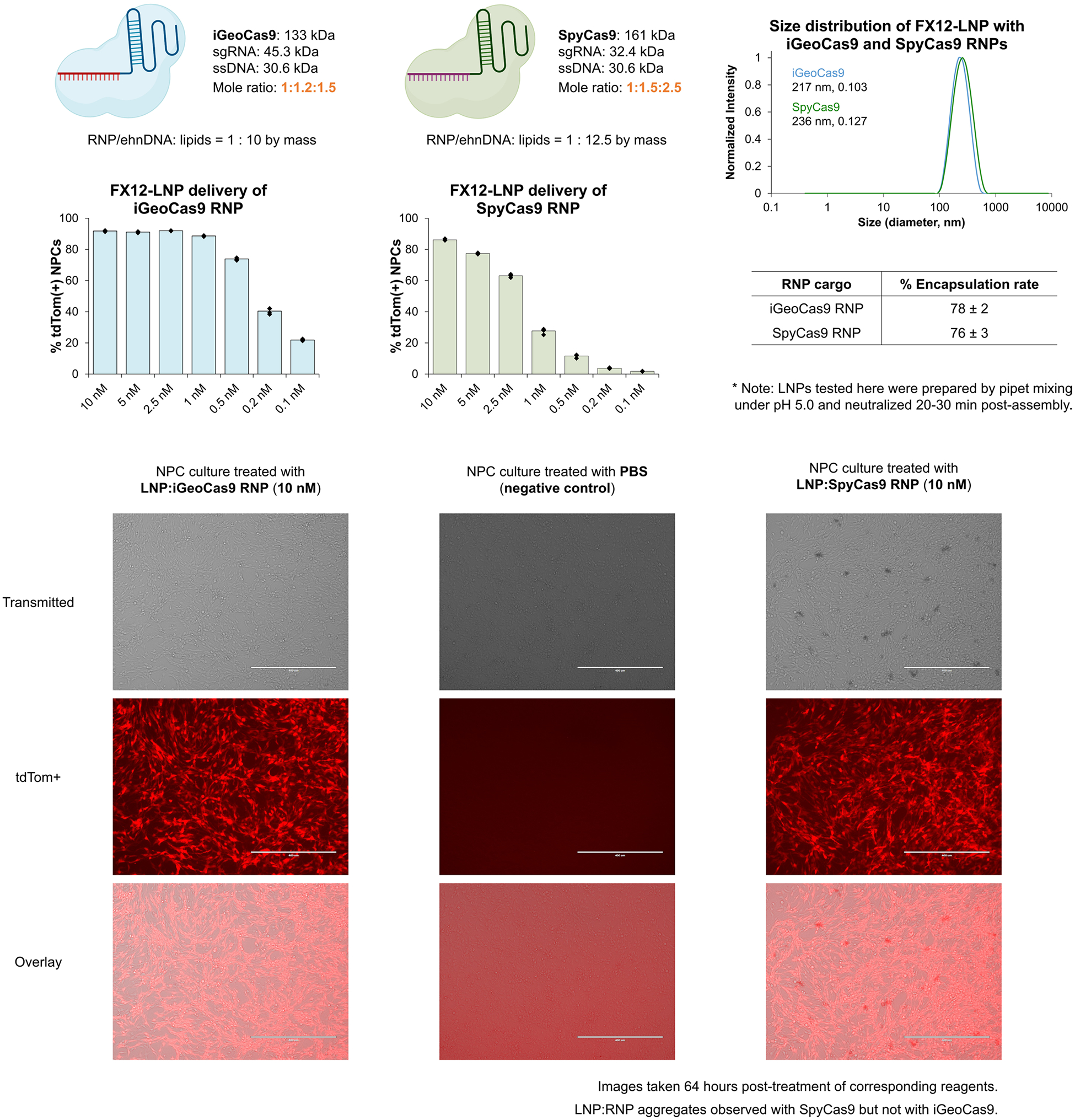
Comparison of the editing efficiency of iGeoCas9 and SpyCas9 in Ai9 tdTom NPCs based on FX12-LNP delivery of corresponding RNP. LNP characterization shows similar encapsulation properties for iGeoCas9 and SpyCas9 RNP cargoes, but SpyCas9 has much lower efficiency compared to iGeoCas9, especially at low RNP dosages. n = 4 for each group, data are presented as mean values with individual data points or ± standard deviation (encapsulation rates). Imaging of NPC cultures suggests that SpyCas9 RNP:LNP complexes tend to form aggregates in the culture media, probably due to the instability of SpyCas9 RNP, while no LNP aggregates were visibly observed for iGeoCas9 RNP. iGeoCas9 used in this figure is 2NLS-iGeoCas9(C1)-2NLS.

Characterization of the FX12 and FC8 LNPs assembled with a microfluidic device demonstrated that both formulations encapsulated RNPs with high efficiency (80% and 98%, respectively) and generated nanoparticles with sizes and polydispersity suitable for in vivo applications (average size: 176 nm and 112 nm, respectively; PDI: 0.10–0.11) (Fig. 5c and Supplementary Fig. 10). In addition, FX12 and FC8 LNPs exhibited minimal cytotoxicity and did not impact cell viability in vitro. More importantly, the FX12 and FC8 formulations enabled RNP delivery and genome editing under conditions of ultralow RNP dosages compared to the previous standard and cationic* formulations (Fig. 5d). For instance, FX12 LNPs showed nearly one-order-of-magnitude-higher genome-editing activity at a 1 nM RNP dose and over two-orders-of-magnitude-higher genome-editing activity at a 100 pM RNP dose compared to the previously established standard formulation. The new FX12 formulation could also deliver SpyCas9 RNP with improved efficiency but still suffers from the issue of RNP instability (Extended Data Fig. 7).

**Extended Data Fig. 8 |:**
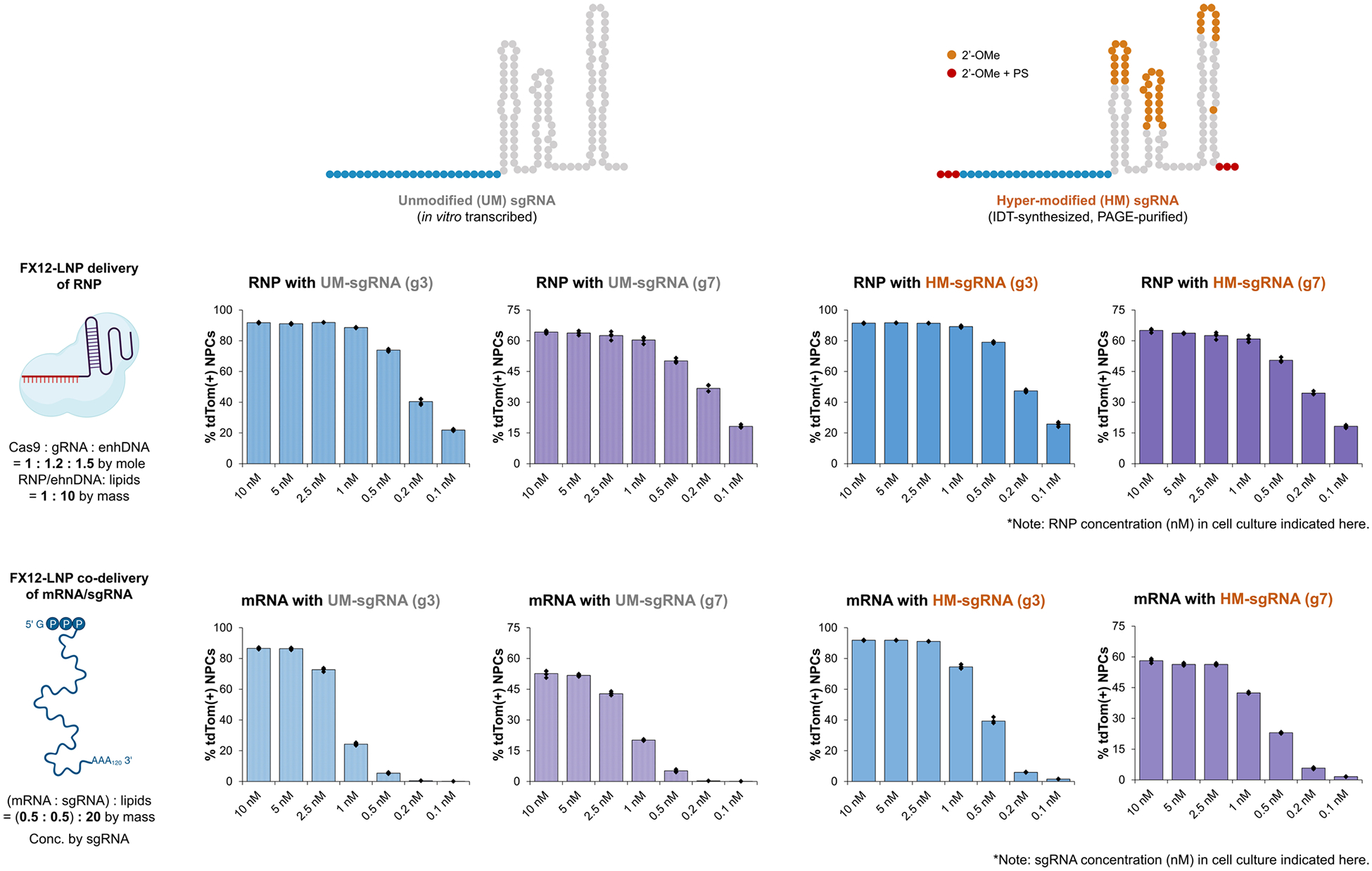
Comparison of the editing efficiency of iGeoCas9 genome editors delivered as mRNA+sgRNA versus RNP using FX12-LNP in Ai9 tdTom NPCs. mRNA delivery is sensitive to sgRNA stability and requires hypermodification of sgRNA to enable successful editing at low mRNA+sgRNA dosage, while sgRNA with modification or not does not affect the editing efficiency based on RNP:LNP delivery. Overall, RNP delivery outperforms mRNA+sgRNA delivery, especially with low cargo dosages. n = 4 for each group, data are presented as mean values with individual data points. iGeoCas9 used in this figure is 2NLS-iGeoCas9(C1)-2NLS.

Because LNP formulations similar to FX12 have been used for in vivo Cas9 mRNA+sgRNA delivery^51,52^, we compared the delivery efficiency of mRNA and RNP by these LNPs (Fig. 5e and Extended Data Fig. 8). mRNA+sgRNA–LNP delivery of CRISPR gene editors requires chemical modifications of the sgRNA to prevent its rapid degradation in the cellular environment. We, therefore, evaluated two sets of sgRNAs, unmodified (UM, by in vitro transcription (IVT)) and hyper-modified (HM, by Integrated DNA Technologies (IDT) synthesis and PAGE purification), for delivery with iGeoCas9 mRNA and RNP. As expected, the use of HM-sgRNA led to a 3-fold to >10-fold improvement in genome-editing activity compared to UM-sgRNA when codelivered with iGeoCas9 mRNA in LNPs. However, there was little difference between UM and HM-sgRNAs when delivered as RNPs, suggesting that the sgRNA is well protected by the Cas9 protein in the RNP format. In addition, RNP delivery showed higher editing efficiency than mRNA+sgRNA delivery, especially at low doses of editing materials, supporting the conclusion that effective RNP delivery can be more advantageous than mRNA delivery by circumventing inefficient translational processes.

### iGeoCas9 RNP–LNP complexes edit organs efficiently in vivo

Having demonstrated that iGeoCas9 RNPs can be delivered by FX12 and FC8 LNPs in vitro with high efficiency, we then asked if this RNP delivery strategy can trigger in vivo genome editing in mice following intravenous injections. More importantly, we wanted to test whether iGeoCas9 RNPs can be delivered to organs beyond the liver, which represents a major challenge for LNP-mediated delivery of CRISPR genome editors and other molecular cargoes.

**Extended Data Fig. 9 |:**
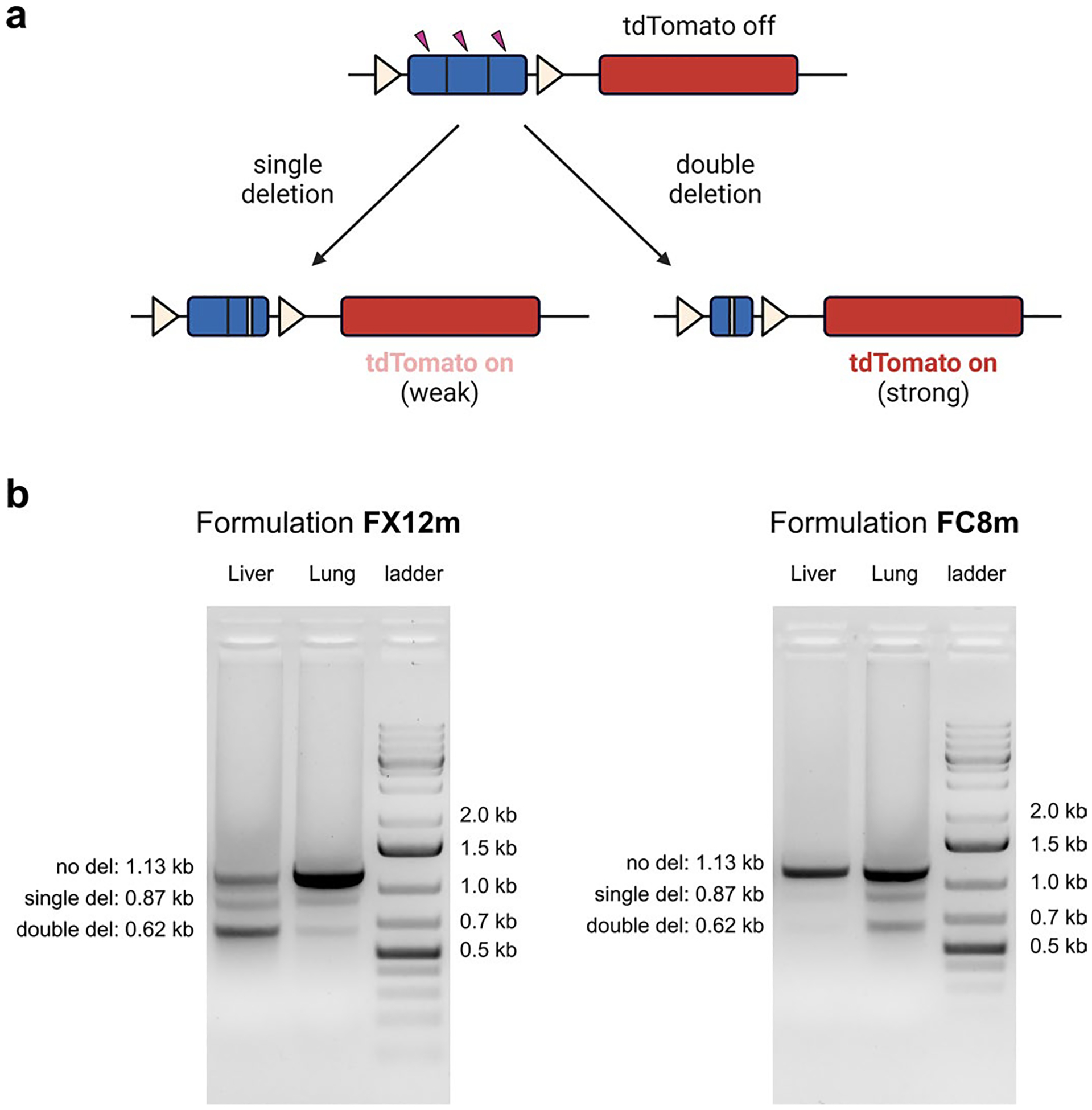
PCR validation of liver and lung editing with Ai9 tdTomato mice. **a**. Schematic illustration of iGeoCas9-mediated transgene editing in Ai9 mouse models to turn on tdTomato expression. **b**. PCR validation of genome edits in the liver and lungs of Ai9 tdTomato mice following IV injections of FX12m and FC8m LNPs.

**Fig. 6 |:**
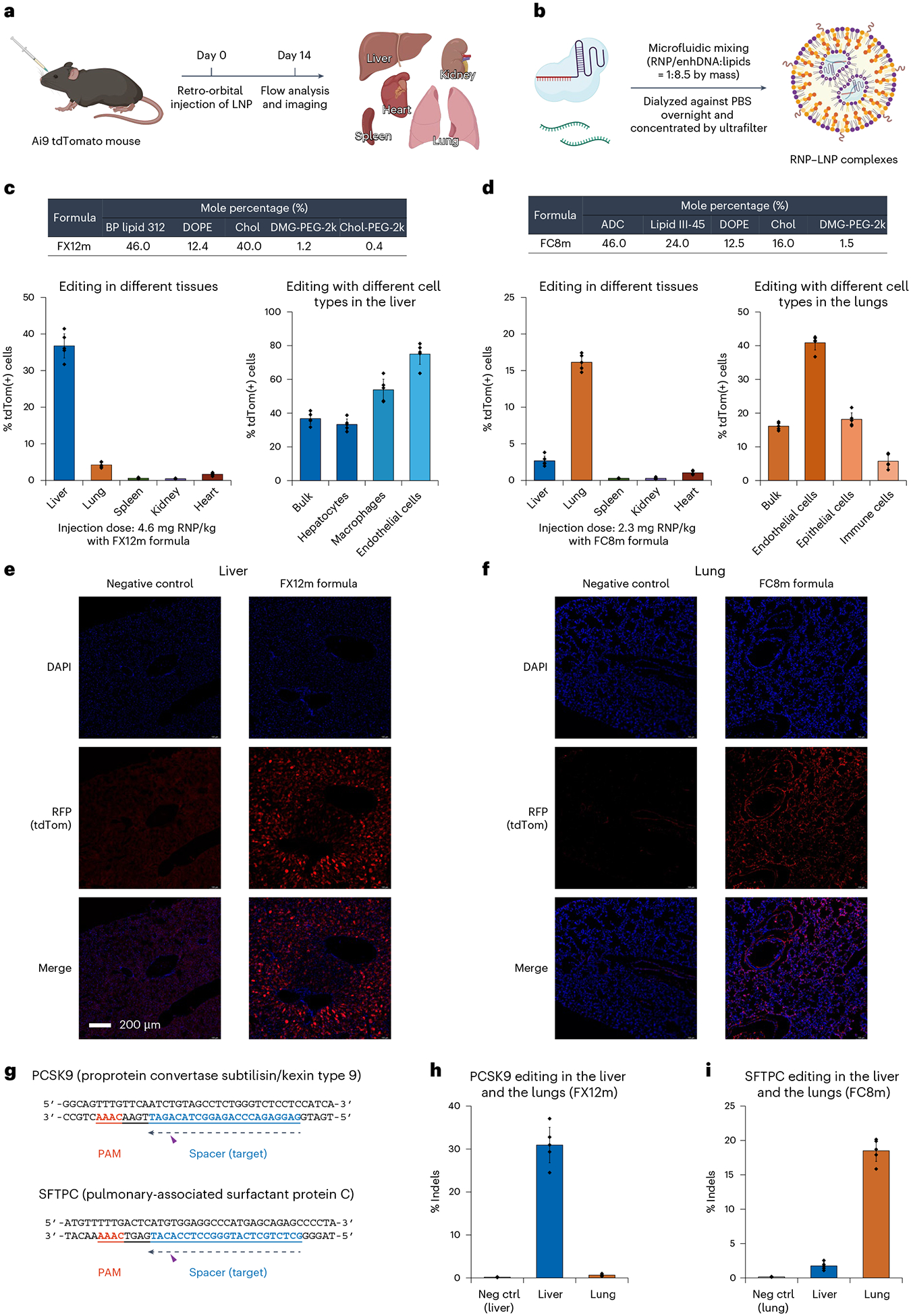
iGeoCas9 RNP–LNPs efficiently edit the liver and lungs of mice. **a**, Schematic diagram of the experimental design used to evaluate iGeoCas9 RNP–LNP-mediated editing in Ai9 mice. **b**, Schematic presentation of LNP preparation procedures. **c**, The modified FX12 LNP formulation (FX12m, with lipid compositions indicated in the table) primarily edits the liver tissue with 37% efficiency. In vivo genome-editing levels in different tissues and different cell types in the liver were quantified by tdTom(+) signals using flow cytometry. **d**, The modified FC8 LNP formulation (FC8m, with lipid compositions indicated in the table) primarily edits the lung tissue with 16% efficiency. In vivo genome-editing levels in different tissues and different cell types in the lungs were quantified by tdTom(+) signals using flow cytometry. For **c** and **d**, *n* = 5 for each group; data are presented as mean values with individual data points and the s.d.; IVT sgRNA, tdTom-g3(23), was used. **e**,**f**, Nuclear staining with DAPI (blue) and imaging of tdTomato (red) in the edited and nonedited liver (**e**) or lung (**f**) tissues. Editing signals were observed with the tissues from experimental mice (*n* = 5). RFP, red fluorescent protein. **g**, sgRNA target designs for *PCSK9* and *SFTPC* gene editing with iGeoCas9 in the liver and lungs, respectively. **h**,**i**, In vivo *PCSK9* and *SFTPC* gene-editing levels (indels) in the liver and lung tissues using FX12m and FC8m LNP formulations, respectively, as quantified by NGS (*n* = 5 for each group); data are presented as mean values with individual data points and the s.d.; PBS-only injections are included as negative controls and the indels in the liver (FX12m) or in the lungs (FC8m) are shown as the blank editing levels. iGeoCas9 used in this figure is 2NLS-iGeoCas9(C1)-2NLS. Neg ctrl, negative control.

We used tdTomato Ai9 mice to assess the delivery and editing efficacy of our LNP-based delivery system for iGeoCas9 RNPs (Fig. 6a). The success of organ-specific mRNA delivery using SORT LNPs^17^ prompted us to test the ability of different lipid formulations to deliver genome editors to organs beyond the liver. Small modifications were made to the FX12 and FC8 LNP formulations to afford FX12m and FC8m formulations for in vivo RNP delivery targeting the liver and the lungs, respectively (Fig. 6b–d). We performed a single retro-orbital injection of LNPs at an RNP-based dose of 4.6 mg kg^−1^ (1.4 mg kg^−1^ based on sgRNA) for FX12m LNPs and 2.3 mg kg^−1^ (0.7 mg kg^−1^ based on sgRNA) for FC8m LNPs. Mice were killed 2 weeks after LNP injection and the organs, including the liver, lung, spleen, heart and kidney, were collected for tdTomato signal analysis to determine genome-editing levels (Fig. 6c,d). Imaging of the organ slices together with flow quantification of tdTomato-positive cells revealed that iGeoCas9 RNPs could be delivered in vivo with LNPs to induce robust genome editing: the FX12m LNP formulation had an average of 37% editing in the liver and the FC8m formulation generated an average of 16% edits in the lungs (*n* = 5; controls were PBS-treated Ai9 mice) (Fig. 6c–f, Extended Data Fig. 9 and Supplementary Figs. 11–13). Specifically, our FX12m LNP formulation drove the delivery of RNPs primarily to the liver, triggering 34%, 54% and 75% editing in the hepatocytes, macrophages and endothelial cells, respectively; the FC8m LNP formulation containing ADC as the biodegradable cationic lipid shifted the delivery specificity to the lungs, generating 41%, 18% and 6% genome editing in endothelial, epithelial and immune cells, respectively. In addition, genome editing was also observed in other tissues that are challenging delivery targets, such as the heart (1–2% genome editing indicated by the tdTomato signal; Supplementary Fig. 13). Notably, no detectable immune response was observed in the experimental mice after LNP injection at the given doses of the FX12m or FC8m formulations, suggesting low cytotoxicity and low immunogenicity of our LNP reagents (Supplementary Fig. 14).

The high efficacy of the FX12m or FC8m formulations in vivo prompted us to explore their potential for genome editing of the disease-causing genes *PCSK9* and *SFTPC* in the liver and lungs, respectively. *PCSK9* was selected as the liver gene target because its editing attenuates hypercholesterolemia. The *SFTPC* gene was selected as the target gene in the lungs because it encodes the surfactant protein C and gain-of-function mutations in the gene, such as I73T, can cause interstitial lung diseases^53^ (Fig. 6g). Following similar experimental procedures, LNPs were assembled and injected into wild-type mice. The liver and lung tissues of edited mice 10 days after injection were collected for NGS analysis to quantify editing levels. The NGS results revealed successful editing of *PCSK9* in the liver (with an average of 31%) (Fig. 6h) and *SFTPC* in the lungs (with an average of 19%) (Fig. 6i) using FX12m and FC8m LNPs, respectively. Overall, these results highlight the potential of our LNP-based RNP delivery system for therapeutic gene editing.

## Discussion

Here, we describe a generalizable platform for CRISPR genome editing both in vitro and in vivo based on LNP-mediated delivery of a thermostable genome editor in the RNP format. Although RNP delivery offers several potential advantages over viral-based or mRNA-based delivery strategies, its use for in vivo genome editing has been limited to tissue editing based on local administration or injection or liver editing through intravenous injection^20^. RNP delivery usually relies on different nanoparticle materials to encapsulate and transport RNPs; however, their applications for in vivo genome editing are commonly restricted by poor particle uniformity, stability and biocompatibility^54^. The use of the thermostable iGeoCas9 genome-editing enzyme described here, along with newly developed LNP formulations, enables robust RNP encapsulation and tissue-selective genome editing in mice. The engineered iGeoCas9 mutants maintain superior stability to the commonly used SpyCas9 under a variety of conditions relevant to in vivo delivery and possess enhanced genome-editing capability because of the tolerance of mutations beneficial to function while preserving molecular structure^55^. Together, LNP-mediated iGeoCas9 RNP delivery may provide a new approach to targeted in vivo genetic treatments.

**Extended Data Fig. 10 |:**
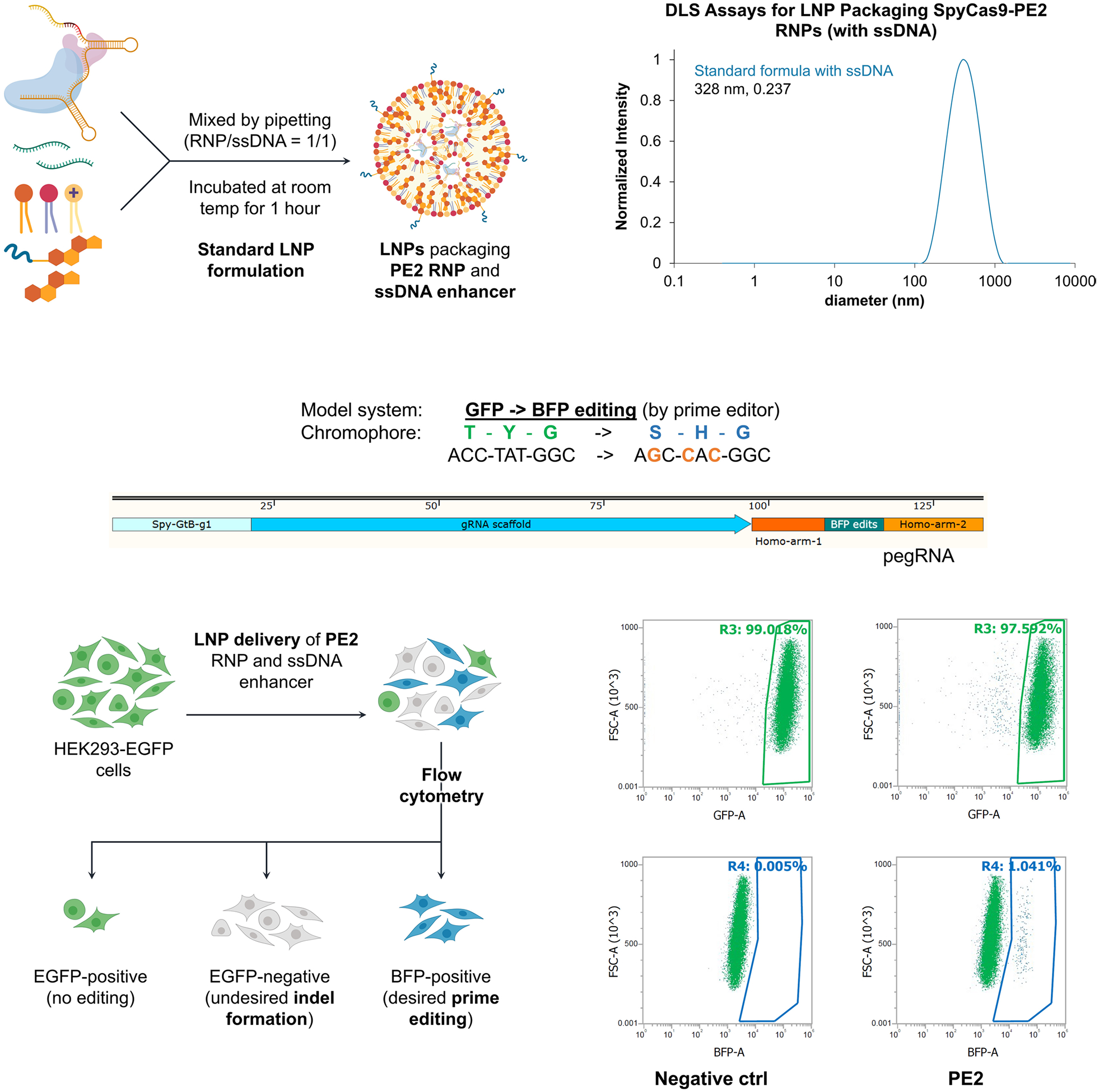
LNP delivery of prime editor RNP. Preliminary results of LNP delivery of prime editor (PE2, based on SpyCas9) showed 1% efficiency in achieving the desired GFP-to-BFP conversion in HEK293T cells. Optimization of the RNP:LNP complex by using a more stable prime-editor RNP, along with an improved LNP formulation, is expected to further enhance the editing efficiency.

LNPs enable nonviral delivery of multiple US Food and Drug Administration-approved RNA therapeutic agents, including the siRNA drug patisiran and the mRNA-based coronavirus disease 2019 vaccines^56^. However, LNPs have rarely been used to deliver proteins or protein–RNA complexes because these molecular cargoes tend to denature under the conditions used for LNP preparation. We hypothesized that proteins with high thermal stability and negative charge density would be efficiently delivered using LNPs because they would encapsulate readily without losing their biochemical functions. iGeoCas9 RNP was selected as a candidate for LNP delivery because of its unique combination of thermostability, negative charge density and genome-editing functionality. Optimized LNP formulations in this study contain biodegradable and acid-degradable lipids that were key to the successful development of vehicles enabling efficient intracellular RNP delivery. The LNP system was also used for the codelivery of RNPs and ssDNA templates to incorporate specific genomic changes by HDR. Consistent with a prior report^30^, ssDNA templates promoted RNP encapsulation into LNPs, presumably through transient binding of ssDNA to RNPs. Successful HDR corrected pathogenic mutations in disease model cell lines, highlighting the potential utility of this LNP-based delivery platform for therapeutic applications. We anticipate that this RNP–LNP strategy may be applicable to other genome-editing tools^57^, including prime editors^58^ (Extended Data Fig. 10) and base editors^59^.

The LNP-assisted RNP delivery described here generated genome edits in vivo in both mouse liver and mouse lungs, depending on the LNP formulation used. The delivery specificity could be regulated by the electrostatic charge properties of the LNPs. In particular, LNPs prepared with the biodegradable cationic lipid ADC targeted the lungs preferentially compared to the liver. This shift in targeting preference is hypothesized to involve differential recruitment of plasma proteins to the LNP surface, which changes the LNP cell tropism by altering the cell surface receptors that they engage with in vivo^60^. LNPs with different lipid compositions or formulated under different conditions (for example, solvent pH, manual versus microfluidic mixing and others) would lead to different surface properties of the nanoparticles and induce tunable delivery specificity. Hints of delivery to more challenging cell types in vivo were also observed, as evidenced by low-level genome editing in the heart, and suggest that it may be possible to screen and optimize new LNP formulations to further expand delivery specificity to more challenging targets. Together, these findings demonstrate the utility of RNP–LNPs for both ex vivo and in vivo genome editing in tissues other than the liver and suggest they have great potential for extending applications of CRISPR–Cas9 genomic therapies.

## Methods

### Ethical statement

The research presented here complies with all relevant ethical regulations. All experiments involving animals were reviewed and approved by the Animal Care and Use Committee (ACUC) at the University of California, Berkeley before commencing the study. Housing, maintenance and experimentation of the mice were carried out with strict adherence to ethical regulations set forth by the ACUC at the University of California, Berkeley.

### Plasmid construction

Plasmids used for the expression of different Cas proteins in this study were built on the basis of a pCold vector. The inserts encoding Cas proteins contain an N-terminal CL7 tag followed by an HRV-3C protease cleavage site and a C-terminal 6xHis-tag following another HRV-3C protease cleavage sequence. The insert for the final NLS-GeoCas9(R1W1)-2NLS protein contains an N-terminal sequence consisting of different tags, His_6_-CL7-MBP (maltose-binding protein) followed by an HRV-3C protease cleavage site. The cloning reactions were carried out in a 50-μl reaction containing 1 ng of template plasmid, 1.25 μl of 10 mM dNTP and 1.25 μl of 10 μM each primer using Phusion high-fidelity DNA polymerase (New England Biolabs (NEB), M0530L). After PCR, the reactions were treated with 1 μl of DpnI (NEB, R0176L) for 1 h at 37 °C before gel purification. The plasmids were ligated by Gibson assembly (NEB master mix, E2611L) of the plasmid backbone and insert sequences. The sequences of all the plasmid constructs were confirmed by full plasmid sequencing (Plasmidsaurus).

### Nucleic acid preparation

All the DNA and RNA oligos used in this study, unless otherwise indicated, were purchased from IDT and passed the quality control standard set by IDT. The HM sgRNAs used in the study were laboratory-purified by PAGE. Some of the sgRNAs and ssDNA HDR templates purchased from IDT possess chemical modifications at the 3′ or 5′ ends (Supplementary Tables 2 and 3). The mRNA encoding 2NLS-iGeoCas9(C1)-2NLS was purchased from TriLink and purified with a silica membrane.

### Lipid material preparation

Commercial lipid materials used in this study were purchased from BroadPharm, Avanti Polar Lipids and Cayman Chemical. Acid-degradable lipids, ADP, ADC, Pep-1k and Pep-2k, were laboratory-synthesized following the procedures in our previous publication^47^.

### IVT of sgRNA

Four sgRNAs (UM-tdTom-g3(23), UM-tdTom-g7(23), UM-gPCSK9 and UM-gSFTPC) used in this study were prepared in milligram scale through IVT using HiScribe T7 high-yield RNA synthesis kit (NEB, E2040S). Following the general protocol provided by the supplier, each IVT reaction (1.2–1.4 ml) uses one RNA synthesis kit together with a dsDNA template (30–50 ug) encoding the sgRNA sequence under a T7 promoter. The IVT reaction mixture was incubated at 37 °C for 10–12 h, then treated with DNAse I (100 units; NEB, M0303S) and incubated for another 3–4 h before being quenched by 2× STOP solution containing formamide, bromophenol blue, xylene cyanol and EDTA. Urea–PAGE was used for sgRNA purification and the gel fraction containing the desired sgRNA was crushed into fine pieces and subjected to RNA extraction at 4 °C overnight using sodium acetate buffer (300 mM, pH 5.0). The extracted sgRNA was concentrated using an Amicon ultracentrifugal filter (10-kDa cutoff) to a total volume of 3–5 ml and the concentrated RNA solution was treated with 10 ml of cold isopropanol to allow the sgRNA to precipitate at −20 °C over 6 h. The sgRNA was pelleted by centrifuge and washed using cold 70% ethanol three times. The isolated sgRNA was further dissolved in 1× rCutsmart buffer (1 ml; NEB, B6004S), subjected to terminal triphosphate removal using calf intestinal alkaline phosphatase (100 units; NEB, M0525S) and incubated at 37 °C for 6 h. The reaction mixture was then diluted with sodium acetate buffer (4 ml, 300 mM, pH 5.0) and subjected to phenol–chloroform (5 ml, saturated, pH 5–6) extraction by vigorous vortexing and centrifugation; the aqueous phase was further washed with chloroform (5 ml) by vigorous vortexing and centrifugation three times. The sgRNA-containing aqueous solution was finally subjected to RNA precipitation and isolation; the pellet was dried in the open air to give purified sgRNA.

Purified IVT sgRNAs were dissolved in an endotoxin-free storage buffer (500 μl; 25 mM NaPi, 150 mM NaCl and 200 mM trehalose at pH 7.50). sgRNAs were reannealed by incubation at 64 °C for 5 min, followed by gradual cooling to room temperature. The sgRNA concentration was Nanodrop-determined (after 10–50× dilution). The final yields of GeoCas9 sgRNA by IVT were as follows: UM-tdTom-g3 and UM-tdTom-g7, 4–5 mg per reaction; UM-gPCSK9 and UM-gSFTPC, 8–12 mg per reaction.

### Directed evolution of GeoCas9

A chloramphenicol-resistant (CAM^+^) bacterial expression plasmid was built to have the insert gene of GeoCas9 together with its corresponding sgRNA that targets the *ccdB* gene in the selection plasmid with a PAM of GAAA (g6). Libraries of GeoCas9 mutants were generated by error-prone PCR to introduce random mutagenesis in three different regions (BH-Rec, RuvC-HNH-WED and WED-PI). The error-prone PCR (with an error rate of 3–5 nucleotide mutations per kilobase) was carried out with Taq DNA polymerase (NEB, M0273S) in a reaction containing 2 μl of 10 mM primers, 1.5 μl of 10 mM MnCl_2_ and 2 ng of template plasmid. The plasmid libraries were generated by ligating the mutated fragments with the remaining part of the plasmid through Gibson assembly. The plasmid libraries (~100 ng DNA after clean-up) were electroporated into 50 μl of electronically competent cells made from *E. coli* strain BW25141(DE3) that contained the selection plasmid encoding the arabinose-inducible *ccdB* toxin gene. After recovery of the electroporated bacteria in 750 μl of sulfur-oxidizing bacteria for 1.5 h at 30 °C, the bacteria culture was concentrated; 1% of the total culture was plated onto a Petri agar dish containing only CAM (as control) and the remaining culture was plated on another Petri agar dish containing both arabinose and CAM. Positive colonies that grew on the plates containing both arabinose and CAM were collected in a pool, retransformed (with ~2 ng of plasmid) and replated (100 μl of transformed culture on both control and selection plates). Plasmids of individual colonies from the replated plate were sequenced to obtain mutational information. Validation of the positive clones in the bacterial assay followed the same procedure.

### Protein expression and purification

All the proteins in this study were expressed in *E. coli* BL21 (DE3) cells (Sigma-Aldrich) cultured in 2× YT medium supplemented with ampicillin. The cultivation was carried out at 37 °C with a shaking speed of 160 r.p.m. after inoculation with an overnight starter culture in LB medium containing ampicillin at a ratio of 1:40. When the optical density at 600 nm of the culture reached 0.8–0.9, the culture was cooled down to 4 °C on ice. The expression of Cas proteins was induced by the addition of IPTG to a final concentration of 0.1 mM and incubated at 15.8–16 °C with a shaking speed of 120 r.p.m. for 14–16 h.

To purify the Cas (or fusion) proteins, the cultured cells were harvested and resuspended in lysis buffer (50 mM Tris-HCl, 20 mM imidazole, 1.2 M NaCl, 10% (v/v) glycerol, 1 mM TCEP and cOmplete protease inhibitor cocktail tablets (Millipore Sigma, 1 tablet per 50 ml) at pH 7.5), disrupted by sonication and centrifuged at 35,000*g* for 45 min. Ni-NTA resin was treated with the supernatant at 4 °C for 60 min, washed with wash buffer 1 (lysis buffer without protease inhibitor cocktail tablet), and eluted with elution buffer (50 mM Tris-HCl, 300 mM imidazole, 1.2 M NaCl, 10% (v/v) glycerol and 1 mM TCEP at pH 7.5) to give crude His-tagged Cas proteins. The nickel elution was then subjected to Im7-6B resin in a slow gravity column repeatedly (3–4 times). The Im7-6B resin was washed with wash buffer 2 (50 mM Tris-HCl, 1.2 M NaCl, 10% (v/v) glycerol and 1 mM TCEP at pH 7.5) before being treated with HRV-3C protease (1% weight to crude Cas protein) for 2–2.5 h to release the Cas proteins from the CL7 and 6xHis-tags. Heparin affinity column was used to further purify the desired proteins. The protein fractions were collected, concentrated and stored in the storage buffer (25 mM NaPi, 150 mM NaCl and 200 mM trehalose at pH 7.50) after buffer exchange. The final yields of different Cas proteins (all with two copies of NLS at both N and C termini) were as follows: wild-type GeoCas9, ~10 mg per 1 L of culture; GeoCas9 mutants, in a range of 2–10 mg per 1 L of culture; SpyCas9, ~4 mg per 1 L of culture; iCas12a, ~30 mg per 1 L of culture; PE2 (nSpyCas9-RT), 1–2 mg per 1 L of culture.

The purification of NLS-GeoCas9(R1W1)-2NLS and 2NLS-GeoCas9(R1-GRK)-2NLS proteins was slightly different after Ni-NTA resin purification. The nickel elution was subjected to dialysis against dialysis buffer (50 mM Tris-HCl, 10 mM imidazole, 1.2 M NaCl, 10% (v/v) glycerol and 1 mM TCEP at pH 7.5) containing HRV-3C protease (1% weight to crude Cas protein) for 12–15 h. The tag-cleaved protein was then loaded to a heparin column and washed with 80 column volumes of buffer containing 0.1% Triton X-114 at 4 °C to minimize endotoxin impurities. The protein fractions were collected, concentrated and subjected to further purification using a size-exclusion column in an endotoxin-free manner. The purified protein was stored in an endotoxin-free storage buffer (25 mM NaPi, 150 mM NaCl and 200 mM trehalose at pH 7.50). The final yields of the desired GeoCas9 mutants were 3–5 mg per 1 L of culture.

### Measurement of protein melting temperatures

Protein melting temperatures were measured using the thermal shift assay (GloMelt, 33021). The assay was performed on a qPCR system with a temperature increase rate of 2 °C min^−1^. The protein melting temperatures were determined as the peak values in the derivative curves of the melting curves.

### Cell lines and culture conditions

NPCs were isolated from embryonic day 13.5 Ai9 tdTomato homozygous mouse brains. Cells were cultured as neurospheres at 37 °C with 5% CO_2_ in NPC medium containing DMEM/F12 (Gibco, 10565018) with GlutaMAX supplement, sodium pyruvate, 10 mM HEPES, nonessential amino acids (Gibco, 11140076), penicillin and streptomycin (Gibco, 10378016), 2-mercaptoethanol (Gibco, 21985023), B-27 without vitamin A (Gibco, 12587010), N2 supplement (Gibco, 17502048) and growth factors bFGF (BioLegand, 579606) and EGF (Gibco, PHG0311) (both 20 ng ml^−1^ as final concentration). NPCs were passaged using the MACS neural dissociation kit (Papain, 130-092-628) following the manufacturer’s protocol. bFGF and EGF were refreshed every 3 days and cells were passaged every 4–5 days. Precoating with a coating solution containing poly(dl-ornithine) hydrobromide (Sigma-Aldrich, P8638), laminin (Sigma-Aldrich, 11243217001) and fibronectin bovine plasma (Sigma-Aldrich, F4759) was required for culturing cells in 96-well plates.

HEK293T and HEK293T EGFP cells were grown in a medium containing DMEM (Gibco, 10569010), high glucose, GlutaMAX supplement, sodium pyruvate, 10% FBS, penicillin and streptomycin (Gibco, 10378016) at 37 °C with 5% CO_2_. Cells were passaged every 3 days.

16HBEge cells were grown in a medium containing MEM (Gibco, 11090099), 10% FBS, penicillin and streptomycin (Gibco, 10378016) at 37 °C with 5% CO_2_. T75 flasks precoated with a coating solution containing LHC-8 basal medium (Gibco, 12677–027), 7.5% BSA (Gibco, 15260–037), bovine collagen solution, type 1 (Advanced BioMatrix, 5005) and fibronectin from human plasma (Thermo Fisher Scientific, 33016–015) were used for culturing 16HBEge cells. Cells were passaged every 4–5 days. Precoating was required for culturing cells in 96-well plates.

### RNP assembly

For cell culture experiments, RNPs were assembled at a 1.2:1 molar ratio of sgRNA (IDT or IVT) to Cas protein in a supplier-recommended buffer (for nucleofection) or a phosphate buffer (25 mM NaPi, 150 mM NaCl and 200 mM trehalose at pH 7.50) immediately before use; it is crucial to slowly add the Cas protein solution to the sgRNA solution while swirling (the reverse addition order can cause RNP aggregate formation). The solution was incubated for 15–25 min at room temperature or 5–10 min at 37 °C. For nucleofection, RNPs were further complexed with Alt-R Cas9 electroporation enhancer (100-nt ssDNA; IDT, 10007805) with a 1:1 molar ratio of enhancer to RNP in supplier-recommended buffers (Lonza). For LNP assembly, RNPs ± ssDNA were further diluted with a neutral solution of PBS and water (1:1, pH 7.3–7.5) or an acidic solution of sodium citrate (10 mM, pH 5.0) to a certain RNP concentration.

### Genome editing with different cell lines

For nucleofection, 250,000 NPCs or 200,000 HEK293T cells were nucleofected with 100 pmol (or other doses if specified) of preassembled RNP (with 100 pmol of enhDNA) with program codes of EH-100 and CM-130, respectively, according to the manufacturer’s instructions. Lonza SF (for HEK293T cells) and P3 (for tdTomato NPCs) buffers were used for the preparation of nucleofection mixtures (with a total volume of 20 μl). Then, 10% of the nucleofected cells were transferred to 96-well plates. The culture medium for NPCs was refreshed after 3 days; HEK293T cells were split with a ratio of 5:1 after 3 days. Cells were harvested for analysis after further incubation at 37 °C for 2 days.

For LNP delivery, 4,000–6,500 cells per well were seeded in 96-well plates 48 h before LNP treatment (HEK293 cells, 4,000–5,000; NPCs, 5,000–6,000; 16HBEge cells, 6,000–6,500). The culture medium was refreshed 24 h after LNP treatment. HEK293T cells were split after two additional days with a ratio of 1:1 to 2:1 based on cell confluency. Cells were harvested for analysis after a total incubation time of 4–5 days (upon signal maturation for tdTom NPCs and HEK293 EGFP cells).

Cell viability was determined on the basis of the counts of live cells (stained with trypan blue) at certain times after treatment with LNPs in comparison to cells treated with PBS (negative control).

### Flow cytometry

Cell fluorescence was assayed on an Attune NxT acoustic focusing cytometer (Thermo Fisher Scientific) equipped with a 554-nm excitation laser and 585/16 emission filter (tdTomato), 488-nm excitation laser and 530/30 emission filter (EGFP) and 400-nm excitation laser and 440/50 emission filter (BFP) and corresponding setup for cell type analysis based on the antibody fluorophores. Flow data were analyzed using Attune Cytometric Software (version 5.1.1), FlowJo (version 10.7.1), Excel (version 2408) and Prism 9 (version 9.4.1).

### LNP assembly and delivery experiment setup

LNP solutions with a total volume of less than 200 μl were prepared by pipet mixing; LNP solutions with higher volumes were prepared using a microfluidic mixing device, NanoGenerator Flex-M (PreciGenome).

For the preparation of standard and cationic LNPs at small scales, RNPs were assembled by mixing iGeoCas9 and sgRNAs at a molar ratio of 1:1.2 and incubated for 20–30 min at room temperature. For HDR experiments, the assembled RNPs were further mixed with ssDNA templates at a molar ratio of 1:1 (ssDNA to RNP). The RNP stock solutions were diluted with an aqueous solution (PBS and water, 1:1, with 5 mM DTT, pH 7.3–7.5) to give a final RNP concentration of 5.0 or 7.5 μM. The lipid stock solutions in ethanol and DMSO were prepared at a total lipid concentration of 10–12 mg ml^−1^. LNPs were assembled by pipet mixing with a volume ratio of 4:1 (aqueous to organic) and incubated at room temperature for 1 h before being diluted with PBS (3× volume of the LNP solution) to give an LNP stock solution with RNP concentrations of 1.0 or 1.5 μM. For cell culture experiments, the LNP solutions were diluted with the corresponding culture medium (9× volume of the LNP solution in PBS) and then used to treat cultured cells (in 1:1 volume ratio) with a final RNP concentration of 50 or 75 nM RNP (for example, 5.0 or 7.5 pmol of RNP in 100 μl of culture medium).

For long-term storage of standard and cationic LNPs at 4 °C, DTT was excluded during LNP assembly. A solution of DTT (5 mM) in PBS was used to activate LNPs right before the in vitro delivery experiments.

For the preparation of FX12 and FC8 LNPs at small scales, RNPs were assembled by mixing iGeoCas9 and sgRNAs at a molar ratio of 1:1.2, incubated for 10 min at 37 °C, then complexed with 100-nt enhDNA (1.5 equivalents to RNP), and incubated for another 10 min at 37 °C, giving a stock solution of RNP with a concentration of 12–15 μM in the storage phosphate–trehalose buffer. The RNP stock solution was then diluted with sodium citrate buffer (10 mM, pH 5.0) to give a final RNP concentration of 1.06 μM (final pH of ~5.2). The lipid stock solutions in ethanol and DMSO were prepared at a total lipid concentration of 10 mg ml^−1^. LNPs were assembled by pipet mixing with a volume ratio of 4:1 (aqueous to organic) and incubated at room temperature for 20–30 min before being neutralized and diluted by PBS (3.24× volume of the LNP solution) to give an LNP stock solution with an RNP concentration of 0.2 μM. The LNP stock solution could be further diluted to give certain doses used for in vitro RNP delivery experiments. FC8 LNPs were DTT-activated during PBS dilution. For cell culture experiments, the LNP solutions were diluted with the corresponding culture medium (9× volume of the LNP solution in PBS) and then used to treat cultured cells. For instance, 5 μl of the LNP stock solution (with an RNP concentration of 0.2 μM) was diluted with 45 μl of culture medium and used to treat mammalian cells in 50 μl of culture medium in a 96-well plate, giving a final RNP concentration of 10 nM (1.0 pmol of RNP in 100 μl of culture medium).

For the microfluidic preparation of LNPs, RNPs were assembled by mixing iGeoCas9 and sgRNAs with a molar ratio of 1:1.2, incubated for 10 min at 37 °C, then complexed with 100-nt enhDNA (1.5 equivalents to RNP), and then incubated for another 10 min at 37 °C, giving a stock solution of RNP with a concentration of 12–15 μM in the storage phosphate–trehalose buffer. The RNP stock solution was then diluted with sodium citrate buffer (10 mM, pH 5.0) to give a final RNP concentration of 1.25 μM (final pH of ~5.2) or 0.625 μM (final pH of ~5.1). The lipid stock solutions in ethanol and DMSO were prepared with a total lipid concentration of 10 mg ml^−1^ (for FX12m formulation) and 5 mg ml^−1^ (for FX8m formulation). FX12m LNPs were microfluidic-assembled with a volume ratio of 4:1 (aqueous to organic) at a flow rate of 3 ml min^−1^; FC8m LNPs were microfluidic-assembled with a volume ratio of 4:1 (aqueous to organic) at a flow rate of 2 ml min^−1^. The assembled LNPs were incubated at room temperature for 20–30 min before being subjected to dialysis against PBS using a dialysis membrane with an MW cutoff of 10 kDa (Thermo Fisher Scientific) overnight at 4 °C. Upon dialysis, the LNPs were concentrated by ultrafiltration using Amicon ultracentrifugal filter with an MW cutoff of 100 kDa (Millipore). FC8m LNPs were DTT-activated during the concentration step. The filter was washed three times with PBS to collect the remaining LNPs absorbed on the filter membrane. The combined LNP solution was diluted with PBS to a certain volume used for animal experiments.

### Dynamic light scattering (DLS) assay

The size distribution of RNP particles or LNPs was measured using Zetasizer (version 7.13, Malvern Panalytical; He–Ne Laser, *λ* = 632 nm; detection angle = 173°).

### RNP encapsulation rate measurement

Quant-iT RiboGreen RNA reagent (R11491) was used to estimate RNP encapsulation efficiency. LNP and lysed LNP (using 1% Triton X-100) samples were diluted using TE buffer to an estimated total nucleic acid concentration of 0.5–2.0 ng μl^−1^. The diluted (lysed) LNP samples were mixed with the RiboGreen reagent (1:1,000 dilution in TE buffer) in a volume ratio of 1:1 (100 μl + 100 μl) and incubated at room temperature in the dark for 2 min before fluorescent signal measurement with the emission wavelength of 500/525 nm. The unencapsulated RNP proportion was estimated as the ratio of the signal intensity (with blank signal subtracted) of intact LNP to lysed LNP samples, thus giving the corresponding RNP encapsulation rate.

### Cryo-transmission electron microscopy (cryo-TEM) image acquisition and processing

For cryo-TEM imaging, 3 μl of LNP suspension was added to a glow-discharged R2/2 Quantifoil Cu Grid (Ted Pella). Samples were incubated for 20 s and blotted for 4 s (blotting force = 5) in a 4 °C high-humidity chamber. After incubation, the samples were immediately plunge-frozen using an FEI Mark IV Vitrobot (FEI), resulting in vitreous ice. The samples were then imaged with an FEI Talos Arctica at 200 kV under low-dose conditions using a bottom-mounted K3 camera (Gatan) at ×36,000 magnification (0.5705 Å per pixel). Images were analyzed with Cryo-SPARC software (version 4.5.3) and representative images were selected from multiple viewfields and grids.

### In vivo genome editing

Retro-orbital injections of LNPs consisting of different lipid formulations were performed with Ai9 tdTomato mice (C56BL/6J, Jackson Laboratory) and wild-type mice (BALB/c, Jackson Laboratory), 10–16 weeks old, weighing 18–20 g (male or female). The mice were killed and all tissues were collected for further analysis 2 weeks (Ai9 mice) or 10 days (wild-type mice) after LNP injection.

For flow analysis, isolated tissues were minced using a sterile blade and then subjected to digestion with collagenase type I (0.1 mg ml^−1^ as the final concentration; Gibco, 17018029) in 1 ml of Hanks’ balanced salt solution buffer (Gibco, 14175095) supplemented with 5 mM Ca^2+^ at 37 °C for 2 h with gentle shaking. Next, the digested solution was filtered using a 70-μm filter and quenched with PBS containing 2% FBS. A cell pellet was obtained by centrifuging for 5 min at a speed of 1,500*g* at 4 °C. The supernatant was removed and the cell pellet was resuspended in 1 ml of PBS containing 2% FBS, which could be used for flow analysis.

For cell type analysis, the dissociated tissue cells (100 μl) were incubated with corresponding antibodies (1:200 dilution) for 1 h in the dark at 4 °C. The stained cells were washed three times with 500 μl of PBS and then resuspended in 500 μl of PBS for flow cytometry analysis. The antibodies used for liver cell types were Alexa Fluor 647 anti-mouse CD95 (Fas) (BioLegend, 152620, for hepatocytes), Alexa Fluor 647 anti-mouse F4/80 (BioLegend, 157314, for macrophages) and Alexa Fluor 488 anti-mouse CD31 (BioLegend, 102414, for endothelial cells); the antibodies used for lung cell types were Alexa Fluor 647 anti-mouse CD326 (Ep-CAM) (BioLegend, 118212, for epithelial cells), Alexa Fluor 488 anti-mouse CD31 (BioLegend, 102414, for endothelial cells) and Pacific blue anti-mouse CD45 (BioLegend, 157212, for immune cells).

For analysis by imaging, tissue blocks were embedded into optimal cutting temperature compounds (Sakura Finetek) and cosectioned (8 μm) on a Cryostat instrument (Leica Biosystems) to prepare tissue sections. The mounted tissue slices were stained with DAPI before microscopy imaging. Images of tissue slices were taken using the Leica DMi8 microscope and analyzed using the Leica Application Suite X program (version 3.9.1.28433).

For analysis by NGS or DNA gel assays, dissociated tissue cells were collected and treated with Quick Extraction solution (Epicentre) to lyse the cells (65 °C for 20 min and then 95 °C for 20 min). The cell lysates were directly used for gene amplicon prep by PCR.

### NGS

Edited cells were harvested and treated with Quick Extraction solution (Epicentre) to lyse the cells (65 °C for 20 min and then 95 °C for 20 min). The cell lysates were directly used for gene amplicon prep by PCR. Amplicons of genomic targets were PCR-amplified in the presence of corresponding primers, which were designed to have no overlap with their corresponding donor ssDNA sequence in the case of HDR.

The PCR products were purified with magnetic beads (Berkeley Sequencing Core Facility) before being subjected to NGS with MiSeq (Illumina) at 2 × 300 bp with a depth of at least 20,000 reads per sample. The sequencing reads were subjected to CRISPResso2 (https://github.com/pinellolab/CRISPResso2) to quantify the levels of indels and HDR. Subsequent data analysis and presentation were performed with Excel (version 2408) and Prism 9 (version 9.4.1).

### Immunogenicity assessment

Wild-type BALB/c mice (male or female), 10–16 weeks old, weighing 18–20 g, were used for cytokine measurement experiments. For LNP complexes based on FX12m or FC8m formulations with RNP cargo or as empty vectors, RNP-only solution, PBS (negative control) and LPS (lipopolysaccharide, with a dose of 1 mg kg^−1^; positive control) were administered by the retro-orbital route (intravenous). LNPs (with or without RNPs) were injected at doses following the experimental setup (for example, 4.6 mg kg^−1^ RNP for FX12m formulation). Injections were performed with a total volume of 150 μl per mouse.

At time points of 6 and 24 h, the first two batches of mice were killed and corresponding blood samples were collected in heparin and centrifuged at 1,500*g* for 10 min at 4 °C. The levels of cytokines, including interleukin 2 (ELISA kit from R&D Systems, DY402–05), interleukin 6 (ELISA kit from R&D Systems, DY406–05), macrophage inflammatory protein 2 (ELISA kit from R&D Systems, DY452–05) and tumor necrosis factor (ELISA kit from R&D Systems, DY410–05), in the plasma were determined on the basis of ELISA assays following the manufacturer’s protocols (R&D Systems).

Another batch of mice were killed 2 weeks after injection and blood samples were collected in heparin and centrifuged at 1,500*g* for 10 min at 4 °C. The levels of liver damage enzymes, including alanine aminotransferase (ELISA kit from Abcam, ab282882), aspartate aminotransferase (ELISA kit from Abcam, ab263882) and transglutaminase 2 (ELISA kit from RayBiotech, ELM-TGM2–1), in the plasma were determined on the basis of ELISA assays following manufacturers’ protocols (Abcam or RayBiotech).

## Extended Data

## Supplementary Material

SI

## Data Availability

Protein, DNA and RNA sequences in this study are available in the Supplementary Information. Sequences, sequencing data and raw images are available through Dryad (https://doi.org/10.5061/dryad.rr4xgxdfh)^61^. NGS data are available from the National Center for Biotechnology Information (PRJNA1157587). Relevant materials (for example, plasmids and proteins) are available from the corresponding author upon reasonable request or from Addgene.
