# Publisher Correction: Lung and liver editing by lipid nanoparticle delivery of a stable CRISPR–Cas9 ribonucleoprotein

**DOI:** 10.1038/s41587-026-03221-1

**Published:** 2026-06-18

**Authors:** Kai Chen, Hesong Han, Sheng Zhao, Bryant Xu, Boyan Yin, Atip Lawanprasert, Marena Trinidad, Benjamin W. Burgstone, Niren Murthy, Jennifer A. Doudna

**Affiliations:** 1 Department of Molecular and Cell Biology, University of California Berkeley, Berkeley, CA USA; 2 Innovative Genomics Institute, University of California Berkeley, Berkeley, CA USA; 3 Department of Bioengineering, University of California Berkeley, Berkeley, CA USA; 4 Howard Hughes Medical Institute, University of California Berkeley, Berkeley, CA USA; 5 Gladstone Institutes, San Francisco, CA USA; 6 Gladstone-UCSF Institute of Genomic Immunology, San Francisco, CA USA; 7 Molecular Biophysics and Integrated Bioimaging Division, Lawrence Berkeley National Laboratory, Berkeley, CA USA; 8 Department of Chemistry, University of California Berkeley, Berkeley, CA USA; 9 California Institute for Quantitative Biosciences, University of California Berkeley, Berkeley, CA USA

**Keywords:** Protein delivery, Gene therapy, Molecular engineering

Correction to: *Nature Biotechnology* 10.1038/s41587-024-02437-3, published online 16 October 2024.

In the version of the article initially published, in the “Directed evolution of GeoCas9” section of the Methods, the text “After recovery of the electroporated bacteria in 750 µl of sulfur-oxidizing bacteria for 1.5 h at 30 °C” should have read “After recovery of the electroporated bacteria in 750 µl of SOB medium (Super Optimal Broth) for 1.5 h at 30 °C.” This correction has been made to the HTML and PDF versions of the article.

