# Supplementary material for: Lung and liver editing by lipid nanoparticle delivery of a stable CRISPR–Cas9 ribonucleoprotein
Source: Nat Biotechnol. Author manuscript; Available in PMC 2025 Sep 17. (PMC12000389; doi:10.1038/s41587-024-02437-3)
Supplement: Supplementary file 1 [file NIHMS2042429-supplement-SI.pdf]

nature biotechnology

Supplementary information https://doi.org/10.1038/s41587-024-02437-3

Lung and liver editing by lipid nanoparticle delivery of a stable CRISPR–Cas9 ribonucleoprotein

In the format provided by the authors and unedited

Table of Content

Content	Pages
Supplementary Figures 1–14	2–15
Protein sequences	16–22
Supplementary Tables 1–3	23–31

Figure S1. RNP stability comparison of WT-GeoCas9, GeoCas9(R1W1), and SpyCas9 using DLS assay. Incubation at 37 °C can gradually lead to SpyCas9 RNP aggregation but does affect WT-GeoCas9 or GeoCas9(R1W1) within 6 hours.

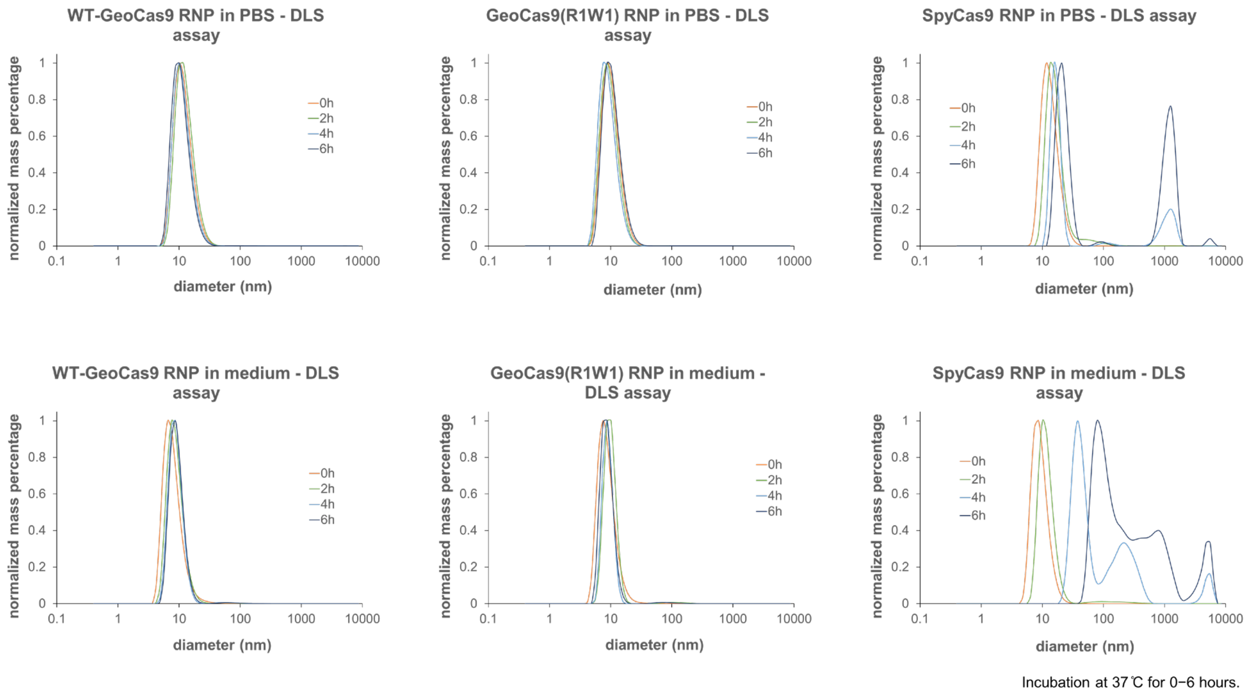

Figure S2. Comparison of iGeoCas9 and SpyCas9 for their genome editing efficiency and RNP stability. **a.** Gene-editing activity comparison of the two editors using the Ai9 tdTom NPC assay with 10 different sgRNA, respectively (tdTom-g1 to g10 for iGeoCas9, and Spy-tdTom-g1 to g10 for SpyCas9, as listed in Table **S2**). SpyCas9 and iGeoCas9(C2) showed overall comparable editing level across the 10 targets. NPCs (0.25 M cells in 20 uL buffer) were nucleofected 25 pmol RNP. Editing efficiencies quantified based on the tdTom(+) signal. n = 4 for each group. Data are presented in box plots where the lower bound of the lower whisker shows the minimum, the lower bound of the box shows the lower quartile, the center of the box shows the median, the upper bound of the box shows the upper quartile and the upper bound of the upper whisker shows the maximum. **b.** Gene-editing activity comparison of the two editors after incubation at 37 °C for certain time using the HEK293T EGFP assay. Incubation in PBS at 37 °C can gradually lead to function loss for SpyCas9 RNP but does affect iGeoCas9(C2) within 12 hours. Editing efficiencies quantified based on the EGFP(–) signal. EGFP-g6 was used for iGeoCas9(C2). n = 4 for each group, data are presented as mean values with individual data points.

a

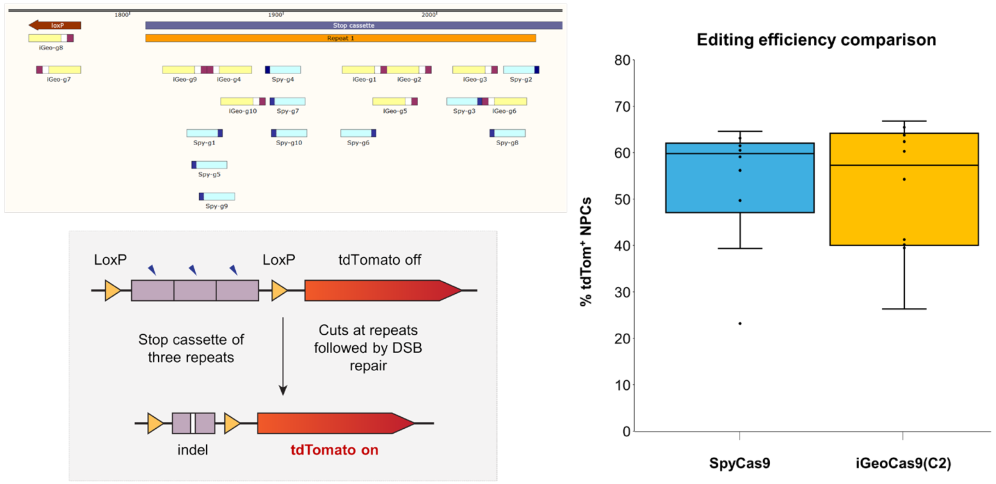

b

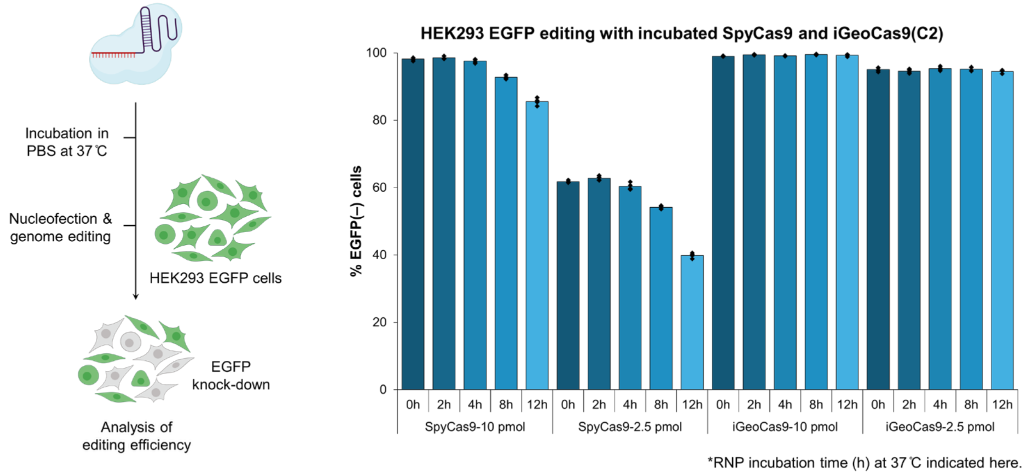

Figure S3. Examples of flow quantification to determine the genome-editing activities by different GeoCas9 RNPs in tdTom NPCs. FSC-A/SSC-A used to define live cells, FSC-A/FSC-H used to define cell singlets, and FSC-A/RFP-A used to determine tdTom(+) cells.

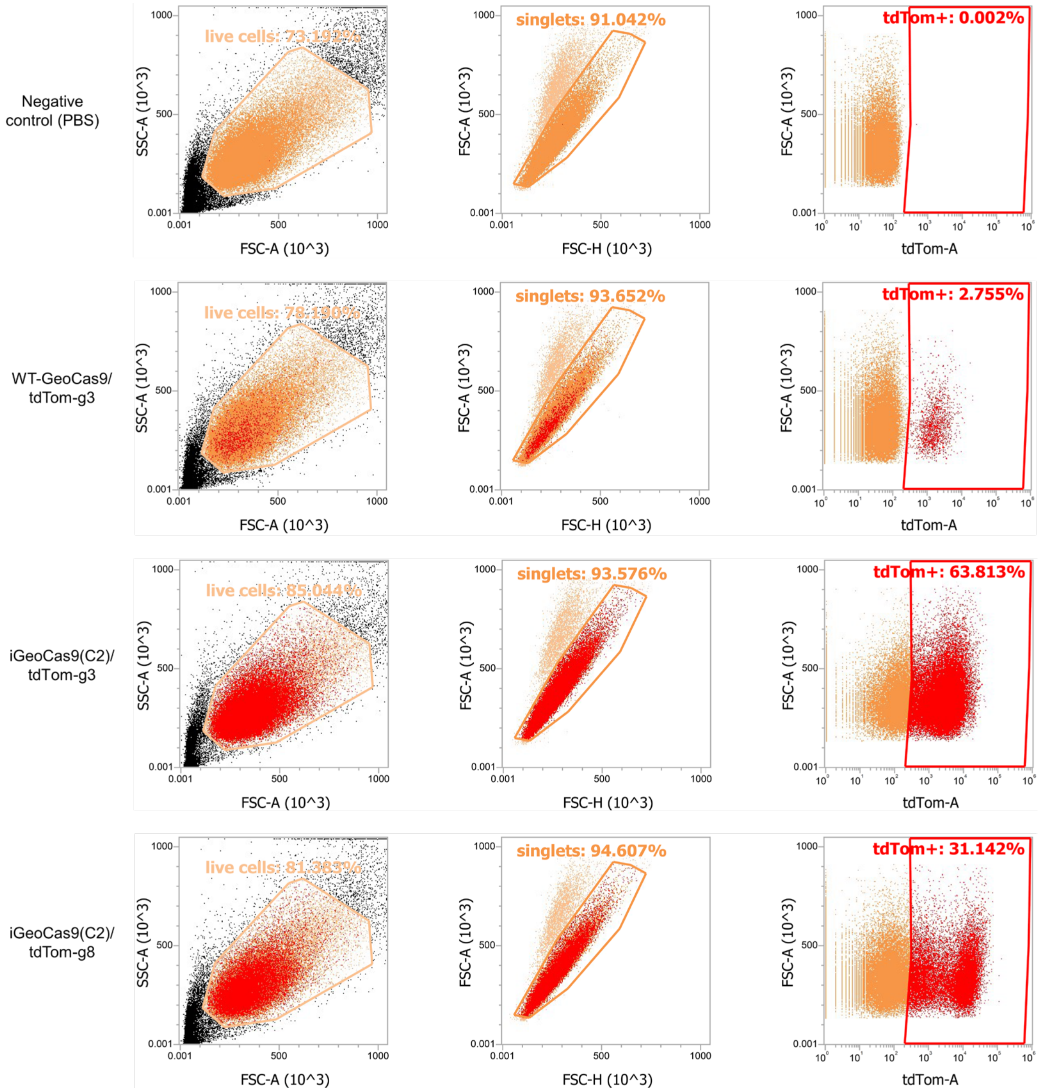

Figure S4. Comparison of WT-GeoCas9 and GeoCas9(R1W1) for their genome editing activities in HEK293T cells to knock down EGFP using different spacer and PAM sequences. GeoCas9(R1W1) shows substantially improved EGFP knock-down efficiency. n = 4 for each group, data are presented as mean values with individual data points.

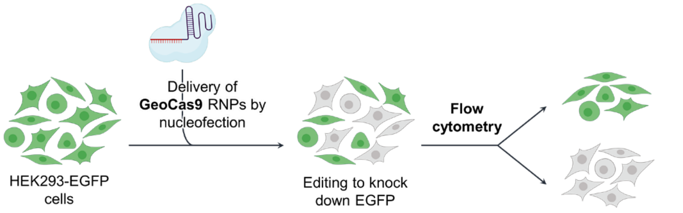

Guides	Spacer	PAM
**EGFP-g1**	ACGACTTCTTCAAGTCCGCCA	TGCC**CGAA**
**EGFP-g2**	ACCTGAGCACCCAGTCCAAGC	TGAG**CAAA**
**EGFP-g3**	CAAGCTGGAGTACAACTACAA	CAGC**CACA**
**EGFP-g4**	GGGCACGGGCAGCTTGCCGGT	GGTG**CAGA**
**EGFP-g5**	TTGAAGAAGATGGTGCGCTCC	TGGA**CGTA**
**EGFP-g6**	ATGCGGTTCACCAGGGTGTCG	CCCT**CGAA**

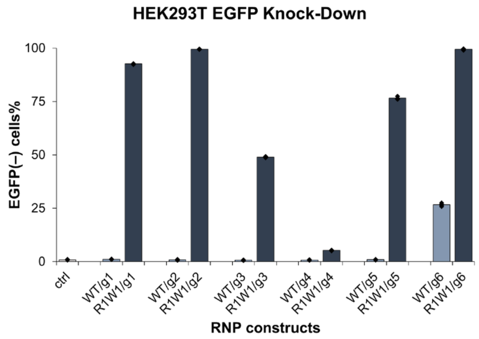

Figure S5. Effect of volume ratio (aqueous/organic) and salt concentration on the packaging efficiency of GeoCas9 RNP in LNP. n = 4 for each group, data are presented as mean values with individual data points.

iGeoCas9(C2)/g3 used as the RNP for optimization

Formula	Mole percentage (%)				
	DOTAP	D-Lin	DOPE	Chol	ADP-2k
Fa	18.3	26.0	13.9	40.3	1.5

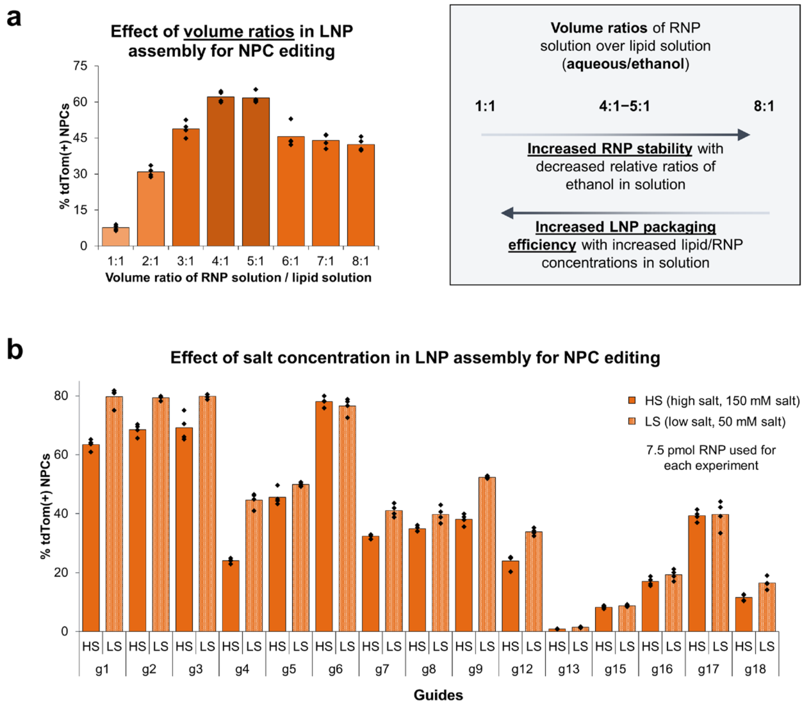

Figure S6. Schematic of the whole procedure for LNP-based RNP delivery in cell culture.

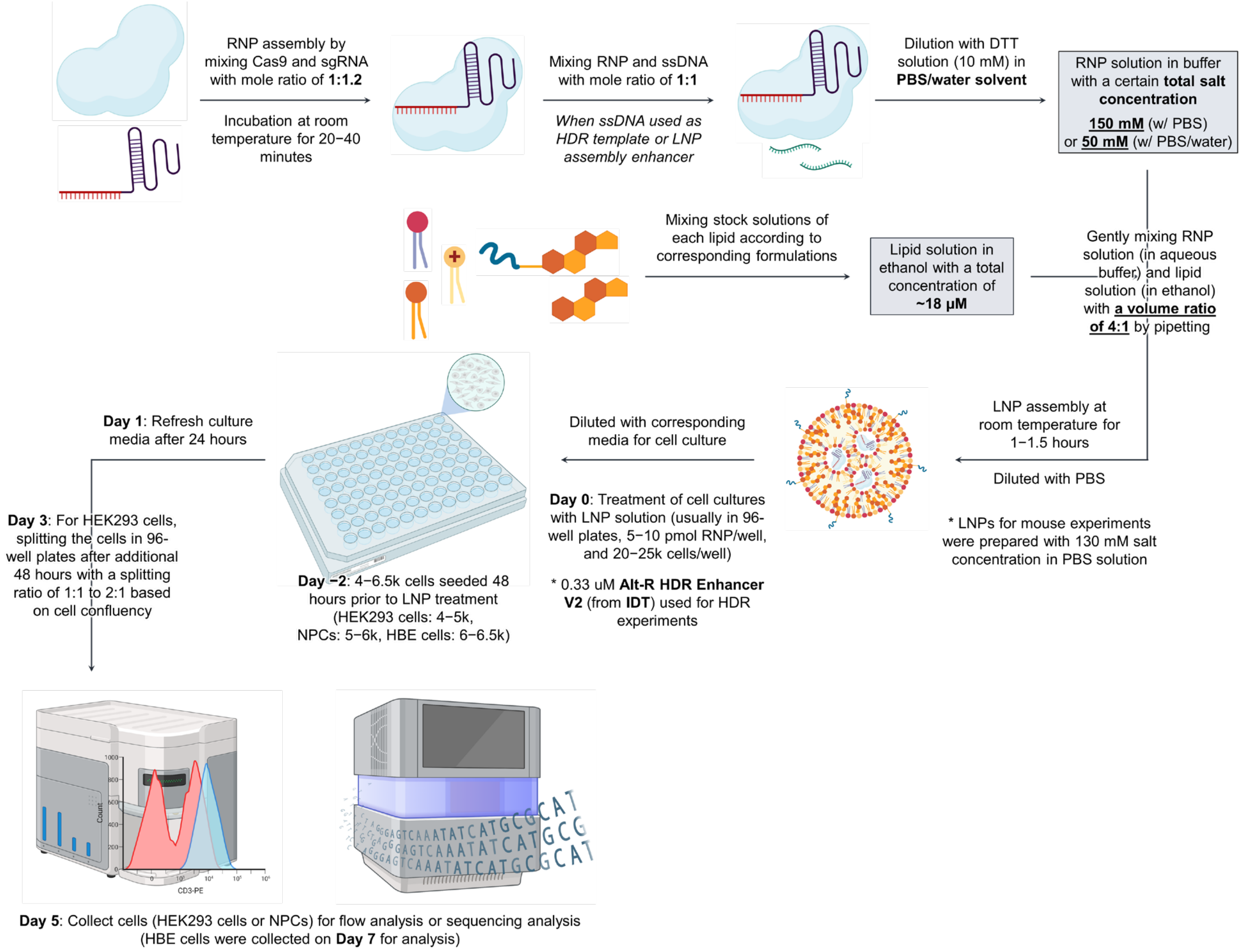

Figure S7. Co-delivery of GeoCas9 RNPs and ssDNA HDR templates to edit the chromophore of EGFP to BFP in HEK293T cells.

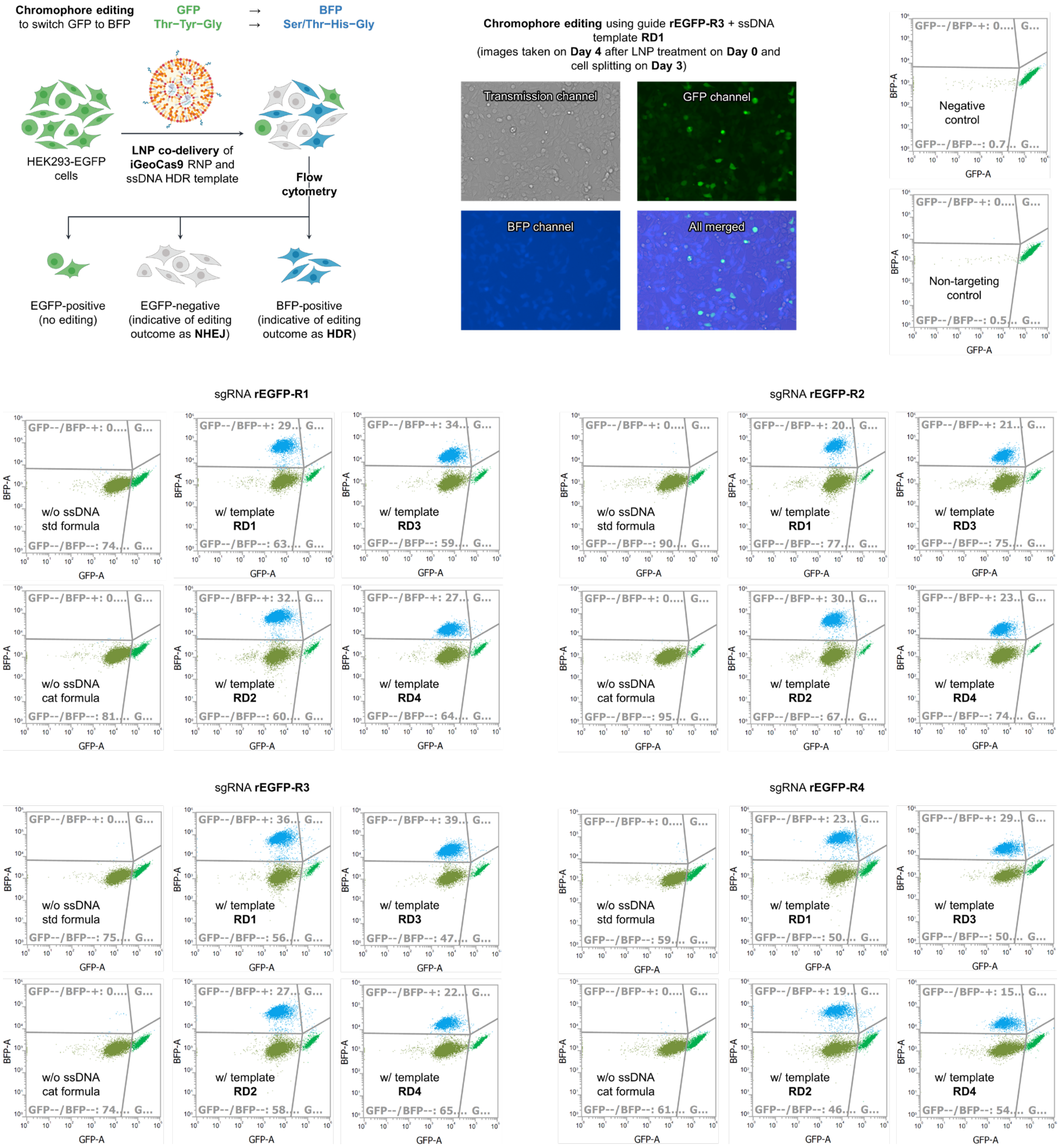

Figure S8. Effect of different anionic polymer additives on the packaging efficiency of RNPs in LNPs. n = 4 for each group, data are presented as mean values with individual data points.

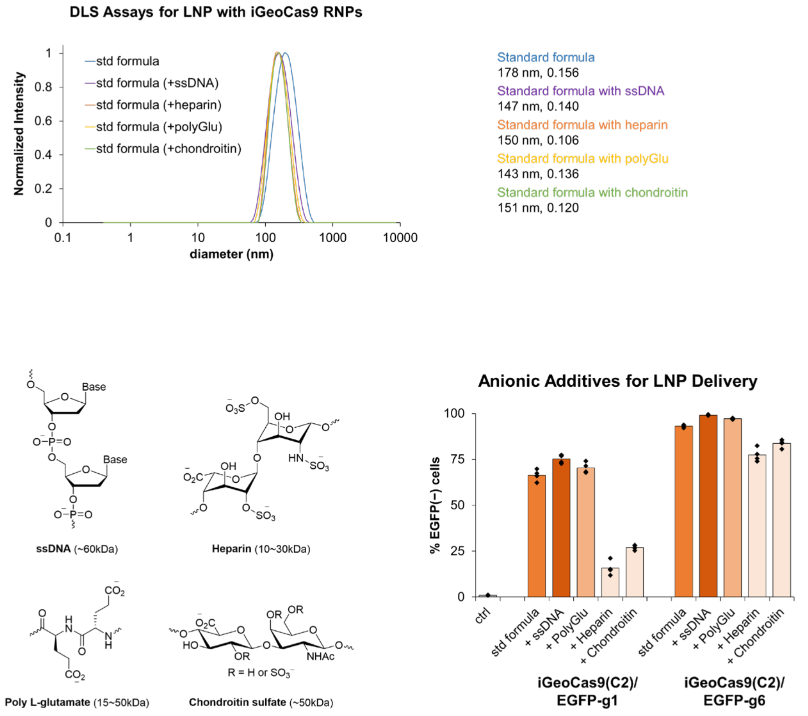

Figure S9. The effect of cationic lipid, DOTAP, on RNP encapsulation rate under neutral pH. n = 4 for each group, data of encapsulation rate are presented as mean values ± standard deviation.

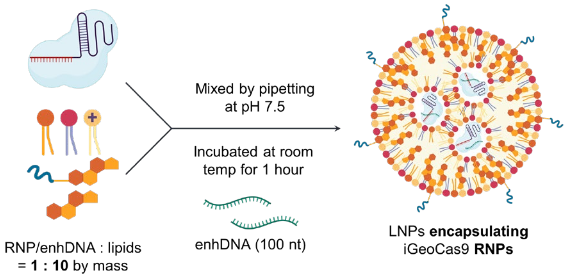

Formula	Mole percentage (%)				
	DOTAP	D-Lin	DOPE	Chol	ADP-2k
**Std**	18.5	26	14	40	1.5
**DOTAP-10**	10	36	12.5	40	1.5
**DOTAP-5**	5	41	12.5	40	1.5
**DOTAP-0**	0	46	12.5	40	1.5

Formulation	RNP encapsulation rate (%)
**Std**	96 ± 2
**DOTAP-10**	35 ± 2
**DOTAP-5**	10 ± 3
**DOTAP-0**	2 ± 2

Figure S10. Cryo-EM images of FC8 and FX12 LNPs encapsulating iGeoCas9 RNPs.

FC8-formula LNPs encapsulating iGeoCas9 RNP/enhDNA

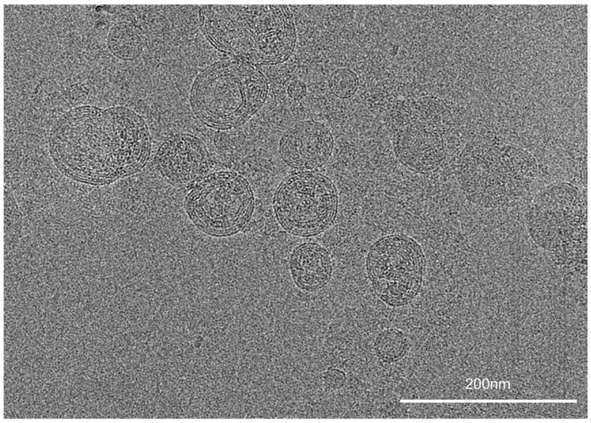

FX12-formula LNPs encapsulating iGeoCas9 RNP/enhDNA

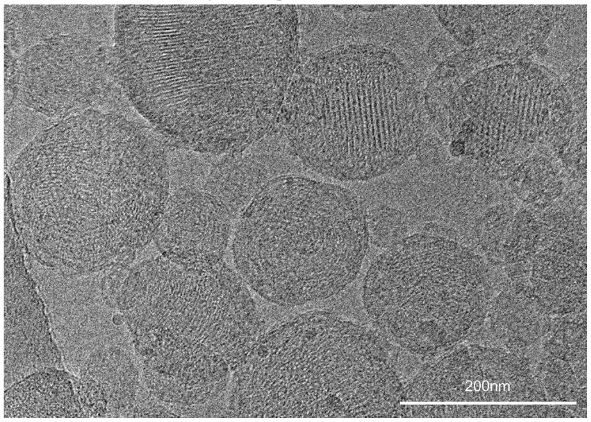

Figure S11. Flow analysis examples of genome-editing activities in the whole livers of Ai9 tdTom mice and different cell types in the liver based on the LNP delivery of iGeoCas9 RNPs using FX12m and FC8m formulations. Cell type analysis is based on immunostaining with corresponding fluorescent-labelled antibodies (hepatocyte – CD95+, macrophage – F4/80+, and endothelial cells – CD31+).

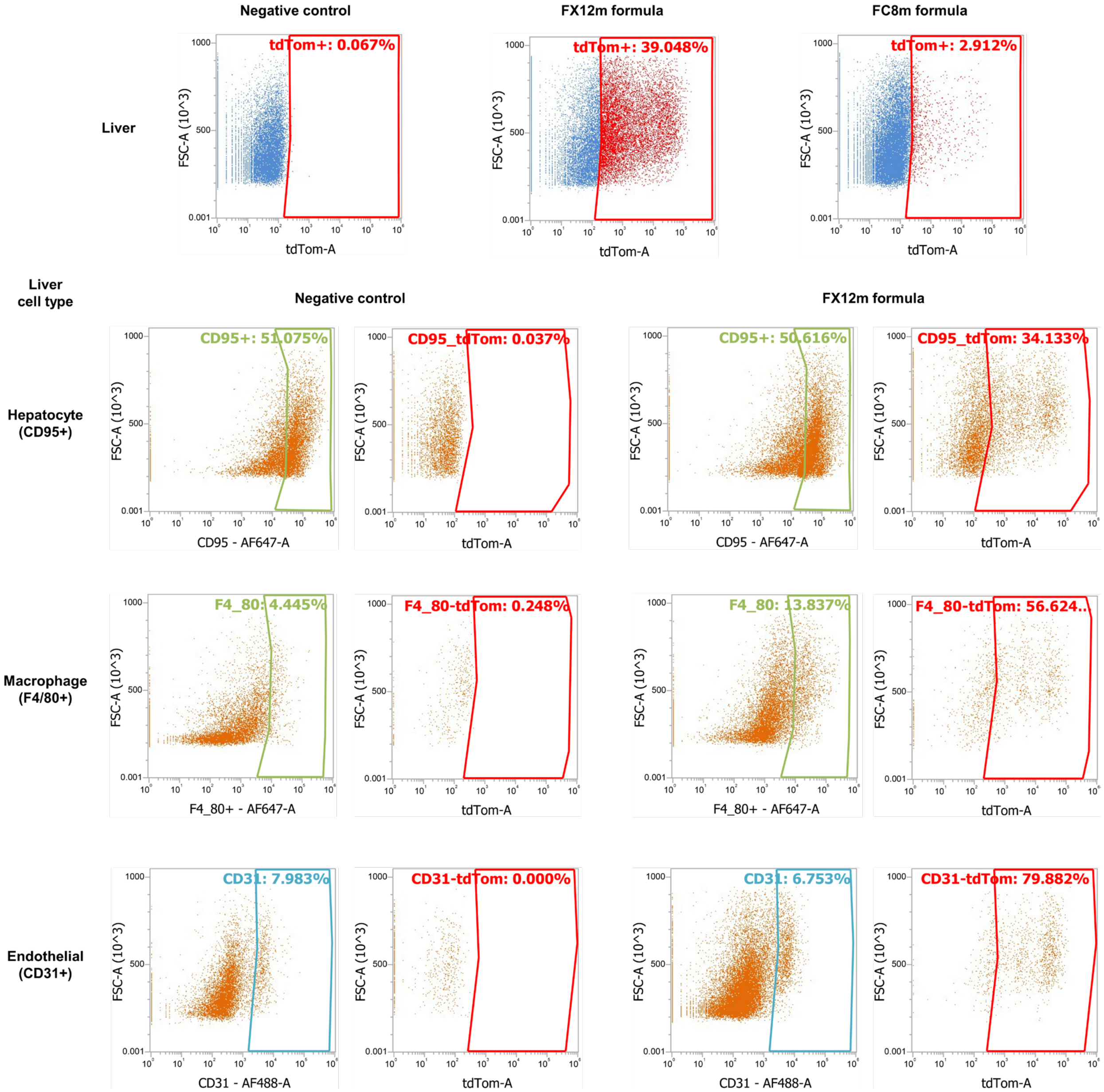

Figure S12. Flow analysis examples of genome-editing activities in the whole lungs of Ai9 tdTom mice and different cell types in the lungs based on the LNP delivery of iGeoCas9 RNPs using FX12m and FC8m formulations. Cell type analysis is based on immunostaining with corresponding fluorescent-labelled antibodies (endothelial cells – CD31+, epithelial cells – CD326+, and immune cells – CD45+).

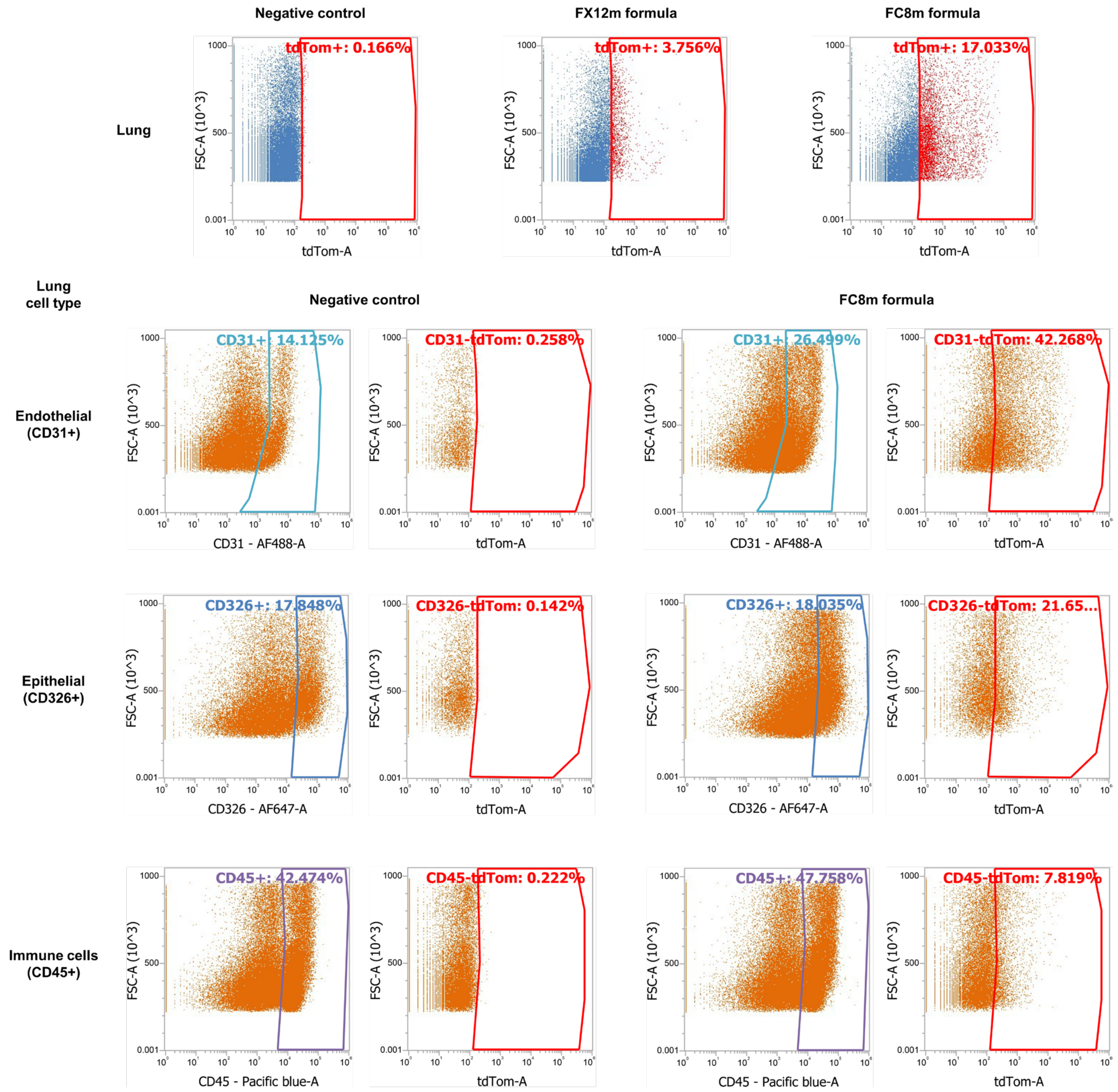

Figure S13. Flow analysis examples of genome-editing activities in different tissues (spleen, kidney and heart) of Ai9 tdTom mice based on the LNP delivery of iGeoCas9 RNPs using FX12m and FC8m formulations.

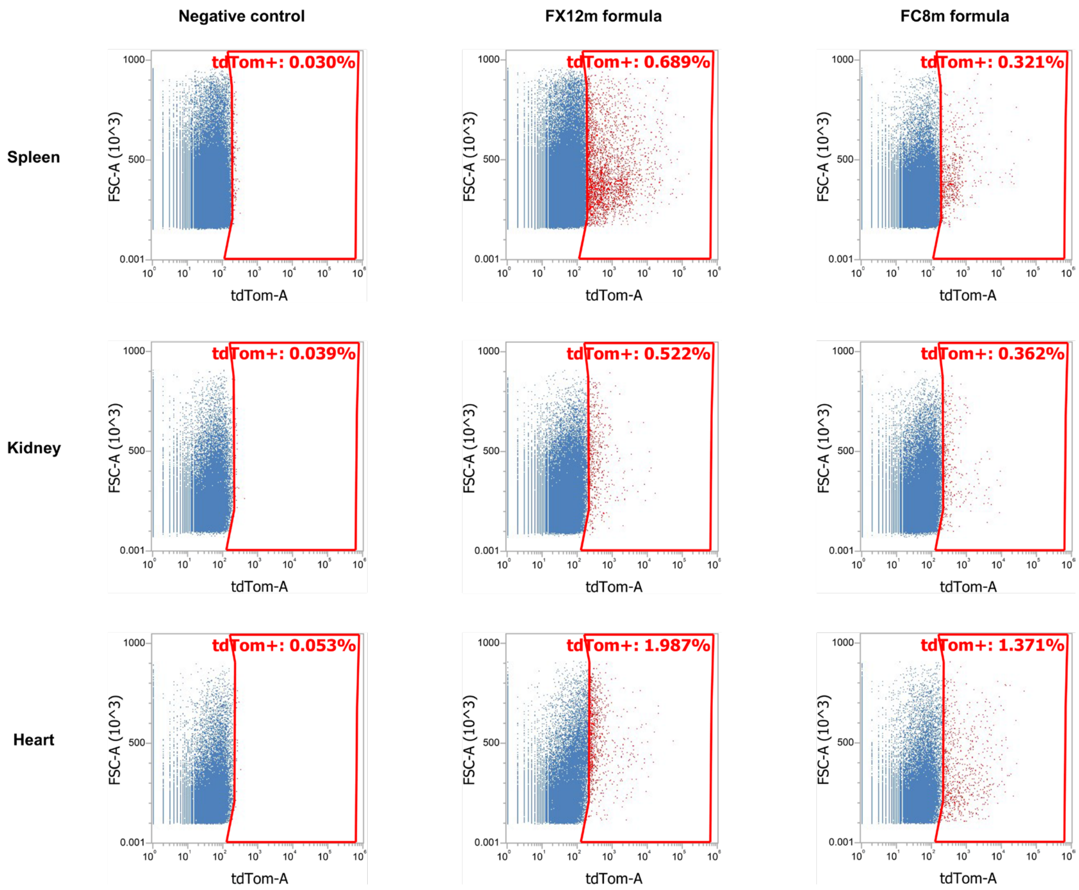

Figure S14. Immune response assessment. **a.** Levels of different serum cytokines at 6 hours post-injection, including interleukin 2 (IL2), interleukin 6 (IL6), tumor necrosis factor α (TNF-α), and macrophage inflammatory protein 2 (MIP-2). **b.** Levels of different serum cytokines at 24 hours post-injection. **c**. Levels of different liver damage enzymes at 2 weeks post-injection, including alanine aminotransferase (ALT), aspartate aminotransferase (AST), and transglutaminase 2 (TGM2). Injections of PBS and LPS (lipopolysaccharide, 1 mg/kg) represent negative and positive controls, respectively; RNP-only (RNP/enhDNA) injections are based on an RNP dosage of 4.6 mg/kg; the remaining injections of empty LNP vectors or RNP:LNP complexes follow the lipid or RNP dosages used for the corresponding mouse experiments. Overall, no significant immune response regarding four cytokines and three liver damage enzymes is observed after the injections of RNP, lipids or RNP:LNPs. LPS injections, as positive controls here, induced significant immune responses at early time points, as indicated by the high levels of three cytokines (IL6, MIP2, and TNF-α), but did not exhibit long-term immunogenicity, as indicated by the normal levels of three liver damage enzymes. n = 3 for each group, data are presented as mean values with individual data points and standard deviations.

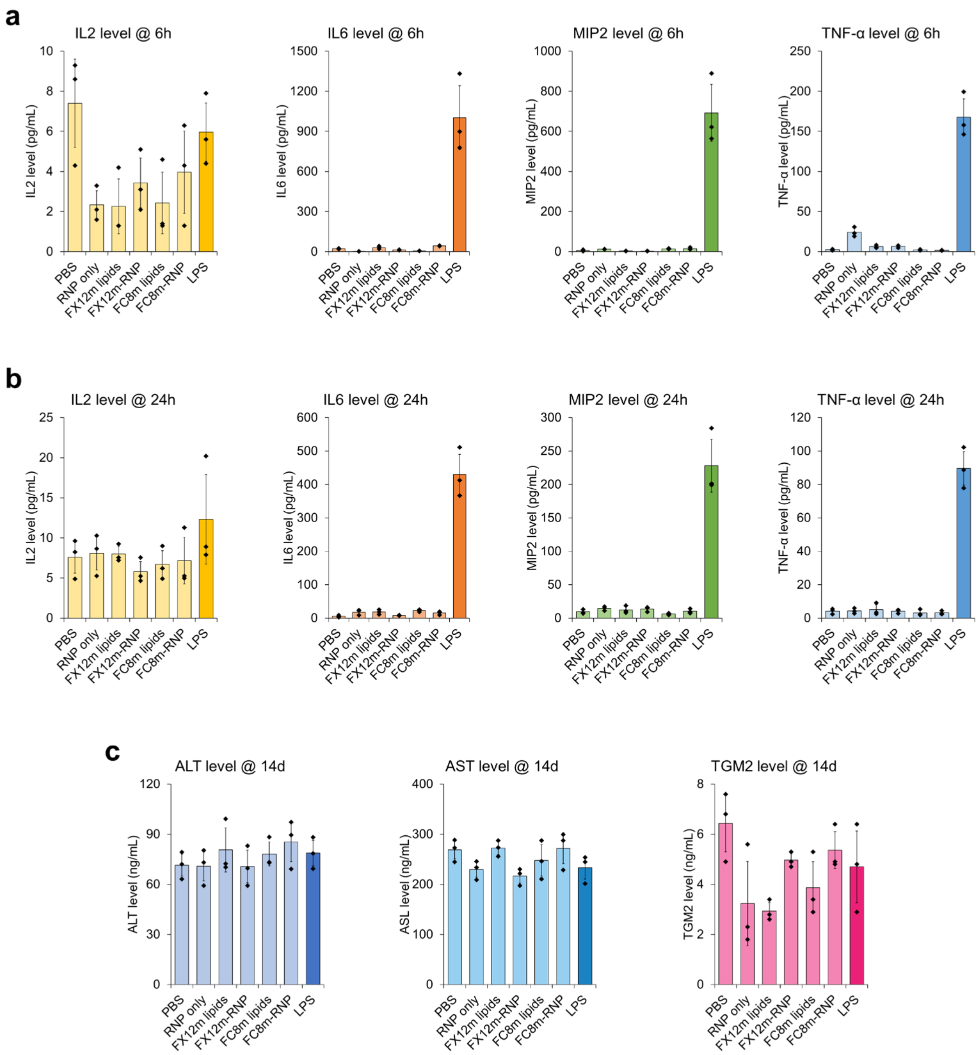

Protein sequences:
GeoCas9, SpyCas9, iCas12a, ThermoCas9, MLV-RT, MBP
SV40 NLS, bipartite NLS, CL7 tag, His tag, Linkers
HRV protease recognition site (↓: protease cleavage position)

Expression construct of 2x-WT-GeoCas9-2x:

MSKSNEPGKATGEGKPVNNKWLNNAGKDLGSPVPDRIANKLRDKEFESFDDFRETFWEEVSKDPELSKQF
SRNNNDRMKVGKAPKTRTQDVSGKRTSFELNHQKPIEQNGGVYDMDNISVVTPKRNIDIEGGGGGSLEVL
FQ↓GPNATPKKRKVGGSPKKRKVGIHGVPAATMRYKIGLDIGITSVGWAVMNLDIPRIEDLGVRIFDRAEN
PQTGESLALPRRLARSARRRLRRRKHRLERIRRLVIREGILTKEELDKLFEEKHEIDVWQLRVEALDRKL
NNDELARVLLHLAKRRGFKSNRKSERSNKENSTMLKHIEENRAILSSYRTVGEMIVKDPKFALHKRNKGE
NYTNTIARDDLEREIRLIFSKQREFGNMSCTEEFENEYITIWASQRPVASKDDIEKKVGFCTFEPKEKRA
PKATYTFQSFIAWEHINKLRLISPSGARGLTDEERRLLYEQAFQKNKITYHDIRTLLHLPDDTYFKGIVY
DRGESRKQNENIRFLELDAYHQIRKAVDKVYGKGKSSSFLPIDFDTFGYALTLFKDDADIHSYLRNEYEQ
NGKRMPNLANKVYDNELIEELLNLSFTKFGHLSLKALRSILPYMEQGEVYSSACERAGYTFTGPKKKQKT
MLLPNIPPIANPVVMRALTQARKVVNAIIKKYGSPVSIHIELARDLSQTFDERRKTKKEQDENRKKNETA
IRQLMEYGLTLNPTGHDIVKFKLWSEQNGRCAYSLQPIEIERLLEPGYVEVDHVIPYSRSLDDSYTNKVL
VLTRENREKGNRIPAEYLGVGTERWQQFETFVLTNKQFSKKKRDRLLRLHYDENEETEFKNRNLNDTRYI
SRFFANFIREHLKFAESDDKQKVYTVNGRVTAHLRSRWEFNKNREESDLHHAVDAVIVACTTPSDIAKVT
AFYQRREQNKELAKKTEPHFPQPWPHFADELRARLSKHPKESIKALNLGNYDDQKLESLQPVFVSRMPKR
SVTGAAHQETLRRYVGIDERSGKIQTVVKTKLSEIKLDASGHFPMYGKESDPRTYEAIRQRLLEHNNDPK
KAFQEPLYKPKKNGEPGPVIRTVKIIDTKNQVIPLNDGKTVAYNSNIVRVDVFEKDGKYYCVPVYTMDIM
KGILPNKAIEPNKPYSEWKEMTEDYTFRFSLYPNDLIRIELPREKTVKTAAGEEINVKDVFVYYKTIDSA
NGGLELISHDHRFSLRGVGSRTLKRFEKYQVDVLGNIYKVRGEKRVGLASSAHSKPGKTIRPLQSTRDGS
PKKKRKVGGSPKKKRKVSLEVLFQ↓GPGSHHHHHH

Expression construct of 2x-GeoCas9(R1)-2x:

MSKSNEPGKATGEGKPVNNKWLNNAGKDLGSPVPDRIANKLRDKEFESFDDFRETFWEEVSKDPELSKQF
SRNNNDRMKVGKAPKTRTQDVSGKRTSFELNHQKPIEQNGGVYDMDNISVVTPKRNIDIEGGGGGSLEVL
FQ↓GPNATPKKRKVGGSPKKRKVGIHGVPAATMRYKIGLDIGITSVGWAVMNLDIPRIEDLGVRIFDRAEN
PQTGESLALPRRLARSARRRLRRRKHRLERIRRLVIREGILTKEELDKLFEEKHEIDVWQLRVEALDRKL
NNDELARVLLHLAKRRGFKSNRKSERSNKENSTMLKHIE**G**NRAILSSYRTVGEMIVKDPKFALHKRNKGE
NY**I**NTIARDDLEREIRLIFSKQREFG**D**MSCTEEFENEYITIWASQRPVASKDDIEKKVGFCTFEPKEKRA
PKATYTFQSFIAWEHINKLRLISPSGARGLTDEERRLLYEQAFQKNKITYHDIRTLLHLPDDTYFKGIVY
DRGESRKQNENIRFLELDAYHQIRKAVDKVYGKGKSSSFLPIDFDTFGYALTLFKDDADIHSYLRNEYEQ
NGKRMPNLANKVYDNELIEELLNLSFTKFGHLSLKALRSILPYMEQGEVYSSACERAGYTFTGPKKKQKT
MLLPNI**Q**PIANPVVMRALTQARKVVNAIIKKYGSPVSIHIELARDLSQTFDERRKTKKEQDENRKKNETA
IRQLMEYGLTLNPTGHDIVKFKLWSEQNGRCAYSLQPIEIERLLEPGYVEVDHVIPYSRSLDDSYTNKVL
VLTRENREKGNRIPAEYLGVGTERWQQFETFVLTNKQFSKKKRDRLLRLHYDENEETEFKNRNLNDTRYI
SRFFANFIREHLKFAESDDKQKVYTVNGRVTAHLRSRWEFNKNREESDLHHAVDAVIVACTTPSDIAKVT
AFYQRREQNKELAKKTEPHFPQPWPHFADELRARLSKHPKESIKALNLGNYDDQKLESLQPVFVSRMPKR
SVTGAAHQETLRRYVGIDERSGKIQTVVKTKLSEIKLDASGHFPMYGKESDPRTYEAIRQRLLEHNNDPK
KAFQEPLYKPKKNGEPGPVIRTVKIIDTKNQVIPLNDGKTVAYNSNIVRVDVFEKDGKYYCVPVYTMDIM
KGILPNKAIEPNKPYSEWKEMTEDYTFRFSLYPNDLIRIELPREKTVKTAAGEEINVKDVFVYYKTIDSA
NGGLELISHDHRFSLRGVGSRTLKRFEKYQVDVLGNIYKVRGEKRVGLASSAHSKPGKTIRPLQSTRDGS
PKKKRKVGGSPKKKRKVSLEVLFQ↓GPGSHHHHHH

Expression construct of 2x-GeoCas9(R1-K)-2x:

MSKSNEPGKATGEGKPVNNKWLNNAGKDLGSPVPDRIANKLRDKEFESFDDFRETFWEEVSKDPELSKQF

SRNNNDRMKVGKAPKTRTQDVSGKRTSFELNHQKPIEQNGGVYDMDNISVVTPKRNIDIEGGGGGSLEVL
FQ↓GPNATPKKRKVGGSPKKRKVGIHGVPAATMRYKIGLDIGITSVGWAVMNLDIPRIEDLGVRIFDRAEN
PQTGESLALPRRLARSARRRLRRRKHRLERIRRLVIREGILTKEELDKLFEEKHEIDVWQLRVEALDRKL
NNDELARVLLHLAKRRGFKSNRKSERSNKENSTMLKHIEGNRAILSSYRTVGEMIVKDPKFALHKRNKGE
NYINTIARDDLEREIRLIFSKQREFGDMSCTEEFENEYITIWASQRPVASKDDIEKKVGFCTFEPKEKRA
PKATYTFQSFIAWEHINKLRLISPSGARGLTDEERRLLYEQAFQKNKITYHDIRTLLHLPDDTYFKGIVY
DRGESRKQNENIRFLELDAYHQIRKAVDKVYGKGKSSSFLPIDFDTFGYALTLFKDDADIHSYLRNEYEQ
NGKRMPNLANKVYDNELIEELLNLSFTKFGHLSLKALRSILPYMEQGEVYSSACERAGYTFTGPKKKQKT
MLLPNIQPIANPVVMRALTQARKVVNAIIKKYGSPVSIHIELARDLSQTFDERRKTKKEQDENRKKNETA
IRQLMEYGLTLNPTGHDIVKFKLWSEQNGRCAYSLQPIEIERLLEPGYVEVDHVIPYSRSLDDSYTNKVL
VLTRENREKGNRIPAEYLGVGTERWQQFETFVLTNKQFSKKKRDRLLRLHYDENEETEFKNRNLNDTRYI
SRFFANFIREHLKFAESDDKQKVYTVNGRVTAHLRSRWEFNKNREESDLHHAVDAVIVACTTPSDIAKVT
AFYQRREQNKELAKKTEPHFPQPWPHFADELRARLSKHPKESIKALNLGNYDDQKLESLQPVFVSRMPKR
SVTGAAHQETLRRYVGIDERSGKIQTVVKTKLSKIKLDASGHFPMYGKESDPRTYEAIRQRLLEHNNDPK
KAFQEPLYKPKKNGEPGPVIRTVKIIDTKNQVIPLNDGKTVAYNSNIVRVDVFEKDGKYYCVPVYTMDIM
KGILPNKAIEPNKPYSEWKEMTEDYTFRFSLYPNDLIRIELPREKTVKTAAGEEINVKDVFVYYKTIDSA
NGGLELISHDHRFSLRGVGSRTLKRFEKYQVDVLGNIYKVRGEKRVGLASSAHSKPGKTIRPLQSTRDGS
PKKKRKVGGSPKKKRKVSLEVLFQ↓GPGSHHHHHH

Expression construct of 2x-GeoCas9(R1-GR)-2x:

MSKSNEPGKATGEGKPVNNKWLNNAGKDLGSPVPDRIANKLRDKEFESFDDFRETFWEEVSKDPELSKQF
SRNNNDRMKVGKAPKTRTQDVSGKRTSFELNHQKPIEQNGGVYDMDNISVVTPKRNIDIEGGGGGSLEVL
FQ↓GPNATPKKRKVGGSPKKRKVGIHGVPAATMRYKIGLDIGITSVGWAVMNLDIPRIEDLGVRIFDRAEN
PQTGESLALPRRLARSARRRLRRRKHRLERIRRLVIREGILTKEELDKLFEEKHEIDVWQLRVEALDRKL
NNDELARVLLHLAKRRGFKSNRKSERSNKENSTMLKHIEGNRAILSSYRTVGEMIVKDPKFALHKRNKGE
NYINTIARDDLEREIRLIFSKQREFGDMSCTEEFENEYITIWASQRPVASKDDIEKKVGFCTFEPKEKRA
PKATYTFQSFIAWEHINKLRLISPSGARGLTDEERRLLYEQAFQKNKITYHDIRTLLHLPDDTYFKGIVY
DRGESRKQNENIRFLELDAYHQIRKAVDKVYGKGKSSSFLPIDFDTFGYALTLFKDDADIHSYLRNEYEQ
NGKRMPNLANKVYDNELIEELLNLSFTKFGHLSLKALRSILPYMEQGEVYSSACERAGYTFTGPKKKQKT
MLLPNIQPIANPVVMRALTQARKVVNAIIKKYGSPVSIHIELARDLSQTFDERRKTKKEQDENRKKNETA
IRQLMEYGLTLNPTGHDIVKFKLWSEQNGRCAYSLQPIEIERLLEPGYVEVDHVIPYSRSLDDSYTNKVL
VLTRENREKGNRIPAEYLGVGTERWQQFETFVLTNKQFSKKKRDRLLRLHYDENEETEFKNRNLNDTRYI
SRFFANFIREHLKFAESDDKQKVYTVNGRVTAHLRSRWEFNKNREESDLHHAVDAVIVACTTPSDIAKVT
AFYQRREQNKELAKKTEPHFPQPWPHFADELRARLSKHPKESIKALNLGNYDDQKLESLQPVFVSRMPKR
SVTGAAHQETLRRYVGIDERSGKIQTVVKTKLSEIKLDASGHFPMYGKESDPRTYEAIRQRLLEHNNDPK
KAFQGPLYKPKKNGEPGPVIRTVKIIDTRNQVIPLNDGKTVAYNSNIVRVDVFEKDGKYYCVPVYTMDIM
KGILPNKAIEPNKPYSEWKEMTEDYTFRFSLYPNDLIRIELPREKTVKTAAGEEINVKDVFVYYKTIDSA
NGGLELISHDHRFSLRGVGSRTLKRFEKYQVDVLGNIYKVRGEKRVGLASSAHSKPGKTIRPLQSTRDGS
PKKKRKVGGSPKKKRKVSLEVLFQ↓GPGSHHHHHH

Expression construct of 2x-GeoCas9(R1-GRK)-2x:

MSKSNEPGKATGEGKPVNNKWLNNAGKDLGSPVPDRIANKLRDKEFESFDDFRETFWEEVSKDPELSKQF
SRNNNDRMKVGKAPKTRTQDVSGKRTSFELNHQKPIEQNGGVYDMDNISVVTPKRNIDIEGGGGGSLEVL
FQ↓GPNATPKKRKVGGSPKKRKVGIHGVPAATMRYKIGLDIGITSVGWAVMNLDIPRIEDLGVRIFDRAEN
PQTGESLALPRRLARSARRRLRRRKHRLERIRRLVIREGILTKEELDKLFEEKHEIDVWQLRVEALDRKL
NNDELARVLLHLAKRRGFKSNRKSERSNKENSTMLKHIEGNRAILSSYRTVGEMIVKDPKFALHKRNKGE
NYINTIARDDLEREIRLIFSKQREFGDMSCTEEFENEYITIWASQRPVASKDDIEKKVGFCTFEPKEKRA
PKATYTFQSFIAWEHINKLRLISPSGARGLTDEERRLLYEQAFQKNKITYHDIRTLLHLPDDTYFKGIVY
DRGESRKQNENIRFLELDAYHQIRKAVDKVYGKGKSSSFLPIDFDTFGYALTLFKDDADIHSYLRNEYEQ
NGKRMPNLANKVYDNELIEELLNLSFTKFGHLSLKALRSILPYMEQGEVYSSACERAGYTFTGPKKKQKT

MLLPNIQPIANPVVMRALTQARKVVNAIIKKYGSPVSIHIELARDLSQTFDERRKTKKEQDENRKKNETA
IRQLMEYGLTLNPTGHDIVKFKLWSEQNGRCAYSLQPIEIERLLEPGYVEVDHVIPYSRSLDDSYTNKVL
VLTRENREKGNRIPAEYLGVGTERWQQFETFVLTNKQFSKKKRDRLLRLHYDENEETEFKNRNLNDTRYI
SRFFANFIREHLKFAESDDKQKVYTVNGRVTAHLRSRWEFNKNREESDLHHAVDAVIVACTTPSDIAKVT
AFYQRREQNKELAKKTEPHFPQPWPHFADELRARLSKHPKESIKALNLGNYDDQKLESLQPVFVSRMPKR
SVTGAAHQETLRRYVGIDERSGKIQTVVKTKLSKIKLDASGHFPMYGKESDPRTYEAIRQRLLEHNNDPK
KAFQGPLYKPKKNGEPGPVIRTVKIIDTRNQVIPLNDGKTVAYNSNIVRVDVFEKDGKYYCVPVYTMDIM
KGILPNKAIEPNKPYSEWKEMTEDYTFRFSLYPNDLIRIELPREKTVKTAAGEEINVKDVFVYYKTIDSA
NGGLELISHDHRFSLRGVGSRTLKRFEKYQVDVLGNIYKVRGEKRVGLASSAHSKPGKTIRPLQSTRDGS
PKKKRKVGGSPKKKRKVSLEVLFQ↓GPGSHHHHHH

Expression construct of 2x-GeoCas9(R1W1)-2x:

MSKSNEPGKATGEGKPVNNKWLNNAGKDLGSPVPDRIANKLRDKEFESFDDFRETFWEEVSKDPELSKQF
SRNNNDRMKVGKAPKTRTQDVSGKRTSFELNHQKPIEQNGGVYDMDNISVVTPKRNIDIEGGGGGSLEVL
FQ↓GPNATPKKRKVGGSPKKRKVGIHGVPAATMRYKIGLDIGITSVGWAVMNLDIPRIEDLGVRIFDRAEN
PQTGESLALPRRLARSARRRLRRRKHRLERIRRLVIREGILTKEELDKLFEEKHEIDVWQLRVEALDRKL
NNDELARVLLHLAKRRGFKSNRKSERSNKENSTMLKHIEGNRAILSSYRTVGEMIVKDPKFALHKRNKGE
NYINTIARDDLEREIRLIFSKQREFGDMSCTEEFENEYITIWASQRPVASKDDIEKKVGFCTFEPKEKRA
PKATYTFQSFIAWEHINKLRLISPSGARGLTDEERRLLYEQAFQKNKITYHDIRTLLHLPDDTYFKGIVY
DRGESRKQNENIRFLELDAYHQIRKAVDKVYGKGKSSSFLPIDFDTFGYALTLFKDDADIHSYLRNEYEQ
NGKRMPNLANKVYDNELIEELLNLSFTKFGHLSLKALRSILPYMEQGEVYSSACERAGYTFTGPKKKQKT
MLLPNIQPIANPVVMRALTQARKVVNAIIKKYGSPVSIHIELARDLSQTFDERRKTKKEQDENRKKNETA
IRQLMEYGLTLNPTGHDIVKFKLWSEQNGRCAYSLQPIEIERLLEPGYVEVDHVIPYSRSLDDSYTNKVL
VLTRENREKGNRIPAEYLGVGTERWQQFETFVLTNKQFSKKKRDRLLRLHYDENEETEFKNRNLNDTRYI
SRFFANFIREHLKFAESDDKQKVYTVNGRVTAHLRSRWEFNKNREESDLHHAVDAVIVACTTPSDIAKVT
AFYQRREQNKELAKKTEPHFPQPWPHFADELRARLSKHPKESIKALNLGNYDDQKLESLQPVFVSRMPKR
SVTGAAHRETLRRYVGIDERSGKIQTVVKTKLSKIKLDASGHFPMYGKESDPRTYEAIRQRLLEHNNDPK
KAFQGPLYKPKKNGEPGPVIRTVKIIDTRNQVIPLNDGKTVAYNSNIVRVDVFEKDGKYYCVPVYTMDIM
KGILPNKAIEPNKPYSEWKEMTEDYTFRFSLYPNDLIRIELPREKTVKTAAGEEINVKDVFVYYKTIDSA
NGGLELISHDHRFSLRGVGSRTLKRFEKYQVDVLGNIYKVRGEKRVGLASSAHSKPGKTIRPLQSTRDGS
PKKKRKVGGSPKKKRKVSLEVLFQ↓GPGSHHHHHH

Expression construct of 2x-GeoCas9(R1WP1)-2x:

MSKSNEPGKATGEGKPVNNKWLNNAGKDLGSPVPDRIANKLRDKEFESFDDFRETFWEEVSKDPELSKQF
SRNNNDRMKVGKAPKTRTQDVSGKRTSFELNHQKPIEQNGGVYDMDNISVVTPKRNIDIEGGGGGSLEVL
FQ↓GPNATPKKRKVGGSPKKRKVGIHGVPAATMRYKIGLDIGITSVGWAVMNLDIPRIEDLGVRIFDRAEN
PQTGESLALPRRLARSARRRLRRRKHRLERIRRLVIREGILTKEELDKLFEEKHEIDVWQLRVEALDRKL
NNDELARVLLHLAKRRGFKSNRKSERSNKENSTMLKHIEGNRAILSSYRTVGEMIVKDPKFALHKRNKGE
NYINTIARDDLEREIRLIFSKQREFGDMSCTEEFENEYITIWASQRPVASKDDIEKKVGFCTFEPKEKRA
PKATYTFQSFIAWEHINKLRLISPSGARGLTDEERRLLYEQAFQKNKITYHDIRTLLHLPDDTYFKGIVY
DRGESRKQNENIRFLELDAYHQIRKAVDKVYGKGKSSSFLPIDFDTFGYALTLFKDDADIHSYLRNEYEQ
NGKRMPNLANKVYDNELIEELLNLSFTKFGHLSLKALRSILPYMEQGEVYSSACERAGYTFTGPKKKQKT
MLLPNIQPIANPVVMRALTQARKVVNAIIKKYGSPVSIHIELARDLSQTFDERRKTKKEQDENRKKNETA
IRQLMEYGLTLNPTGHDIVKFKLWSEQNGRCAYSLQPIEIERLLEPGYVEVDHVIPYSRSLDDSYTNKVL
VLTRENREKGNRIPAEYLGVGTERWQQFETFVLTNKQFSKKKRDRLLRLHYDENEETEFKNRNLNDTRYI
SRFFANFIREHLKFAESDDKQKYTVNGRVTAHLRSRWEFNKNREESDLHHAVDAVIVACTTPSDIAKVTA
FYQRREQNKELAKKTEPHFPQPWPHFADELRARLSKHPKESIKALNLGNYDDQKLESLQPVFVSRMPKRS
VTGAAHQETLRRYVGIDERSGKIQTVVKTKLSKIKLDASGHFPMYGKESDPRTYEAIRQRLLEHNNDPKK
AFQGPLYKPKKNGEPGPVIRTVKIIDTRNQVIPLNDGKTVAYNSNIVRVDVFEKDGKYYCVPVYTMDIMK
GILPNKAIEPNKPYSEWKEMTEDYTFRFSLYPNDLIRIELPREKTVKTAAGEEINVKDVFVYYKAINSAN

GGLELISHDHRFSLRGVGSRTLKRFEKYQVDVLGNIYKVRGEKRVGLASSAHSKPGKTIRPLQSTRDGSP
KKKRKVGGSPKKKRKVSLEVLFQ↓GPGSHHHHHH

Expression construct of 1x-GeoCas9(R1W1)-2x:

MKSSHHHHHHGSSKSNEPGKATGEGKPVNNKWLNNAGKDLGSPVPDRIANKLRDKEFESFDDFRETFWEE
VSKDPELSKQFSRNNNDRMKVGKAPKTRTQDVSGKRTSFELNHQKPIEQNGGVYDMDNISVVTPKRNIDI
EGGGGGSGGSMKIEEGKLVIWINGDKGYNGLAEVGKKFEKDTGIKVTVEHPDKLEEKFPQVAATGDGPDI
IFWAHDRFGGYAQSGLLAEITPDKAFQDKLYPFTWDAVRYNGKLIAYPIAVEALSLIYNKDLLPNPPKTW
EEIPALDKELKAKGKSALMFNLQEPYFTWPLIAADGGYAFKYENGKYDIKDVGVDNAGAKAGLTFLVDLI
KNKHMNADTDYSIAEAAFNKGETAMTINGPWAWSNIDTSKVNYGVTVLPTFKGQPSKPFVGVLSAGINAA
SPNKELAKEFLENYLLTDEGLEAVNKDKPLGAVALKSYEEELAKDPRIAATMENAQKGEIMPNIPQMSAF
WYAVRTAVINAASGRQTVDEALKDAQTNSSSNNNNNNNNNNLGIELEVLFQ↓GPGSPKKRKVGGSNAMRYK
IGLDIGITSVGWAVMNLDIPRIEDLGVRIFDRAENPQTGESLALPRRLARSARRRLRRRKHRLERIRRLV
IREGILTKEELDKLFEEKHEIDVWQLRVEALDRKLNNDELARVLLHLAKRRGFKSNRKSERSNKENSTML
KHIEGNRAILSSYRTVGEMIVKDPKFALHKRNKGENYINTIARDDLEREIRLIFSKQREFGDMSCTEEFE
NEYITIWASQRPVASKDDIEKKVGFCTFEPKEKRAPKATYTFQSFIAWEHINKLRLISPSGARGLTDEER
RLLYEQAFQKNKITYHDIRTLLHLPDDTYFKGIVYDRGESRKQNENIRFLELDAYHQIRKAVDKVYGKGK
SSSFLPIDFDTFGYALTLFKDDADIHSYLRNEYEQNGKRMPNLANKVYDNELIEELLNLSFTKFGHLSLK
ALRSILPYMEQGEVYSSACERAGYTFTGPKKKQKTMLLPNIQPIANPVVMRALTQARKVVNAIIKKYGSP
VSIHIELARDLSQTFDERRKTKKEQDENRKKNETAIRQLMEYGLTLNPTGHDIVKFKLWSEQNGRCAYSL
QPIEIERLLEPGYVEVDHVIPYSRSLDDSYTNKVLVLTRENREKGNRIPAEYLGVGTERWQQFETFVLTN
KQFSKKKRDRLLRLHYDENEETEFKNRNLNDTRYISRFFANFIREHLKFAESDDKQKVYTVNGRVTAHLR
SRWEFNKNREESDLHHAVDAVIVACTTPSDIAKVTAFYQRREQNKELAKKTEPHFPQPWPHFADELRARL
SKHPKESIKALNLGNYDDQKLESLQPVFVSRMPKRSVTGAAHRETLRRYVGIDERSGKIQTVVKTKLSKI
KLDASGHFPMYGKESDPRTYEAIRQRLLEHNNDPKKAFQGPLYKPKKNGEPGPVIRTVKIIDTRNQVIPL
NDGKTVAYNSNIVRVDVFEKDGKYYCVPVYTMDIMKGILPNKAIEPNKPYSEWKEMTEDYTFRFSLYPND
LIRIELPREKTVKTAAGEEINVKDVFVYYKTIDSANGGLELISHDHRFSLRGVGSRTLKRFEKYQVDVLG
NIYKVRGEKRVGLASSAHSKPGKTIRPLQSTRDGSPKKKRKVGGSPKKKRKV

Expression construct of 2x-WT-ThermoCas9-2x:

MSKSNEPGKATGEGKPVNNKWLNNAGKDLGSPVPDRIANKLRDKEFESFDDFRETFWEEVSKDPELSKQF
SRNNNDRMKVGKAPKTRTQDVSGKRTSFELNHQKPIEQNGGVYDMDNISVVTPKRNIDIEGGGGGSLEVL
FQ↓GPNATPKKRKVGGSPKKRKVGIHGVPAATMKYKIGLDIGITSIGWAVINLDIPRIEDLGVRIFDRAEN
PKTGESLALPRRLARSARRRLRRRKHRLERIRRLFVREGILTKEELNKLFEKKHEIDVWQLRVEALDRKL
NNDELARILLHLAKRRGFRSNRKSERTNKENSTMLKHIEENQSILSSYRTVAEMVVKDPKFSLHKRNKED
NYTNTVARDDLEREIKLIFAKQREYGNIVCTEAFEHEYISIWASQRPFASKDDIEKKVGFCTFEPKEKRA
PKATYTFQSFTVWEHINKLRLVSPGGIRALTDDERRLIYKQAFHKNKITFHDVRTLLNLPDDTRFKGLLY
DRNTTLKENEKVRFLELGAYHKIRKAIDSVYGKGAAKSFRPIDFDTFGYALTMFKDDTDIRSYLRNEYEQ
NGKRMENLADKVYDEELIEELLNLSFSKFGHLSLKALRNILPYMEQGEVYSTACERAGYTFTGPKKKQKT
VLLPNIPPIANPVVMRALTQARKVVNAIIKKYGSPVSIHIELARELSQSFDERRKMQKEQEGNRKKNETA
IRQLVEYGLTLNPTGLDIVKFKLWSEQNGKCAYSLQPIEIERLLEPGYTEVDHVIPYSRSLDDSYTNKVL
VLTKENREKGNRTPAEYLGLGSERWQQFETFVLTNKQFSKKKRDRLLRLHYDENEENEFKNRNLNDTRYI
SRFLANFIREHLKFADSDDKQKVYTVNGRITAHLRSRWNFNKNREESNLHHAVDAAIVACTTPSDIARVT
AFYQRREQNKELSKKTDPQFPQPWPHFADELQARLSKNPKESIKALNLGNYDNEKLESLQPVFVSRMPKR
SITGAAHQETLRRYIGIDERSGKIQTVVKKKLSEIQLDKTGHFPMYGKESDPRTYEAIRQRLLEHNNDPK
KAFQEPLYKPKKNGELGPIIRTIKIIDTTNQVIPLNDGKTVAYNSNIVRVDVFEKDGKYYCVPIYTIDMM
KGILPNKAIEPNKPYSEWKEMTEDYTFRFSLYPNDLIRIEFPREKTIKTAVGEEIKIKDLFAYYQTIDSS
NGGLSLVSHDNNFSLRSIGSRTLKRFEKYQVDVLGNIYKVRGEKRVGVASSSHSKAGETIRPGSPKKKRK
VGGSPKKKRKVSLEVLFQ↓GPGSHHHHHH

Expression construct of 2x-ThermoCas9(R1W1)-2x:

MSKSNEPGKATGEGKPVNNKWLNNAGKDLGSPVPDRIANKLRDKEFESFDDFRETFWEEVSKDPELSKQF
SRNNNDRMKVGKAPKTRTQDVSGKRTSFELNHQKPIEQNGGVYDMDNISVVTPKRNIDIEGGGGGSLEVL
FQ↓GPNATPKKRKVGGSPKKRKVGIHGVPAATMKYKIGLDIGITSIGWAVINLDIPRIEDLGVRIFDRAEN
PKTGESLALPRRLARSARRRLRRRKHRLERIRRLFVREGILTKEELNKLFEKKHEIDVWQLRVEALDRKL
NNDELARILLHLAKRRGFRSNRKSERTNKENSTMLKHIE**G**NQSILSSYRTVAEMVVKDPKFSLHKRNKED
NY**I**NTVARDDLEREIKLIFAKQREYG**D**IVCTEAFEHEYISIWASQRPFASKDDIEKKVGFCTFEPKEKRA
PKATYTFQSFTVWEHINKLRLVSPGGIRALTDDERRLIYKQAFHKNKITFHDVRTLLNLPDDTRFKGLLY
DRNTTLKENEKVRFLELGAYHKIRKAIDSVYGKGAAKSFRPIDFDTFGYALTMFKDDTDIRSYLRNEYEQ
NGKRMENLADKVYDEELIEELLNLSFSKFGHLSLKALRNILPYMEQGEVYSTACERAGYTFTGPKKKQKT
VLLPNI**Q**PIANPVVMRALTQARKVVNAIIKKYGSPVSIHIELARELSQSFDERRKMQKEQEGNRKKNETA
IRQLVEYGLTLNPTGLDIVKFKLWSEQNGKCAYSLQPIEIERLLEPGYTEVDHVIPYSRSLDDSYTNKVL
VLTKENREKGNRTPAEYLGLGSERWQQFETFVLTNKQFSKKKRDRLLRLHYDENEENEFKNRNLNDTRYI
SRFLANFIREHLKFADSDDKQKVYTVNGRITAHLRSRWNFNKNREESNLHHAVDAAIVACTTPSDIARVT
AFYQRREQNKELSKKTDPQFPQPWPHFADELQARLSKNPKESIKALNLGNYDNEKLESLQPVFVSRMPKR
SITGAAH**R**ETLRRYIGIDERSGKIQTVVKKKLS**K**IQLDKTGHFPMYGKESDPRTYEAIRQRLLEHNNDPK
KAFQ**G**PLYKPKKNGELGPIIRTIKIIDT**R**NQVIPLNDGKTVAYNSNIVRVDVFEKDGKYYCVPIYTIDMM
KGILPNKAIEPNKPYSEWKEMTEDYTFRFSLYPNDLIRIEFPREKTIKTAVGEEIKIKDLFAYYQTIDSS
NGGLSLVSHDNNFSLRSIGSRTLKRFEKYQVDVLGNIYKVRGEKRVGVASSSHSKAGETIRPGSPKKKRK
VGGSPKKKRKVSLEVLFQ↓GPGSHHHHHH

Expression construct of 2x-SpyCas9-2x:

MSKSNEPGKATGEGKPVNNKWLNNAGKDLGSPVPDRIANKLRDKEFESFDDFRETFWEEVSKDPELSKQF
SRNNNDRMKVGKAPKTRTQDVSGKRTSFELNHQKPIEQNGGVYDMDNISVVTPKRNIDIEGGGGGSLEVL
FQ↓GPNATPKKRKVGGSPKKRKVGIHGVPAATMDKKYSIGLDIGTNSVGWAVITDEYKVPSKKFKVLGNTD
RHSIKKNLIGALLFDSGETAEATRLKRTARRRYTRRKNRICYLQEIFSNEMAKVDDSFFHRLEESFLVEE
DKKHERHPIFGNIVDEVAYHEKYPTIYHLRKKLVDSTDKADLRLIYLALAHMIKFRGHFLIEGDLNPDNS
DVDKLFIQLVQTYNQLFEENPINASGVDAKAILSARLSKSRRLENLIAQLPGEKKNGLFGNLIALSLGLT
PNFKSNFDLAEDAKLQLSKDTYDDDLDNLLAQIGDQYADLFLAAKNLSDAILLSDILRVNTEITKAPLSA
SMIKRYDEHHQDLTLLKALVRQQLPEKYKEIFFDQSKNGYAGYIDGGASQEEFYKFIKPILEKMDGTEEL
LVKLNREDLLRKQRTFDNGSIPHQIHLGELHAILRRQEDFYPFLKDNREKIEKILTFRIPYYVGPLARGN
SRFAWMTRKSEETITPWNFEEVVDKGASAQSFIERMTNFDKNLPNEKVLPKHSLLYEYFTVYNELTKVKY
VTEGMRKPAFLSGEQKKAIVDLLFKTNRKVTVKQLKEDYFKKIECFDSVEISGVEDRFNASLGTYHDLLK
IIKDKDFLDNEENEDILEDIVLTLTLFEDREMIEERLKTYAHLFDDKVMKQLKRRRYTGWGRLSRKLING
IRDKQSGKTILDFLKSDGFANRNFMQLIHDDSLTFKEDIQKAQVSGQGDSLHEHIANLAGSPAIKKGILQ
TVKVVDELVKVMGRHKPENIVIEMARENQTTQKGQKNSRERMKRIEEGIKELGSQILKEHPVENTQLQNE
KLYLYYLQNGRDMYVDQELDINRLSDYDVDHIVPQSFLKDDSIDNKVLTRSDKNRGKSDNVPSEEVVKKM
KNYWRQLLNAKLITQRKFDNLTKAERGGLSELDKAGFIKRQLVETRQITKHVAQILDSRMNTKYDENDKL
IREVKVITLKSKLVSDFRKDFQFYKVREINNYHHAHDAYLNAVVGTALIKKYPKLESEFVYGDYKVYDVR
KMIAKSEQEIGKATAKYFFYSNIMNFFKTEITLANGEIRKRPLIETNGETGEIVWDKGRDFATVRKVLSM
PQVNIVKKTEVQTGGFSKESILPKRNSDKLIARKKDWDPKKYGGFDSPTVAYSVLVVAKVEKGKSKKLKS
VKELLGITIMERSSFEKNPIDFLEAKGYKEVKKDLIIKLPKYSLFELENGRKRMLASAGELQKGNELALP
SKYVNFLYLASHYEKLKGSPEDNEQKQLFVEQHKHYLDEIIEQISEFSKRVILADANLDKVLSAYNKHRD
KPIREQAENIIHLFTLTNLGAPAAFKYFDTTIDRKRYTSTKEVLDATLIHQSITGLYETRIDLSQLGGDG
SPKKKRKVEDPKKKRKVSLEVLFQ↓GPGSHHHHHH

Expression construct of 2x-iCas12a-2x:

MSKSNEPGKATGEGKPVNNKWLNNAGKDLGSPVPDRIANKLRDKEFESFDDFRETFWEEVSKDPELSKQF
SRNNNDRMKVGKAPKTRTQDVSGKRTSFELNHQKPIEQNGGVYDMDNISVVTPKRNIDIEGGGGGSLEVL
FQ↓GPNATPKKRKVGGSPKKRKVGIHGVPAATMSKLEKFTNCYSLSKTLRFKAIPVGKTQENIDNKRLLVE

DEKRAEDYKGVKKLLDRYYLSFINDVLHSIKLKNLNNYISLFRKKTRTEKENKELENLEINLRKEIAKAF
KGNEGYKSLFKKDIIETILPEFLDDKDEIALVNSFNGFTTAFTGFFDNRENMFSEEAKSTSIAFRCINEN
LTRYISNMDIFEKVDAIFDKHEVQEIKEKILNSDYDVEDFFEGEFFNFVLTQEGIDVYNAIIGGFVTESG
EKIKGLNEYINLYNQKTKQKLPKFKPLYKQVLSDRESLSFYGEGYTSDEEVLEVFRNTLNKNSEIFSSIK
KLEKLFKNFDEYSSAGIFVKNGPAISTISKDIFGEWNVIRDKWNAEYDDIHLKKKAVVTEKYEDDRRKSF
KKIGSFSLEQLQEYADADLSVVEKLKEIIIQKVDEIYKVYGSSEKLFDADFVLEKSLKKNDAVVAIMKDL
LDSVKSFENYIKAFFGEGKETNRDESFYGDFVLAYDILLKVDHIYDAIRNYVTQKPYSKDKFKLYFQNPQ
FMGGWDKDKETDYRATILRYGSKYYLAIMDKKYAKCLQKIDKDDVNGNYEKINYKLLPGPNKMLPKVFFS
KKWMAYYNPSEDIQKIYKNGTFKKGDMFNLNDCHKLIDFFKDSISRYPKWSNAYDFNFSETEKYKDIAGF
YREVEEQGYKVSFESASKKEVDKLVEEGKLYMFQIYNKDFSDKSHGTPNLHTMYFKLLFDENNHGQIRLS
GGAELFMRRASLKKEELVVHPANSPIANKNPDNPKKTTTLSYDVYKDKRFSEDQYELHIPIAINKCPKNI
FKINTEVRVLLKHDDNPYVIGIDRGERNLLYIVVVDGKGNIVEQYSLNEIINNVNGIRIKTDYHSLLDKK
EKERFEARQNWTSIENIKELKAGYISQVVHKICELVEKYDAVIALEDLNSGFKNSRVKVEKQVYQKFEKM
LINKLNYMVDKKSNPYATGGALKGYQITNKFESFKSMSTQNGFIFYIPAWLTSKIDPSTGFANLLKTKYT
SIADSKKFISSFDRIMYVPEEDLFEFALDYKNFSRTDADYIKKWKLYSYGNRIRIFRNPKKNNVFDWEEV
CLTSAYKELFNKYGINYQLGDIRVLLCEQSDKAFYSSFMALMTLMLQMRNSITGRTDVDFLISPVKNSDG
IFYDSRNYEAQENAILPKNADANGAYNIARKVLWAIGQFKKAEDEKLDKVKIAIPNKEWLEYAQTSVKHG
GSPKKRKVGGSPKKRKVSLEVLFQ↓GPGSHHHHHH

Expression construct of SpyCas9-based prime editor (PE2):

MSKSNEPGKATGEGKPVNNKWLNNAGKDLGSPVPDRIANKLRDKEFESFDDFRETFWEEVSKDPELSKQF
SRNNNDRMKVGKAPKTRTQDVSGKRTSFELNHQKPIEQNGGVYDMDNISVVTPKRNIDIEGGGGGSLEVL
FQ↓GPNATKRTADGSEFESPKKKRKVDKKYSIGLDIGTNSVGWAVITDEYKVPSKKFKVLGNTDRHSIKK
NLIGALLFDSGETAEATRLKRTARRRYTRRKNRICYLQEIFSNEMAKVDDSFFHRLEESFLVEEDKKHER
HPIFGNIVDEVAYHEKYPTIYHLRKKLVDSTDKADLRLIYLALAHMIKFRGHFLIEGDLNPDNSDVDKLF
IQLVQTYNQLFEENPINASGVDAKAILSARLSKSRRLENLIAQLPGEKKNGLFGNLIALSLGLTPNFKSN
FDLAEDAKLQLSKDTYDDDLDNLLAQIGDQYADLFLAAKNLSDAILLSDILRVNTEITKAPLSASMIKRY
DEHHQDLTLLKALVRQQLPEKYKEIFFDQSKNGYAGYIDGGASQEEFYKFIKPILEKMDGTEELLVKLNR
EDLLRKQRTFDNGSIPHQIHLGELHAILRRQEDFYPFLKDNREKIEKILTFRIPYYVGPLARGNSRFAWM
TRKSEETITPWNFEEVVDKGASAQSFIERMTNFDKNLPNEKVLPKHSLLYEYFTVYNELTKVKYVTEGMR
KPAFLSGEQKKAIVDLLFKTNRKVTVKQLKEDYFKKIECFDSVEISGVEDRFNASLGTYHDLLKIIKDKD
FLDNEENEDILEDIVLTLTLFEDREMIEERLKTYAHLFDDKVMKQLKRRRYTGWGRLSRKLINGIRDKQS
GKTILDFLKSDGFANRNFMQLIHDDSLTFKEDIQKAQVSGQGDSLHEHIANLAGSPAIKKGILQTVKVVD
ELVKVMGRHKPENIVIEMARENQTTQKGQKNSRERMKRIEEGIKELGSQILKEHPVENTQLQNEKLYLYY
LQNGRDMYVDQELDINRLSDYDVDAIVPQSFLKDDSIDNKVLTRSDKNRGKSDNVPSEEVVKKMKNYWRQ
LLNAKLITQRKFDNLTKAERGGLSELDKAGFIKRQLVETRQITKHVAQILDSRMNTKYDENDKLIREVKV
ITLKSKLVSDFRKDFQFYKVREINNYHHAHDAYLNAVVGTALIKKYPKLESEFVYGDYKVYDVRKMIAKS
EQEIGKATAKYFFYSNIMNFFKTEITLANGEIRKRPLIETNGETGEIVWDKGRDFATVRKVLSMPQVNIV
KKTEVQTGGFSKESILPKRNSDKLIARKKDWDPKKYGGFDSPTVAYSVLVVAKVEKGKSKKLKSVKELLG
ITIMERSSFEKNPIDFLEAKGYKEVKKDLIIKLPKYSLFELENGRKRMLASAGELQKGNELALPSKYVNF
LYLASHYEKLKGSPEDNEQKQLFVEQHKHYLDEIIEQISEFSKRVILADANLDKVLSAYNKHRDKPIREQ
AENIIHLFTLTNLGAPAAFKYFDTTIDRKRYTSTKEVLDATLIHQSITGLYETRIDLSQLGGDSGGSSGG
SSGSETPGTSESATPESSGGSSGGSSTLNIEDEYRLHETSKEPDVSLGSTWLSDFPQAWAETGGMGLAVR
QAPLIIPLKATSTPVSIKQYPMSQEARLGIKPHIQRLLDQGILVPCQSPWNTPLLPVKKPGTNDYRPVQD
LREVNKRVEDIHPTVPNPYNLLSGLPPSHQWYTVLDLKDAFFCLRLHPTSQPLFAFEWRDPEMGISGQLT
WTRLPQGFKNSPTLFNEALHRDLADFRIQHPDLILLQYVDDLLLAATSELDCQQGTRALLQTLGNLGYRA
SAKKAQICQKQVKYLGYLLKEGQRWLTEARKETVMGQPTPKTPRQLREFLGKAGFCRLFIPGFAEMAAPL
YPLTKPGTLFNWGPDQQKAYQEIKQALLTAPALGLPDLTKPFELFVDEKQGYAKGVLTQKLGPWRRPVAY
LSKKLDPVAAGWPPCLRMVAAIAVLTKDAGKLTMGQPLVILAPHAVEALVKQPPDRWLSNARMTHYQALL
LDTDRVQFGPVVALNPATLLPLPEEGLQHNCLDILAEAHGTRPDLTDQPLPDADHTWYTDGSSLLQEGQR
KAGAAVTTETEVIWAKALPAGTSAQRAELIALTQALKMAEGKKLNVYTDSRYAFATAHIHGEIYRRRGWL

TSEGKEIKNKDEILALLKALFLPKRLSIIHCPGHQKGHSAEARGNRMADQAARKAAITETPDTSTLLIEN
SSPSGGSKRTADGSEFESPKKKRKVGSSLEVLFQ↓GPGSHHHHHH

Table S1. Primer sequences. All primers for Illumina MiSeq (next generation sequencing, NGS) were ordered with (5'-GCTCTTCCGATCT-3') at the 5' end for library preparation and indexing.

Primer name	Sequence (5'->3')	Application
AAVS1-1-fwd	ACGTAACCTGAGAAGGGAATCCCT	AAVS1-1 target (short read Illumina MiSeq)
AAVS1-1-rev	ATCCCCGTTCCCCTGCATCCCCCTT	AAVS1-1 target (short read Illumina MiSeq)
AAVS1-2-fwd	AGCTCAGGTTCTGGGAGAGGGTAG	AAVS1-2 target (short read Illumina MiSeq)
AAVS1-2-rev	TAGTCTCCTGATATTGGGTCTAAC	AAVS1-2 target (short read Illumina MiSeq)
AAVS1-3-fwd	TGGAAGATGCCATGACAGGGGGCT	AAVS1-3 target (short read Illumina MiSeq)
AAVS1-3-rev	TTGCGTCCCGCCTCCCCTTCTTGTA	AAVS1-3 target (short read Illumina MiSeq)
AAVS1-4-fwd	AGGACCGGCTGGAGGGGCTCAACAT	AAVS1-4 target (short read Illumina MiSeq)
AAVS1-4-rev	CCTTACCATTCCCCTTCGACCACC	AAVS1-4 target (short read Illumina MiSeq)
EMX1-1-fwd	CCTCTACATTCTACTTCTCTGTGT	EMX1-1 target (short read Illumina MiSeq)
EMX1-1-rev	TGCACCCTATCCTGGACTTCCTAA	EMX1-1 target (short read Illumina MiSeq)
EMX1-2-fwd	CTCAGGAACAAGGTCGCAGCTTCA	EMX1-2 target (short read Illumina MiSeq)
EMX1-2-rev	TTGGGGCTAAACTCCAACCCTCCCCT	EMX1-2 target (short read Illumina MiSeq)
EMX1-3-fwd	TCGGAGAGTTAATGTTCAGTGTGG	EMX1-3 target (short read Illumina MiSeq)
EMX1-3-rev	TGGCTACGACCTTAAAAGCCCTCT	EMX1-3 target (short read Illumina MiSeq)
EMX1-4-fwd	CACCTGGCCAATAGATGCTGACCT	EMX1-4 target (short read Illumina MiSeq)
EMX1-4-rev	GGACCAGGGTAGATTGCTCAATTTT	EMX1-4 target (short read Illumina MiSeq)
CFTR-G542-fwd	agaaggaagatgtgcctttcaaattcag	CFTR-G542 target (short read Illumina MiSeq)
CFTR-G542-rev	cagaatataaagcaatagagaaatgtctg	CFTR-G542 target (short read Illumina MiSeq)
CFTR-W1282-fwd	ttataggtgggcctcttgggaagaactgg	CFTR-W1282 target (short read Illumina MiSeq)
CFTR-W1282-rev	gcagagtaatatgaatttcttgagtac	CFTR-W1282 target (short read Illumina MiSeq)
BS-272-fwd	GCTCCTGGGCAACGTGCTGGTTATTG	PCR validation for editing at the tdTomato target
BS-273-rev	TTGATGACCTCCTCGCCCTTGCTCAC	PCR validation for editing at the tdTomato target
PCSK9-ext-fwd	agcatggggatgtggccaagttaag	PCSK9 target (extended amplicon)
PCSK9-ext-rev	gtaatgtgccctatggtggcagtca	PCSK9 target (extended amplicon)
PCSK9-fwd	ccaacaggtcactgctcatcttcaccaaga	PCSK9 target (short read Illumina MiSeq)
PCSK9-rev	gcatgctacactgagatgaggtcatgctggg	PCSK9 target (short read Illumina MiSeq)
SFTPC-ext-fwd	tagcgatggtgtctgctcgctcact	SFTPC target (extended amplicon)
SFTPC-ext-rev	aaaccatgaagctggcacatggact	SFTPC target (extended amplicon)
SFTPC-fwd	tgtcacccaagatgtgctcatacctgt	SFTPC target (short read Illumina MiSeq)
SFTPC-rev	acttgtctctctgcttcccttgctct	SFTPC target (short read Illumina MiSeq)

T1-OFT1-fwd	agctcatcttaaggtgctgtgtttacc	T1 off-target site 1 (short read Illumina MiSeq)
T1-OFT1-rev	tgagattggctcacctttcaggggct	T1 off-target site 1 (short read Illumina MiSeq)
T1-OFT2-fwd	taaagccagccaggcttgtgtcctt	T1 off-target site 2 (short read Illumina MiSeq)
T1-OFT2-rev	tttgagtccttgagtgggaagagtcc	T1 off-target site 2 (short read Illumina MiSeq)
T1-OFT3-fwd	tctgcttaataaacaatgtagtcctc	T1 off-target site 3 (short read Illumina MiSeq)
T1-OFT3-rev	cctgagattgtgaacaacatctatcg	T1 off-target site 3 (short read Illumina MiSeq)
T1-OFT4-fwd	tggaacctgccagaagcctggcatca	T1 off-target site 4 (short read Illumina MiSeq)
T1-OFT4-rev	ctaggattcaaacctacgtctggcaa	T1 off-target site 4 (short read Illumina MiSeq)
T1-OFT5-fwd	ccaaattccaaattttggttgcttc	T1 off-target site 5 (short read Illumina MiSeq)
T1-OFT5-rev	gatccctttggtgtgttgaggaatgc	T1 off-target site 5 (short read Illumina MiSeq)
T1-OFT6-fwd	gagggaatcttctcagcaaaagatag	T1 off-target site 6 (short read Illumina MiSeq)
T1-OFT6-rev	gttctcaaagtcccataatgtatgc	T1 off-target site 6 (short read Illumina MiSeq)
T1-OFT7-fwd	cccatgtgtgtacacttgtacacat	T1 off-target site 7 (short read Illumina MiSeq)
T1-OFT7-rev	gcatcccatcttgaatggaaatgaag	T1 off-target site 7 (short read Illumina MiSeq)
T1-OFT8-fwd	cctcagtagtaaaagaatttgtagg	T1 off-target site 8 (short read Illumina MiSeq)
T1-OFT8-rev	ctcctagaattatctcaagatctcc	T1 off-target site 8 (short read Illumina MiSeq)
T2-OFT1-fwd	acctccgatgtaggccacagagaag	T2 off-target site 1 (short read Illumina MiSeq)
T2-OFT1-rev	agctcctaccccaacaacatccgct	T2 off-target site 1 (short read Illumina MiSeq)
T2-OFT2-fwd	aagtaaaataaggcaactggaacac	T2 off-target site 2 (short read Illumina MiSeq)
T2-OFT2-rev	aggctgaggcagaagaatcgcttgaa	T2 off-target site 2 (short read Illumina MiSeq)
T2-OFT3-fwd	gatagaagcattttaagggctttca	T2 off-target site 3 (short read Illumina MiSeq)
T2-OFT3-rev	ccaaaatggatatattgcctccagcg	T2 off-target site 3 (short read Illumina MiSeq)
T2-OFT4-fwd	attcattgcagagggcgagagcctc	T2 off-target site 4 (short read Illumina MiSeq)
T2-OFT4-rev	gagttgtgcctgcatcatgctgatg	T2 off-target site 4 (short read Illumina MiSeq)
T2-OFT5-fwd	cgaaggaagggagagaaggagggaa	T2 off-target site 5 (short read Illumina MiSeq)
T2-OFT5-rev	atgagggcactctgctgggttcctta	T2 off-target site 5 (short read Illumina MiSeq)
T2-OFT6-fwd	taggaaaataaggtggcctgaggtg	T2 off-target site 6 (short read Illumina MiSeq)
T2-OFT6-rev	ctcctttggtcaatgcaagctctaa	T2 off-target site 6 (short read Illumina MiSeq)
T2-OFT7-fwd	tcattgcagagggcgagagcctcca	T2 off-target site 7 (short read Illumina MiSeq)
T2-OFT7-rev	agttgtgcctgcatcatgctgatgc	T2 off-target site 7 (short read Illumina MiSeq)
T3-OFT1-fwd	cccacatgctcaacactgctggaat	T3 off-target site 1 (short read Illumina MiSeq)
T3-OFT1-rev	agcttcttcttgctccaggaagtgg	T3 off-target site 1 (short read Illumina MiSeq)
T3-OFT2-fwd	aggagttcgagaccagcctggccaa	T3 off-target site 2 (short read Illumina MiSeq)

T3-OFT2-rev	agctatctcacaagtagaattggag	T3 off-target site 2 (short read Illumina MiSeq)
T3-OFT3-fwd	acatctacgccaaggtccgcaagaga	T3 off-target site 3 (short read Illumina MiSeq)
T3-OFT3-rev	caaatacgacctgtttcccaaacatg	T3 off-target site 3 (short read Illumina MiSeq)
T3-OFT4-fwd	gcactgagtcacatattctaagaaa	T3 off-target site 4 (short read Illumina MiSeq)
T3-OFT4-rev	caaaacctctctcaatcaagaacacg	T3 off-target site 4 (short read Illumina MiSeq)
T3-OFT5-fwd	cacacacacatatatatgtatacccac	T3 off-target site 5 (short read Illumina MiSeq)
T3-OFT5-rev	actcaccaagggggggacatgggtgaa	T3 off-target site 5 (short read Illumina MiSeq)
T3-OFT6-fwd	cacctgaggtcaggagttcaagagca	T3 off-target site 6 (short read Illumina MiSeq)
T3-OFT6-rev	gatctgtatggtcacttcttctatcc	T3 off-target site 6 (short read Illumina MiSeq)
T3-OFT7-fwd	agtctttccaggctaaggggttccat	T3 off-target site 7 (short read Illumina MiSeq)
T3-OFT7-rev	tatgctcatttacctcaagactgtcc	T3 off-target site 7 (short read Illumina MiSeq)
T3-OFT8-fwd	ggtttcattttgggaaagaaatcttc	T3 off-target site 8 (short read Illumina MiSeq)
T3-OFT8-rev	ggagttcgagaccagcctgaactaca	T3 off-target site 8 (short read Illumina MiSeq)
T3-OFT9-fwd	ccagcatggtctcaacatggcgaaa	T3 off-target site 9 (short read Illumina MiSeq)
T3-OFT9-rev	ctgcaatcaaaaaaaagagattggc	T3 off-target site 9 (short read Illumina MiSeq)

Table S2. sgRNA sequences (**Alt-R** modifications by IDT: 2'-*O* methylation and phosphorothioate linkage with the last 3 nucleotides at both 3'- and 5'-ends; m stands for 2'-*O* methylation, and * stands for phosphorothioate linkage; **spacer** sequences are shown in bold). *Note: by IVT – sgRNA prepared by *in vitro* transcription.

sgRNA name	Sequence (5'->3')	Alt-R
tdTom-g1	**UGUUUAUUGCAGCUUAUAAUG**GUCAUAGUUCCCCUGAgaaaUCAGGGUUACUAUGAUAAGGGCUUUCUGCCUAAGGCAGACUGACCCGCGGCGUUGGGGAUCGCCUGUCGCCCGCUUUUGGCGGGCAUUCCCCAUCCUU	No
tdTom-g2	**UAAAGCAAUAGCAUCACAAAU**GUCAUAGUUCCCCUGAgaaaUCAGGGUUACUAUGAUAAGGGCUUUCUGCCUAAGGCAGACUGACCCGCGGCGUUGGGGAUCGCCUGUCGCCCGCUUUUGGCGGGCAUUCCCCAUCCUU	No
tdTom-g3	**CACUGCAUUCUAGUUGUGGUU**GUCAUAGUUCCCCUGAgaaaUCAGGGUUACUAUGAUAAGGGCUUUCUGCCUAAGGCAGACUGACCCGCGGCGUUGGGGAUCGCCUGUCGCCCGCUUUUGGCGGGCAUUCCCCAUCCUU	No
tdTom-g4	**UUUUUUAAAGCAAGUAAAACC**GUCAUAGUUCCCCUGAgaaaUCAGGGUUACUAUGAUAAGGGCUUUCUGCCUAAGGCAGACUGACCCGCGGCGUUGGGGAUCGCCUGUCGCCCGCUUUUGGCGGGCAUUCCCCAUCCUU	No
tdTom-g5	**GGUUACAAAUAAAGCAAUAGC**GUCAUAGUUCCCCUGAgaaaUCAGGGUUACUAUGAUAAGGGCUUUCUGCCUAAGGCAGACUGACCCGCGGCGUUGGGGAUCGCCUGUCGCCCGCUUUUGGCGGGCAUUCCCCAUCCUU	No
tdTom-g6	**GAUAAGAUACAUUGAUGAGUU**GUCAUAGUUCCCCUGAgaaaUCAGGGUUACUAUGAUAAGGGCUUUCUGCCUAAGGCAGACUGACCCGCGGCGUUGGGGAUCGCCUGUCGCCCGCUUUUGGCGGGCAUUCCCCAUCCUU	No
tdTom-g7	**AUAACUUCGUAUAGCAUACAU**GUCAUAGUUCCCCUGAgaaaUCAGGGUUACUAUGAUAAGGGCUUUCUGCCUAAGGCAGACUGACCCGCGGCGUUGGGGAUCGCCUGUCGCCCGCUUUUGGCGGGCAUUCCCCAUCCUU	No
tdTom-g8	**AUAACUUCGUAUAAUGUAUGC**GUCAUAGUUCCCCUGAgaaaUCAGGGUUACUAUGAUAAGGGCUUUCUGCCUAAGGCAGACUGACCCGCGGCGUUGGGGAUCGCCUGUCGCCCGCUUUUGGCGGGCAUUCCCCAUCCUU	No
tdTom-g9	**UCUAGAGGAUCAUAAUCAGCC**GUCAUAGUUCCCCUGAgaaaUCAGGGUUACUAUGAUAAGGGCUUUCUGCCUAAGGCAGACUGACCCGCGGCGUUGGGGAUCGCCUGUCGCCCGCUUUUGGCGGGCAUUCCCCAUCCUU	No
tdTom-g10	**GUUUUACUUGCUUUAAAAAAC**GUCAUAGUUCCCCUGAgaaaUCAGGGUUACUAUGAUAAGGGCUUUCUGCCUAAGGCAGACUGACCCGCGGCGUUGGGGAUCGCCUGUCGCCCGCUUUUGGCGGGCAUUCCCCAUCCUU	No
tdTom-g11	**AAUAAAGCAAUAGCAUCACAA**GUCAUAGUUCCCCUGAgaaaUCAGGGUUACUAUGAUAAGGGCUUUCUGCCUAAGGCAGACUGACCCGCGGCGUUGGGGAUCGCCUGUCGCCCGCUUUUGGCGGGCAUUCCCCAUCCUU	No
tdTom-g12	**GAUACAUUGAUGAGUUUGGAC**GUCAUAGUUCCCCUGAgaaaUCAGGGUUACUAUGAUAAGGGCUUUCUGCCUAAGGCAGACUGACCCGCGGCGUUGGGGAUCGCCUGUCGCCCGCUUUUGGCGGGCAUUCCCCAUCCUU	No
tdTom-g13	**GGCUGAUUAUGAUCCUCUAGA**GUCAUAGUUCCCCUGAgaaaUCAGGGUUACUAUGAUAAGGGCUUUCUGCCUAAGGCAGACUGACCCGCGGCGUUGGGGAUCGCCUGUCGCCCGCUUUUGGCGGGCAUUCCCCAUCCUU	No
tdTom-g14	**UAGAGGAUCUGCGACUCUAGA**GUCAUAGUUCCCCUGAgaaaUCAGGGUUACUAUGAUAAGGGCUUUCUGCCUAAGGCAGACUGACCCGCGGCGUUGGGGAUCGCCUGUCGCCCGCUUUUGGCGGGCAUUCCCCAUCCUU	No
tdTom-g15	**GCGACUCUAGAGGAUCAUAAU**GUCAUAGUUCCCCUGAgaaaUCAGGGUUACUAUGAUAAGGGCUUUCUGCCUAAGGCAGACUGACCCGCGGCGUUGGGGAUCGCCUGUCGCCCGCUUUUGGCGGGCAUUCCCCAUCCUU	No
tdTom-g16	**CCCACACCUCCCCCUGAACCU**GUCAUAGUUCCCCUGAgaaaUCAGGGUUACUAUGAUAAGGGCUUUCUGCCUAAGGCAGACUGACCCGCGGCGUUGGGGAUCGCCUGUCGCCCGCUUUUGGCGGGCAUUCCCCAUCCUU	No
tdTom-g17	**GCAUUCUAGUUGUGGUUUGUC**GUCAUAGUUCCCCUGAgaaaUCAGGGUUACUAUGAUAAGGGCUUUCUGCCUAAGGCAGACUGACCCGCGGCGUUGGGGAUCGCCUGUCGCCCGCUUUUGGCGGGCAUUCCCCAUCCUU	No
tdTom-g18	**ACCUCCCACACCUCCCCCUGA**GUCAUAGUUCCCCUGAgaaaUCAGGGUUACUAUGAUAAGGGCUUUCUGCCUAAGGCAGACUGACCCGCGGCGUUGGGGAUCGCCUGUCGCCCGCUUUUGGCGGGCAUUCCCCAUCCUU	No
tdTom-g19	**GGACAAACCACAACUAGAAUG**GUCAUAGUUCCCCUGAgaaaUCAGGGUUACUAUGAUAAGGGCUUUCUGCC	No

	UAAGGCAGACUGACCCGCGGCGUUGGGGAUCGCCUGUCGCCCGCUUUUGGCGGGCAUUCCCCAUCCUU	
tdTom-g20	**GCUUUAUUUGUAACCAUUAUA**GUCAUAGUUCCCCUGAgaaaUCAGGGUUACUAUGAUAAGGGCUUUCUGCC<br>UAAGGCAGACUGACCCGCGGCGUUGGGGAUCGCCUGUCGCCCGCUUUUGGCGGGCAUUCCCCAUCCUU	No
tdTom-g21	**CCUGAACCUGAAACAUAAAAU**GUCAUAGUUCCCCUGAgaaaUCAGGGUUACUAUGAUAAGGGCUUUCUGCC<br>UAAGGCAGACUGACCCGCGGCGUUGGGGAUCGCCUGUCGCCCGCUUUUGGCGGGCAUUCCCCAUCCUU	No
tdTom-g22	**UAUUGCAGCUUAUAAUGGUUA**GUCAUAGUUCCCCUGAgaaaUCAGGGUUACUAUGAUAAGGGCUUUCUGCC<br>UAAGGCAGACUGACCCGCGGCGUUGGGGAUCGCCUGUCGCCCGCUUUUGGCGGGCAUUCCCCAUCCUU	No
tdTom-g3(ms)	**mC*mA*mC*UGCAUUCUAGUUGUGGUU**GUCAUAGUUCCCCUGAgaaaUCAGGGUUACUAUGAUAAGGGCUU<br>UCUGCCUAAGGCAGACUGACCCGCGGCGUUGGGGAUCGCCUGUCGCCCGCUUUUGGCGGGCAUUCCCCAUC<br>*mC*mU*mU	**Yes**
tdTom-g3(22ms)	**mU*mC*mA*CUGCAUUCUAGUUGUGGUU**GUCAUAGUUCCCCUGAgaaaUCAGGGUUACUAUGAUAAGGGCU<br>UUCUGCCUAAGGCAGACUGACCCGCGGCGUUGGGGAUCGCCUGUCGCCCGCUUUUGGCGGGCAUUCCCCAU<br>C*mC*mU*mU	**Yes**
tdTom-g3(23ms)	**mU*mU*mC*ACUGCAUUCUAGUUGUGGUU**GUCAUAGUUCCCCUGAgaaaUCAGGGUUACUAUGAUAAGGGC<br>UUUCUGCCUAAGGCAGACUGACCCGCGGCGUUGGGGAUCGCCUGUCGCCCGCUUUUGGCGGGCAUUCCCCA<br>UC*mC*mU*mU	**Yes**
tdTom-g3(24ms)	**mU*mU*mU*CACUGCAUUCUAGUUGUGGUU**GUCAUAGUUCCCCUGAgaaaUCAGGGUUACUAUGAUAAGGG<br>CUUUCUGCCUAAGGCAGACUGACCCGCGGCGUUGGGGAUCGCCUGUCGCCCGCUUUUGGCGGGCAUUCCCC<br>AUC*mC*mU*mU	**Yes**
EGFP-g1	**ACGACUUCUUCAAGUCCGCCA**GUCAUAGUUCCCCUGAgaaaUCAGGGUUACUAUGAUAAGGGCUUUCUGCC<br>UAAGGCAGACUGACCCGCGGCGUUGGGGAUCGCCUGUCGCCCGCUUUUGGCGGGCAUUCCCCAUCCUU	No
EGFP-g2	**ACCUGAGCACCCAGUCCAAGC**GUCAUAGUUCCCCUGAgaaaUCAGGGUUACUAUGAUAAGGGCUUUCUGCC<br>UAAGGCAGACUGACCCGCGGCGUUGGGGAUCGCCUGUCGCCCGCUUUUGGCGGGCAUUCCCCAUCCUU	No
EGFP-g3	**CAAGCUGGAGUACAACUACAA**GUCAUAGUUCCCCUGAgaaaUCAGGGUUACUAUGAUAAGGGCUUUCUGCC<br>UAAGGCAGACUGACCCGCGGCGUUGGGGAUCGCCUGUCGCCCGCUUUUGGCGGGCAUUCCCCAUCCUU	No
EGFP-g4	**GGGCACGGGCAGCUUGCCGGU**GUCAUAGUUCCCCUGAgaaaUCAGGGUUACUAUGAUAAGGGCUUUCUGCC<br>UAAGGCAGACUGACCCGCGGCGUUGGGGAUCGCCUGUCGCCCGCUUUUGGCGGGCAUUCCCCAUCCUU	No
EGFP-g5	**UUGAAGAAGAUGGUGCGCUCC**GUCAUAGUUCCCCUGAgaaaUCAGGGUUACUAUGAUAAGGGCUUUCUGCC<br>UAAGGCAGACUGACCCGCGGCGUUGGGGAUCGCCUGUCGCCCGCUUUUGGCGGGCAUUCCCCAUCCUU	No
EGFP-g6	**AUGCGGUUCACCAGGGUGUCG**GUCAUAGUUCCCCUGAgaaaUCAGGGUUACUAUGAUAAGGGCUUUCUGCC<br>UAAGGCAGACUGACCCGCGGCGUUGGGGAUCGCCUGUCGCCCGCUUUUGGCGGGCAUUCCCCAUCCUU	No
EGFP-g6(23ms)	**mC*mG*mA*UGCGGUUCACCAGGGUGUCG**GUCAUAGUUCCCCUGAgaaaUCAGGGUUACUAUGAUAAGGGC<br>UUUCUGCCUAAGGCAGACUGACCCGCGGCGUUGGGGAUCGCCUGUCGCCCGCUUUUGGCGGGCAUUCCCCA<br>UC*mC*mU*mU	**Yes**
rEGFP-R1	**mC*mG*mC*GAAAAGCAUUGUACGCCAU**GUCAUAGUUCCCCUGAgaaaUCAGGGUUACUAUGAUAAGGGCU<br>UUCUGCCUAAGGCAGACUGACCCGCGGCGUUGGGGAUCGCCUGUCGCCCGCUUUUGGCGGGCAUUCCCCAU<br>C*mC*mU*mU	**Yes**
rEGFP-R2	**mU*mG*mU*ACGCCAUAUGUCAAAGUGG**GUCAUAGUUCCCCUGAgaaaUCAGGGUUACUAUGAUAAGGGCU<br>UUCUGCCUAAGGCAGACUGACCCGCGGCGUUGGGGAUCGCCUGUCGCCCGCUUUUGGCGGGCAUUCCCCAU<br>C*mC*mU*mU	**Yes**
rEGFP-R3	**mC*mA*mC*UUUGGUGACCACUUUGACAU**GUCAUAGUUCCCCUGAgaaaUCAGGGUUACUAUGAUAAGGGC<br>UUUCUGCCUAAGGCAGACUGACCCGCGGCGUUGGGGAUCGCCUGUCGCCCGCUUUUGGCGGGCAUUCCCCA<br>UC*mC*mU*mU	**Yes**
rEGFP-R4	**mU*mU*mG*GUGACCACUUUGACAUAUGG**GUCAUAGUUCCCCUGAgaaaUCAGGGUUACUAUGAUAAGGGC<br>UUUCUGCCUAAGGCAGACUGACCCGCGGCGUUGGGGAUCGCCUGUCGCCCGCUUUUGGCGGGCAUUCCCCA	**Yes**

	UC*mC*mU*mU	
AAVS1-g1	**mA*mC*mC*CCACAGUUGGAGGAGAAUC**GUCAUAGUUCCCCUGAgaaaUCAGGGUUACUAUGAUAAGGGCUUUCUGCCUAAGGCAGACUGACCCGCGGCGUUGGGGAUCGCCUGUCGCCCGCUUUUGGCGGGCAUUCCCCAUC*mC*mU*mU	**Yes**
AAVS1-g2	**mA*mG*mG*AUCCUCUCUGGCUCCAUCG**GUCAUAGUUCCCCUGAgaaaUCAGGGUUACUAUGAUAAGGGCUUUCUGCCUAAGGCAGACUGACCCGCGGCGUUGGGGAUCGCCUGUCGCCCGCUUUUGGCGGGCAUUCCCCAUC*mC*mU*mU	**Yes**
AAVS1-g3	**mA*mU*mC*CACAGGAGAACGGGGUGUC**GUCAUAGUUCCCCUGAgaaaUCAGGGUUACUAUGAUAAGGGCUUUCUGCCUAAGGCAGACUGACCCGCGGCGUUGGGGAUCGCCUGUCGCCCGCUUUUGGCGGGCAUUCCCCAUC*mC*mU*mU	**Yes**
AAVS1-g4	**mG*mC*mG*ACUCCAAUGCGGAAGAGAGU**GUCAUAGUUCCCCUGAgaaaUCAGGGUUACUAUGAUAAGGGCUUUCUGCCUAAGGCAGACUGACCCGCGGCGUUGGGGAUCGCCUGUCGCCCGCUUUUGGCGGGCAUUCCCCAUC*mC*mU*mU	**Yes**
EMX1-g5	**mG*mU*mU*AUUACUUAAUUAUCUGCCA**GUCAUAGUUCCCCUGAgaaaUCAGGGUUACUAUGAUAAGGGCUUUCUGCCUAAGGCAGACUGACCCGCGGCGUUGGGGAUCGCCUGUCGCCCGCUUUUGGCGGGCAUUCCCCAUC*mC*mU*mU	**Yes**
EMX1-g6	**mU*mU*mC*GGGAAAACCAAUGUGUUGG**GUCAUAGUUCCCCUGAgaaaUCAGGGUUACUAUGAUAAGGGCUUUCUGCCUAAGGCAGACUGACCCGCGGCGUUGGGGAUCGCCUGUCGCCCGCUUUUGGCGGGCAUUCCCCAUC*mC*mU*mU	**Yes**
EMX1-g7	**mC*mC*mA*AGGUGGGGAAGGUUUGCAG**GUCAUAGUUCCCCUGAgaaaUCAGGGUUACUAUGAUAAGGGCUUUCUGCCUAAGGCAGACUGACCCGCGGCGUUGGGGAUCGCCUGUCGCCCGCUUUUGGCGGGCAUUCCCCAUC*mC*mU*mU	**Yes**
EMX1-g8	**mU*mU*mU*CAAAGACUUGCCCAAAUCCA**GUCAUAGUUCCCCUGAgaaaUCAGGGUUACUAUGAUAAGGGCUUUCUGCCUAAGGCAGACUGACCCGCGGCGUUGGGGAUCGCCUGUCGCCCGCUUUUGGCGGGCAUUCCCCAUC*mC*mU*mU	**Yes**
CFTR-g1	**mC*mC*mU*CCACUCAGUGUGAUUCCACC**GUCAUAGUUCCCCUGAgaaaUCAGGGUUACUAUGAUAAGGGCUUUCUGCCUAAGGCAGACUGACCCGCGGCGUUGGGGAUCGCCUGUCGCCCGCUUUUGGCGGGCAUUCCCCAUC*mC*mU*mU	**Yes**
CFTR-g2	**mC*mA*mC*UCCAAAGGCUUUCCUUCACU**GUCAUAGUUCCCCUGAgaaaUCAGGGUUACUAUGAUAAGGGCUUUCUGCCUAAGGCAGACUGACCCGCGGCGUUGGGGAUCGCCUGUCGCCCGCUUUUGGCGGGCAUUCCCCAUC*mC*mU*mU	**Yes**
HM-tdTom-g3(23)	**mU*mU*mC*ACUGCAUUCUAGUUGUGGUU**GUCAUAGUUCCCmCmUmGmAmgmamamamUmCmAmGGGUUACUAUGAUAAGGGCUmUmUmCmUmGmCmCmUmAmAmGmGmCmAmGmAmCUGACCCGCGGCGUUGGGGAUCGCCUGUCGmCmCmCmGmCmUmUmUmUGGCGGGCAUmUCCCCAUC*mC*mU*mU	**Yes**
HM-tdTom-g7(23)	**mG*mA*mA*UAACUUCGUAUAGCAUACAU**GUCAUAGUUCCCmCmUmGmAmgmamamamUmCmAmGGGUUACUAUGAUAAGGGCUmUmUmCmUmGmCmCmUmAmAmGmGmCmAmGmAmCUGACCCGCGGCGUUGGGGAUCGCCUGUCGmCmCmCmGmCmUmUmUmUGGCGGGCAUmUCCCCAUC*mC*mU*mU	**Yes**
UM-tdTom-g3(23)	**GUUCACUGCAUUCUAGUUGUGGUU**GUCAUAGUUCCCCUGAgaaaUCAGGGUUACUAUGAUAAGGGCUUUCUGCCUAAGGCAGACUGACCCGCGGCGUUGGGGAUCGCCUGUCGCCCGCUUUUGGCGGGCAUUCCCCAUCCUU	By IVT
UM-tdTom-g7(23)	**GAAUAACUUCGUAUAGCAUACAU**GUCAUAGUUCCCCUGAgaaaUCAGGGUUACUAUGAUAAGGGCUUUCUGCCUAAGGCAGACUGACCCGCGGCGUUGGGGAUCGCCUGUCGCCCGCUUUUGGCGGGCAUUCCCCAUCCUU	By IVT
UM-gPCSK9	**GAGGAGACCCAGAGGCUACAGAU**GUCAUAGUUCCCCUGAgaaaUCAGGGUUACUAUGAUAAGGGCUUUCUGCCUAAGGCAGACUGACCCGCGGCGUUGGGGAUCGCCUGUCGCCCGCUUUUGGCGGGCAUUCCCCAUCCUU	By IVT
UM-gSFTPC	**GCUCUGCUCAUGGGCCUCCACAU**GUCAUAGUUCCCCUGAgaaaUCAGGGUUACUAUGAUAAGGGCUUUCUGCCUAAGGCAGACUGACCCGCGGCGUUGGGGAUCGCCUGUCGCCCGCUUUUGGCGGGCAUUCCCCAUCCUU	By IVT
SpyCas9-	**mA*mA*mG*UAAAACCUCUACAAAUG**GUUUUAGAGCUAGAAAUAGCAAGUUAAAAUAAGGCUAGUCCGUUA	**Yes**

tdTom (g9 in Fig S4a)	UCAACUUGAAAAAGUGGCACCGAGUCGGUGCmU*mU*mU*U	
Spy-tdTom-g1	**mC*mA*mG*CCAUACCACAUUUGUAG**GUUUUAGAGCUAGAAAUAGCAAGUUAAAAUAAGGCUAGUCCGUUA<br>UCAACUUGAAAAAGUGGCACCGAGUCGGUGCmU*mU*mU*U	**Yes**
Spy-tdTom-g2	**mU*mC*mA*AUGUAUCUUAUCAUGUC**GUUUUAGAGCUAGAAAUAGCAAGUUAAAAUAAGGCUAGUCCGUUA<br>UCAACUUGAAAAAGUGGCACCGAGUCGGUGCmU*mU*mU*U	**Yes**
Spy-tdTom-g3	**mU*mU*mU*UCACUGCAUUCUAGUUG**GUUUUAGAGCUAGAAAUAGCAAGUUAAAAUAAGGCUAGUCCGUUA<br>UCAACUUGAAAAAGUGGCACCGAGUCGGUGCmU*mU*mU*U	**Yes**
Spy-tdTom-g4	**mU*mA*mU*GUUUCAGGUUCAGGGGG**GUUUUAGAGCUAGAAAUAGCAAGUUAAAAUAAGGCUAGUCCGUUA<br>UCAACUUGAAAAAGUGGCACCGAGUCGGUGCmU*mU*mU*U	**Yes**
Spy-tdTom-g5	**mA*mA*mA*CCUCUACAAAUGUGGUA**GUUUUAGAGCUAGAAAUAGCAAGUUAAAAUAAGGCUAGUCCGUUA<br>UCAACUUGAAAAAGUGGCACCGAGUCGGUGCmU*mU*mU*U	**Yes**
Spy-tdTom-g6	**mU*mU*mG*UUUAUUGCAGCUUAUAA**GUUUUAGAGCUAGAAAUAGCAAGUUAAAAUAAGGCUAGUCCGUUA<br>UCAACUUGAAAAAGUGGCACCGAGUCGGUGCmU*mU*mU*U	**Yes**
Spy-tdTom-g7	**mU*mU*mU*UAUGUUUCAGGUUCAGG**GUUUUAGAGCUAGAAAUAGCAAGUUAAAAUAAGGCUAGUCCGUUA<br>UCAACUUGAAAAAGUGGCACCGAGUCGGUGCmU*mU*mU*U	**Yes**
Spy-tdTom-g8	**mA*mU*mA*AGAUACAUUGAUGAGUU**GUUUUAGAGCUAGAAAUAGCAAGUUAAAAUAAGGCUAGUCCGUUA<br>UCAACUUGAAAAAGUGGCACCGAGUCGGUGCmU*mU*mU*U	**Yes**
Spy-tdTom-g10	**mA*mU*mU*UUAUGUUUCAGGUUCAG**GUUUUAGAGCUAGAAAUAGCAAGUUAAAAUAAGGCUAGUCCGUUA<br>UCAACUUGAAAAAGUGGCACCGAGUCGGUGCmU*mU*mU*U	**Yes**
LbCas12a-tdTom	mU*mA*mA*UUUCUACUAAGUGUAGAU**UUGCAGCUUAUAAUGGUUACA*mA*mA*mU**	**Yes**
Spy-EGFP-g1	**mA*mG*mC*ACTGCACGCCATAGGTC**GUUUUAGAGCUAGAAAUAGCAAGUUAAAAUAAGGCUAGUCCGUUA<br>UCAACUUGAAAAAGUGGCACCGAGUCGGUGCmU*mU*mU*U	**Yes**
pegRNA-GtB	**mG*mG*mC*UGAAGCACUGCACGCCAU**GUUUUAGAGCUAGAAAUAGCAAGUUAAAAUAAGGCUAGUCCGUU<br>AUCAACUUGAAAAAGUGGCACCGAGUCGGUGCUGACCACCCUGA**G**C**CAC**GGCGUGCAGUGCUUC*mA*mG*<br>mC	**Yes**

Table S3. ssDNA sequences (HDR templates and enhancer ssDNA with **Alt-R** modifications by IDT; * stands for phosphorothioate linkage).

ssDNA name	Sequence (5'->3')
rEGFP-RD1	g*g*g*cgatgccacctacggcaagctgaccctgaagttcatctgcaccaccggcaagctgcccgtgccctggccca<br>ctttggtgaccactttAaGCCaCggAgtacaatgcttttcgcgctaccccgaccacatgaagcagcacgacttcttc<br>aagtccgccatgcccgaaggctacgtccaggag*c*g*c
rEGFP-RD2	g*c*g*ctcctggacgtagccttcgggcatggcggacttgaagaagtcgtgctgcttcatgtggtcggggtagcgcg<br>aaaagcattgtacTccGtGGCtTaaagtggtcaccaaagtgggccagggcacgggcagcttgccggtggtgcagatg<br>aacttcagggtcagcttgccgtaggtggcatcg*c*c*c
rEGFP-RD3	g*g*g*cgatgccacctacggcaagctgaccctgaagttcatctgcaccaccggcaagctgcccgtgccctggccca<br>ctttggtgaccactttAacTCaCggAgtacaatgcttttcgcgctaccccgaccacatgaagcagcacgacttcttc<br>aagtccgccatgcccgaaggctacgtccaggag*c*g*c
rEGFP-RD4	g*c*g*ctcctggacgtagccttcgggcatggcggacttgaagaagtcgtgctgcttcatgtggtcggggtagcgcg<br>aaaagcattgtacTccGtGAgtTaaagtggtcaccaaagtgggccagggcacgggcagcttgccggtggtgcagatg<br>aacttcagggtcagcttgccgtaggtggcatcg*c*c*c
AAVS1-D1	t*c*t*ctgaaccccagcccaccccaatgctccaggcctcctgggataccccgaagagtgagtttgccaagcagtca<br>ccccacagttggaggaAGCTTcacccaaaaggcagcctggtagacagggctggggtggcctctcgtggggtccaggc<br>caagtaggtggcctggggcctctgggggatg*c*a*g
AAVS1-D2	g*g*g*tggccactgagaaccgggcaggtcacgcatcccccccttccctcccacccccctgccaagctctccctccca<br>ggatcctctctggctcAAGCTtaagcaaaccttagaggttctggcaaggagagagatggctccaggaaatgggggtg<br>tgtcaccagataaggaatctgcctaacagga*g*g*t
AAVS1-D3	g*a*g*ctagcacagactagagaggtaagggggggtaggggagctgcccaaatgaaaggagtgagaggtgacccgaat<br>ccacaggagaacCAAGCTtccaggcaaagaaagcaagaggatggagaggtggctaaagccagggagacggggtactt<br>tggggttgtccagaaaaacggtgatgatgcag*g*c*c
AAVS1-D4	c*g*g*aagaggggaagtcgagggagggatggtaaggaggactgcatgggtcagcacaggctgccaaagccagggcc<br>agttaaagcgactccaatgcggaagCTTACaggtcgaaggggaatggtaaggaggcctggggcagagtggtcagcac<br>agagtggctaagcccagggccagttgaagcggctccaattcggaag*t*g*g
EMX1-D1	t*g*t*atactacctcctccacattctcagagctcaccacacaacccctgcctatcatgatatgcatcaaactttgt<br>tgttattacttaattaAGCTTGatgtccaaacatcaatctgtagaccagcagtatgcgtctctcagggagatcttaa<br>aatacagattcctgggtttcacccaggagatt*c*t*c
EMX1-D2	c*t*t*cagaccgcggcccaggaggccgatggtgggtgagtgggagagtcccggagagcagggggcagagagctgg<br>ttttcgggaaaaccaatgtAAGCTTccccaaacatccaccctccgctcggatccaagttctctgagaactgaaacga<br>catcccgggacgaatgggagagttaggctgagctacaca*c*c*g
EMX1-D3	g*g*g*cctggctgtcttgggatgtttctcggcaaccttggcccgacttctccaagtcacacgtgcctctcctaccc<br>aaggtggggaaggtAAgcTTtaagcaaactggcttccgccgttgctcgccgccttcgggagggagcccacccggctg<br>ctggaataccgaggacagttttcccgggcag*g*g*g
EMX1-D4	a*c*c*tggcagataccacaaaaccagagaatgtaattactagaataagaattgttgtgggtagccttgcctcctct<br>ttgaagatttcaaagacttgcccaaGCTTTaatccgaaaaaacaaaaatgctacaatgtcatctgccttgggcaaga<br>gtttctgccacttaaaaataaatgtttactgataacatgaggatat*c*t*t
CFTR-G542-D1	a*a*t*tcagattgagcatactaaaagtgactctctaattttctatttttggtaataggacatctccaagtttgcag<br>agaaagacaatatagttcttGgagaaggAggaatTacactgagtggaggtcaacgagcaagaatttctttagcaagg<br>tgaataactaattattggtctagcaagcatttgctgtaaatgtcat*t*c*a
CFTR-G542-D2	t*g*a*atgacatttacagcaaatgcttgctagaccaataattagttattcaccttgctaaagaaattcttgctcgt<br>tgacctccactcagtgtAattccTccttctcCaagaactatattgtctttctctgcaaacttggagatgtcctatta<br>ccaaaaatagaaaattagagagtcacttttagtatgctcaatctga*a*t*t
CFTR-W1282-D3	a*t*c*agggaagagtactttgttatcagctttttttgagactactgaacactgaaggagaaatccagatcgatggtg

	tgtcttgggattcaataactttAcaacaAtgGaggaaagcctttggagtgataccacaggtgagcaaaaggacttag ccagaaaaaaggcaactaaattatatttttttactgctatttgatac*t*t*g
CFTR-W1282-D4	c*a*a*gtatcaaatagcagtaaaaaatataatttagttgccttttttctggctaagtccttttgctcacctgtggt atcactccaaaggctttcctCcaTtgttgTaaagttattgaatcccaagacacaccatcgatctggatttctccttc agtgttcagtagtctcaaaaaagctgataacaaagtactcttccct*g*a*t
Enhancer ssDNA (enhDNA, 100 nt)	t*t*a*gctctgtttacgtcccagcgggcatgagagtaacaagagggtgtggtaatattacggtaccgagcactatc gatacaatatgtgtcatacggac*a*c*g
ssDNA (200 nt, for Figs. S11 & S21)	a*g*g*gcgagggcgatgccacctacggcaagctgaccctgaagttcatctgcaccaccggcaagctgcccgtgccc tggcccactttggtgaccactttaacttggggagtacaatgcttttcgcgctaccccgaccacatgaagcagcacga cttcttcaagtccgccatgcccgaaggctacgtccaggagcgcacc*a*t*c
